# PP2A^Cdc55^ Phosphatase Imposes Ordered Cell-Cycle Phosphorylation by Opposing Threonine Phosphorylation

**DOI:** 10.1016/j.molcel.2016.12.018

**Published:** 2017-02-02

**Authors:** Molly Godfrey, Sandra A. Touati, Meghna Kataria, Andrew Jones, Ambrosius P. Snijders, Frank Uhlmann

**Affiliations:** 1 Chromosome Segregation Laboratory, The Francis Crick Institute, London NW1 1AT, UK; 2 Mass Spectrometry Proteomics Science Technology Platform, The Francis Crick Institute, London NW1 1AT, UK

**Keywords:** cell cycle, cyclin-dependent kinase, PP2A phosphatase, *Saccharomyces cerevisiae*, phosphoproteome analysis

## Abstract

In the quantitative model of cell-cycle control, progression from G1 through S phase and into mitosis is ordered by thresholds of increasing cyclin-dependent kinase (Cdk) activity. How such thresholds are read out by substrates that respond with the correct phosphorylation timing is not known. Here, using the budding yeast model, we show that the abundant PP2A^Cdc55^ phosphatase counteracts Cdk phosphorylation during interphase and delays phosphorylation of late Cdk substrates. PP2A^Cdc55^ specifically counteracts phosphorylation on threonine residues, and consequently, we find that threonine-directed phosphorylation occurs late in the cell cycle. Furthermore, the late phosphorylation of a model substrate, Ndd1, depends on threonine identity of its Cdk target sites. Our results support a model in which Cdk-counteracting phosphatases contribute to cell-cycle ordering by imposing Cdk thresholds. They also unveil a regulatory principle based on the phosphoacceptor amino acid, which is likely to apply to signaling pathways beyond cell-cycle control.

## Highlights

• The phosphatase PP2A^Cdc55^ counteracts Cdk phosphorylation during the cell cycle
• It does so specifically on threonine, but not serine, phosphorylation sites
• Consequently, threonine phosphorylation is a late event in the cell cycle
• Thereby, PP2A^Cdc55^ contributes to setting the timing of mitosis

## Graphical Abstract

**Figure:**
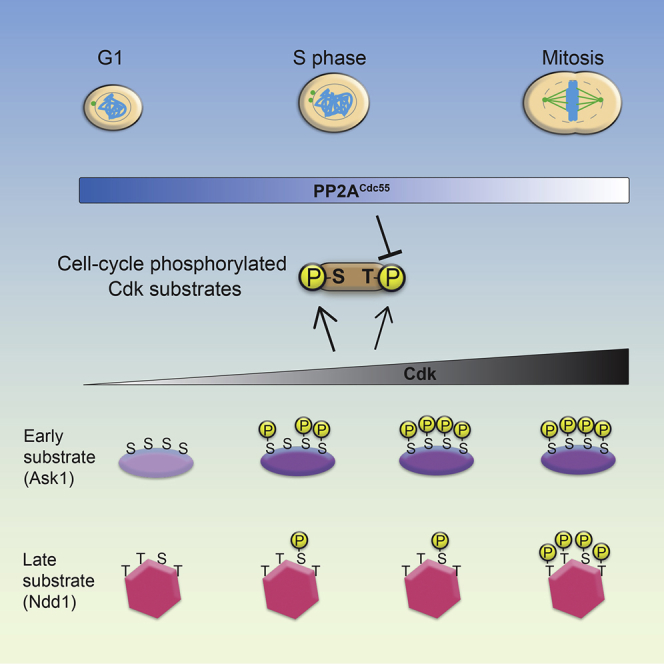

## Teaser

The eukaryotic cell cycle comprises a robustly ordered series of phosphorylation events. Godfrey et al. show that the phosphatase PP2A^Cdc55^ counteracts and thereby delays cell-cycle phosphorylation by cyclin-dependent kinase specifically on threonine, but not serine, phosphorylation sites. This reveals an ordering principle that might be also relevant in other signaling networks.

## Introduction

The cell-division cycle is made up of an ordered series of events that ensure faithful replication and accurate partitioning of the genetic material. A major impetus for cell-cycle progression stems from the phosphorylation of numerous target proteins by the master cyclin-dependent kinase (Cdk) (Morgan, 2007). Cdk substrates whose phosphorylation triggers DNA replication (Tanaka et al., 2007, Zegerman and Diffley, 2007), as well as many substrates that are important for entry into and progression through mitosis (Errico et al., 2010, Ubersax et al., 2003), are known, but how the correct substrate phosphorylation timing is defined, such that DNA replication occurs before mitosis, is not well understood. Sequential ordering of phosphorylation events is in part thought to arise from transcriptional waves of G1-, S phase-, and mitosis-specific cyclins that confer substrate specificity to the kinase. For example, G1- and S phase-specific cyclins recognize hydrophobic substrate motifs that increase kinase-substrate affinity and thereby enhance the phosphorylation rate of certain substrates (Bhaduri and Pryciak, 2011, Brown et al., 1999, Kõivomägi et al., 2011).

While cyclin specificity provides an apparently straightforward ordering principle, cell-cycle progression is remarkably resilient to its abolition. For example, the ordering of S phase and mitosis remains uncompromised if S phase cyclins are replaced by mitotic cyclins in both budding yeast and vertebrates (Hu and Aparicio, 2005, Moore et al., 2003). In another example, all budding yeast G1-specific cyclins can be removed so long as their role in upregulating S phase cyclin expression is compensated for (Tyers, 1996). What is more, ordered cell-cycle progression is maintained with a single source of cyclin-Cdk activity in fission yeast (Coudreuse and Nurse, 2010, Fisher and Nurse, 1996). This has led to the proposal that the quantitative increase, rather than qualitative differences, in Cdk activity controls ordered Cdk substrate phosphorylation (Stern and Nurse, 1996). How such a quantitative increase of Cdk activity is read out by substrates that respond by phosphorylation at the correct time is not known. In budding yeast, Cdk phosphorylates its many substrates with widely varying efficiencies (Loog and Morgan, 2005), but there is no obvious correlation between phosphorylation efficiency and phosphorylation timing. Thus, ordered cell-cycle phosphorylation is not simply defined by kinase affinity for its substrates, as the best substrates are not necessarily those that are phosphorylated first.

If Cdk activity is chemically inhibited at various cell-cycle stages, Cdk substrate phosphorylation is rapidly lost (Holt et al., 2009, Liakopoulos et al., 2003). This suggests that potent Cdk-counteracting phosphatase(s) are active in cells at most times. By reversing phosphorylation, such Cdk-counteracting phosphatases could set thresholds that Cdk activity must overcome to achieve net substrate phosphorylation (Uhlmann et al., 2011). Whether and how Cdk-counteracting phosphatases indeed act during interphase to order cell-cycle phosphorylation is not yet known. It is also unknown how such phosphatases might differentiate early from late phosphorylated Cdk targets to set a higher Cdk threshold for phosphorylation of the latter. Here, we analyze the contribution of the budding yeast PP2A^Cdc55^ phosphatase to the dynamics of cell-cycle phosphorylation. We find that PP2A^Cdc55^ indeed counteracts and thereby delays Cdk phosphorylation, but only on a subset of substrates. These are enriched for threonines as the phosphoacceptor sites, consistent with the known biochemical PP2A substrate preference. Our results portray cell-cycle phosphorylation as a dynamic process in which the steady-state level of phosphorylation is under control of the changing activity ratio of Cdk and its counteracting phosphatases.

## Results

### PP2A^Cdc55^ Phosphatase Imposes Late Cdk Substrate Phosphorylation

**Figure 1:**
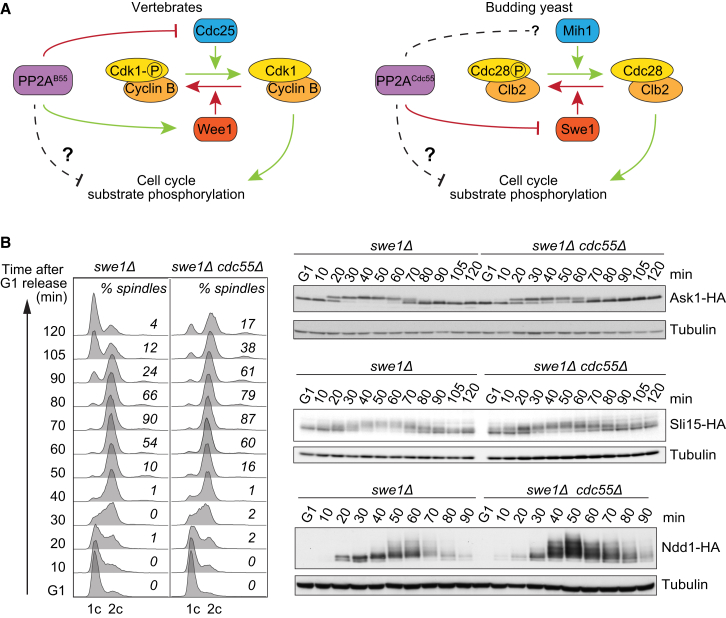
PP2A^Cdc55^ Delays Cdk Substrate Phosphorylation Independently of Impacting Cdk Tyrosine Phosphorylation (A) Simplified schematics of how PP2A^Cdc55^ is thought to affect Cdk activity in vertebrates and budding yeast (Chica et al., 2016, Harvey et al., 2011, Kinoshita et al., 1993, Lee et al., 1991, Minshull et al., 1996). See also Figure S1 for a comparison of cell-cycle progression in wild-type and *swe1Δ* strains. (B) PP2A^Cdc55^ delays cell-cycle phosphorylation of a subset of Cdk substrates. *swe1Δ* and *swe1Δ cdc55Δ* cells were arrested in G1 by pheromone α-factor treatment and released to progress through a synchronous cell cycle before re-arrest in the next G1 phase by α-factor re-addition. Protein extracts were prepared at the indicated times from strains in which Ask1, Sli15, or Ndd1 was fused to a hemagglutinin (HA) epitope tag. Cell-cycle progression was monitored by fluorescence-activated cell sorting (FACS) analysis of DNA content. Shown is a representative profile of the Sli15-HA experiment, as well as representative scores of cells containing mitotic (short or long) spindles. See also Figure S2A for FACS analyses of the other experiments, Figure S2B for a repeat of the Ndd1 phosphorylation analysis, including internal timing controls, and Figure S3 for cell-cycle phosphorylation analysis using antibodies raised against phosphorylated Cdk consensus peptides.

We analyzed a possible contribution of the abundant PP2A^Cdc55^ phosphatase to the regulation of Cdk substrate phosphorylation during the budding yeast cell cycle. PP2A^Cdc55^ and its orthologs in vertebrates have been studied for their impact on cell-cycle progression by controlling inhibitory Cdk tyrosine phosphorylation (Chica et al., 2016, Harvey et al., 2011, Kinoshita et al., 1993, Lee et al., 1991, Minshull et al., 1996) (Figure 1A). Budding yeast cells lacking PP2A^Cdc55^ show gross morphological defects and poor growth due to attenuated Cdk activity (Wang and Burke, 1997). To disambiguate a possible role of PP2A^Cdc55^ in dephosphorylating Cdk substrates, we therefore employed a budding yeast strain background lacking the Cdk tyrosine kinase Swe1 (*swe1Δ*). Without inhibitory Cdk phosphorylation, *swe1Δ* cells enter mitosis slightly earlier than wild-type cells but show otherwise undisturbed ordering of cell-cycle events (Harvey and Kellogg, 2003) (Figure S1, available online). Notably, absence of PP2A^Cdc55^ no longer causes morphological defects in cells lacking Swe1 (Wang and Burke, 1997).

To examine the impact of PP2A^Cdc55^ on the kinetics of Cdk substrate phosphorylation, we compared the electrophoretic mobility changes of three known Cdk targets, Ask1, Sli15, and Ndd1 (Li and Elledge, 2003, Pereira and Schiebel, 2003, Reynolds et al., 2003), during synchronous cell-cycle progression of *swe1Δ* and *swe1Δ cdc55Δ* cells (Figure 1B). Ask1 is phosphorylated early in the cell cycle as soon as cells enter S phase. Its phosphorylation timing was unaltered in the absence of PP2A^Cdc55^. In contrast, both Sli15 and Ndd1 are phosphorylated later, around the time of mitotic entry. Phosphorylation of both late Cdk substrates was markedly advanced in the absence of PP2A^Cdc55^ (Figure 1B). Sli15 phosphorylation was advanced by at least 20 min, with some increased phosphorylation evident already in G1-arrested cells. Ndd1 phosphorylation was advanced by ∼10 min, a substantial difference during the short budding yeast cell cycle. These phosphorylation timing changes were reproducible between repeats of the experiment. Figure S2 shows a repeat of the Ndd1 phosphorylation time course, including additional cell-cycle markers as internal timing controls. This confirmed that Ndd1 phosphorylation is advanced in the absence of PP2A^Cdc55^.

We also probed cell extracts with antibodies raised against phosphorylated Cdk consensus peptides. This provided further evidence for both advanced as well as increased Cdk phosphorylation in cells lacking PP2A^Cdc55^ (Figure S3). Despite these examples of advanced Cdk phosphorylation, the overall kinetics of interphase progression were similar between the two strains, while mitotic exit and cytokinesis were delayed in the absence of PP2A^Cdc55^. This suggests that PP2A^Cdc55^ counteracts and thereby delays phosphorylation of some, but not all, Cdk substrates during interphase.

### PP2A^Cdc55^ Counteracts Threonine Phosphorylation

**Figure 2:**
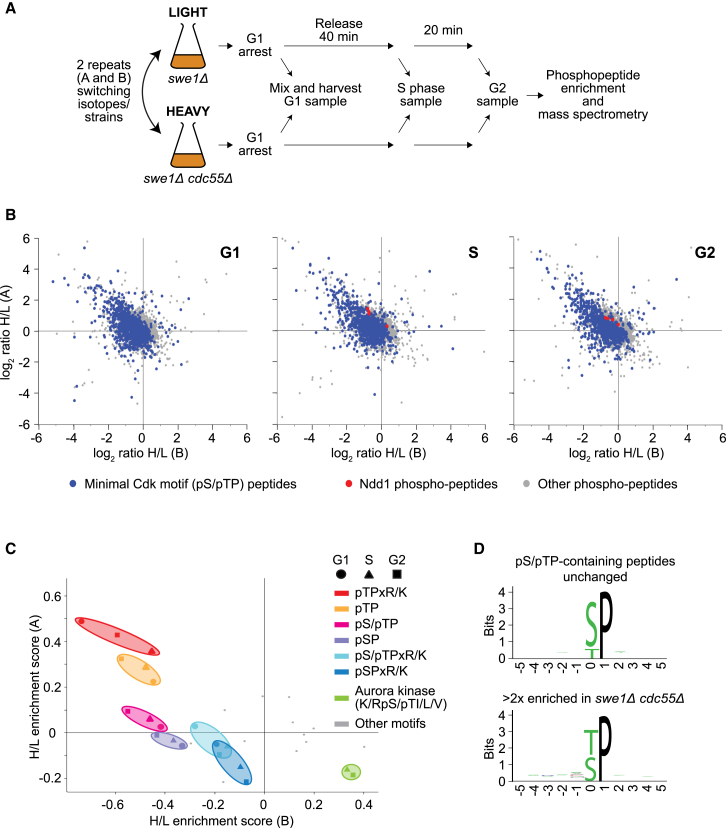
Increased Threonine-Directed Phosphorylation in Cells Lacking PP2A^Cdc55^ (A) Schematic of the experiment to compare PP2A^Cdc55^-dependent phosphoproteomes at three time points during synchronous cell-cycle progression. (B) The normalized heavy/light (H/L) ratio of all phosphosites in two repeats of the experiment, (A) and (B), is shown. 4,589 phosphosites on 1,309 proteins were quantified in both repeats, many at more than one cell-cycle stage (compare Table S1). Minimal Cdk motif-containing phosphosites are highlighted, as well as those derived from Ndd1. (C) 2D annotation enrichment analysis of phosphosite motifs whose abundance changed significantly in the absence of PP2A^Cdc55^ (p < 0.02, using an adapted Wilcoxon Mann-Whitney test as described previously; Cox and Mann, 2012). (D) Sequence logos of pSP/pTP sites enriched greater than 2-fold in the absence of PP2A^Cdc55^ or unchanged. See also Figure S4A for FACS analysis to confirm the cell-cycle state of cells at the three time points, Figure S4B for a 2D annotation of full Cdk consensus sites, Figure S4C for sequence logos taking into account the species-specific probability of amino acid occurrence, Table S1 for a summary of peptide counts, and Data S1 for the abridged mass spectrometry data.

To obtain a global view of Cdk substrates affected by PP2A^Cdc55^, we quantitatively compared the phosphoproteomes of *swe1Δ* and *swe1Δ cdc55Δ* cells at three time points during synchronous cell-cycle progression in G1, S, and G2 (Figures 2A and S4A). We used stable isotope labeling with amino acids in culture (SILAC) followed by phosphopeptide enrichment and mass spectrometry. This allowed us to compare the abundance of 4,589 phosphosites on 1,309 proteins in the presence or absence of PP2A^Cdc55^ (for details of the peptide and protein numbers identified in the three cell-cycle stages, see Table S1). Plotting the heavy/light ratio of phosphosites from two repeats with inverse isotope labeling allowed reliable identification of phosphosites enriched in the absence of PP2A^Cdc55^. These are found in the upper left quadrants of the corresponding 2D scatterplots (Figure 2B). This analysis revealed that PP2A^Cdc55^ counteracts protein phosphorylation throughout interphase in G1, S, and G2 phases. Numerous phosphosites adhering to the minimal (pS/pTP) or full (pS/pTPxR/K; Figure S4B) Cdk consensus sites were among those enriched in the absence of PP2A^Cdc55^.

As a control, to assess the variability introduced by the SILAC method, we differentially labeled two cultures of the same wild-type or mutant strains, respectively. This revealed a small degree of variation, with the vast majority of phosphosites (94.3%) detected with a less than 2-fold difference between the two labeling conditions (see the Quantification and Statistical Analysis). We therefore used a greater than 2-fold change as our selection criterion to compile phosphosites enriched in the absence of PP2A^Cdc55^, including an acceptable false discovery rate. Among those, in samples taken in S and G2 phase, we found several Ndd1-derived sites. At the same time, other Cdk phosphosites were not affected by the absence of PP2A^Cdc55^, clustering around the origin of the enrichment plots. This confirms that PP2A^Cdc55^ counteracts phosphorylation of some, but not all, Cdk substrates.

We next explored whether PP2A^Cdc55^ shows phosphorylation site preferences when selecting its substrates. To this end, we assigned each phosphosite to one or more phosphorylation site consensus motifs. We then performed a 2D annotation enrichment analysis, which tests for the enrichment of motifs at either high or low SILAC ratios, based on their rank (Cox and Mann, 2012). This revealed that Cdk phosphorylation was significantly affected by PP2A^Cdc55^. In particular, this was the case for phosphothreonine-containing, but not phosphoserine-containing, Cdk sites (Figure 2C). In a complementary approach, we generated a sequence logo of the 197 Cdk phosphosites that increased over 2-fold in the absence of PP2A^Cdc55^ and compared it to the logo of the 713 sites that increased by less or remained unchanged (Figure 2D). This again demonstrated that PP2A^Cdc55^ preferentially affects threonine phosphorylation, a result that was statistically highly significant (see the Quantification and Statistical Analysis). An in vitro preference for threonine dephosphorylation has been noted during the early biochemical characterization of PP2A using peptide substrates (Agostinis et al., 1990). Recently, a preferential impact of PP2A on threonine phosphorylation was observed in protein extracts prepared from HeLa cells (Cundell et al., 2016). Our results show that this translates into a pronounced in vivo substrate preference.

Substrate recognition by human PP2A^Cdc55^ was suggested to involve a polybasic recognition determinant (Cundell et al., 2016). Our above sequence logo of PP2A^Cdc55^-dependent phosphosites did not reveal a similar requirement (Figure 2D). When we analyzed the surrounding of PP2A^Cdc55^-regulated phosphosites using an algorithm that takes the species-specific probability of amino acid occurrence into account (Colaert et al., 2009), a slight enrichment of positively charged amino acids became detectable (Figure S4C). An acidic patch on Cdc55 that has been implicated in substrate interactions (Xu et al., 2008) could be a reason for this preference. The same argument might explain why the impact of PP2A^Cdc55^ was quantitatively greatest on full Cdk consensus sites (Figure 2C), which include an oppositely charged basic residue.

Among all other phosphorylation site motifs analyzed, the Aurora kinase motif stood out as showing significantly decreased phosphorylation in the absence of PP2A^Cdc55^ (Figure 2C). This could be a secondary consequence of increased Cdk phosphorylation on its regulatory Sli15 subunit (Figure 1B), which is thought to attenuate Aurora kinase activity (Zimniak et al., 2012).

### Threonine Phosphorylation Is a Late Cell-Cycle Event

If PP2A^Cdc55^ specifically targets phosphothreonines to set an increased Cdk threshold for their phosphorylation, threonines should be phosphorylated later in the cell cycle compared to serines. To address whether this is the case, we used SILAC followed by mass spectrometry to compare the phosphoproteomes of G2 and mitosis, using a pair of sequential samples taken from wild-type cells that synchronously passed through the cell cycle. 2D annotation enrichment analysis, incorporating a repeat experiment using inverse labeling, revealed that the phosphorylation site motif most strongly enriched in mitosis, compared to G2, was pTPxR/K (Figure S4D). This corresponds to the threonine-directed motif whose phosphorylation is most strongly counteracted by PP2A^Cdc55^. Thus, PP2A^Cdc55^-regulated phosphorylation events indeed correspond to those that are reserved until late in the cell cycle.

**Figure 3:**
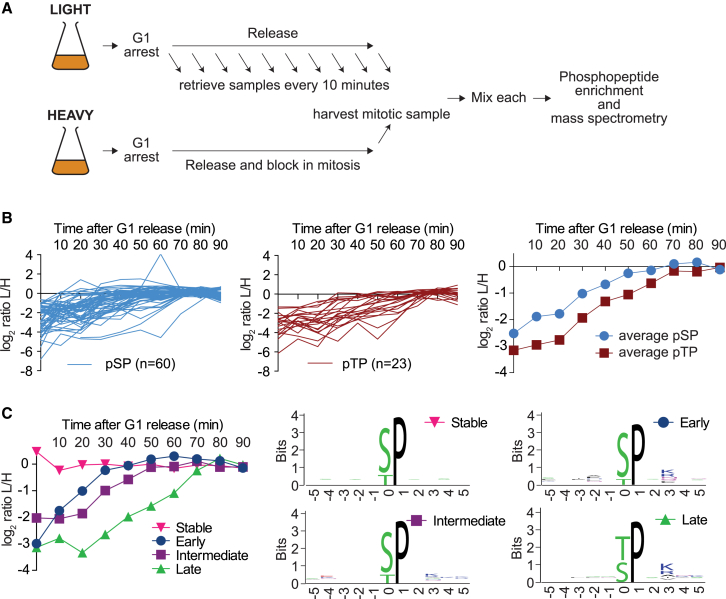
Threonine-Directed Phosphorylation Occurs Late in the Cell Cycle (A) Schematic of the experiment to analyze time-resolved phosphoproteome changes during synchronous cell-cycle progression. See also Figure S4D for an experiment comparing phosphoproteome changes between G2 and M. (B) The intensity profiles of all increasing pSP and pTP sites are plotted over the course of cell-cycle progression and normalized to the intensity at 90 min (left and middle). The averages of all profiles are compared (right). (C) The average intensity profiles of all pSP/pTP sites, divided into sequential early, intermediate, and late categories (reaching their maximal levels at 10–30, 40–60, and 70–90 min, respectively) as well as stable phosphosites, are plotted together with the sequence logos surrounding the phosphorylation site in each category. See also Figures S5A and S5B for cell-cycle analysis and for the aggregate traces of all the sites, subdivided into phosphorylation timing categories, Table S1 for a summary of peptide counts, and Data S2 for the abridged mass spectrometry data.

To gain a time-resolved picture of serine and threonine phosphorylation, we performed a mass-spectrometry-based time course experiment. Samples were taken at 10-min intervals from a synchronous culture grown in light amino acids that progressed from G1 to anaphase. Each sample was combined with an aliquot of a metaphase sample grown in heavy amino acids (Figure 3A). This allowed us to follow the abundance of 405 phosphoserine-proline (pSP)- or phosphothreonine-proline (pTP)-containing phosphosites during cell-cycle progression. 83 (20%) of these showed a greater than 2-fold increase between G1 and mitosis, the rest were either stable or displayed unclear patterns (Figure S5). Categorizing the increasing phosphosites into pSPs and pTPs and plotting their abundance over time made apparent that pSP sites indeed increased earlier in the cell cycle compared to pTP sites (Figure 3B). This became additionally evident from plotting the average phosphorylation kinetics of all the increasing phosphosites within these two categories. pTP sites started out in G1 at a lower relative level of phosphorylation and reached their peak of phosphorylation 10–20 min after pSP sites.

In a complementary analysis, we subdivided all Cdk sites into early, intermediate, late, or stable categories depending on the time they reached their maximal level of phosphorylation. Early sites included known early Cdk targets (e.g., Whi5 and Stb1), while late sites included known late substrates (e.g., Ndd1 and Net1), thus validating our assignments. The averaged phosphorylation kinetics of each group over time is shown in Figure 3C (aggregate traces of all phosphosites can be found in Figure S5B). We then created sequence logos of the phosphosites in each category. This revealed that only 3 out of 25 early phosphorylated sites were pTP, which increased over 4-fold to 14 out of 26 late phosphosites (Table S1). A χ^2^ test across the time categories confirmed that late phosphorylated sites are significantly enriched for pTP compared to the other categories (p = 0.0013, corrected for multiple testing). Together, these results reveal that phosphothreonines are preferentially kept underphosphorylated by PP2A^Cdc55^ and that this correlates with their late phosphorylation during the cell cycle.

### Threonine Identity Confers Late Ndd1 Phosphorylation

**Figure 4:**
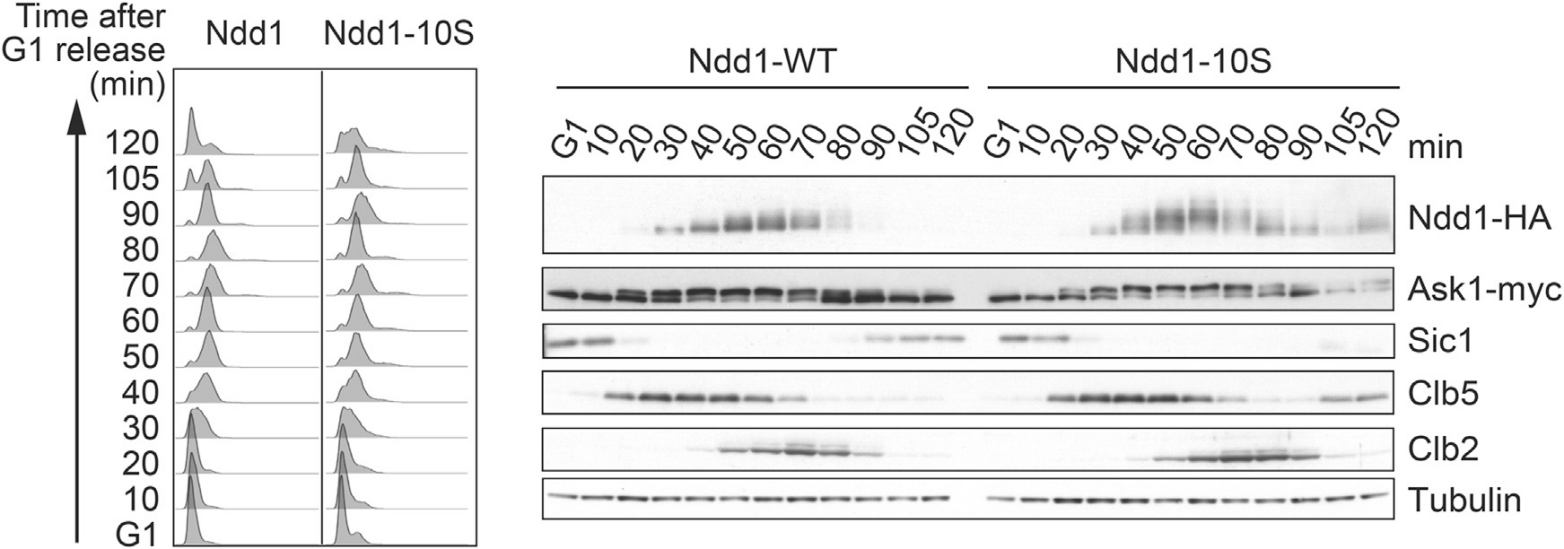
Threonine Identity of the Cdk Acceptor Sites Defines Late Ndd1 Phosphorylation Wild-type strains, carrying wild-type Ndd1 or Ndd1-10S, progressed synchronously through the cell cycle following α-factor arrest and release. The Ndd1 phosphorylation status was analyzed by western blotting. Ask1 phosphorylation and the levels of Sic1, Clb5, and Clb2 served as internal timing controls. Tubulin was the loading control. See also Figure S5C, which shows that PP2A^Cdc55^ retains only a small effect on the phosphorylation timing of Ndd1-10S.

We next wanted to establish a causal relationship between the nature of the phosphoacceptor amino acid and its phosphorylation timing. Therefore, we asked whether threonine identity not only correlates with but also is responsible for late Cdk substrate phosphorylation. To do this, we replaced ten threonine Cdk target sites in Ndd1 (Reynolds et al., 2003) with serines. In an otherwise wild-type strain background, phosphorylation of the resulting Ndd1-10S protein was both advanced and increased during cell-cycle progression (Figure 4). Ask1 phosphorylation, which remained unaffected, served as an internal timing comparison. This confirms that the threonine nature of the Cdk target sites on Ndd1 is instrumental in restricting their phosphorylation to mitosis. Consistent with the idea that PP2A^Cdc55^ delays Ndd1 phosphorylation by targeting threonines, PP2A^Cdc55^ impacted on the phosphorylation timing of Ndd1-10S to a smaller degree when compared to wild-type Ndd1 (Figure S5C).

### Accelerated Cell-Cycle Progression in the Absence of PP2A^Cdc55^

**Figure 5:**
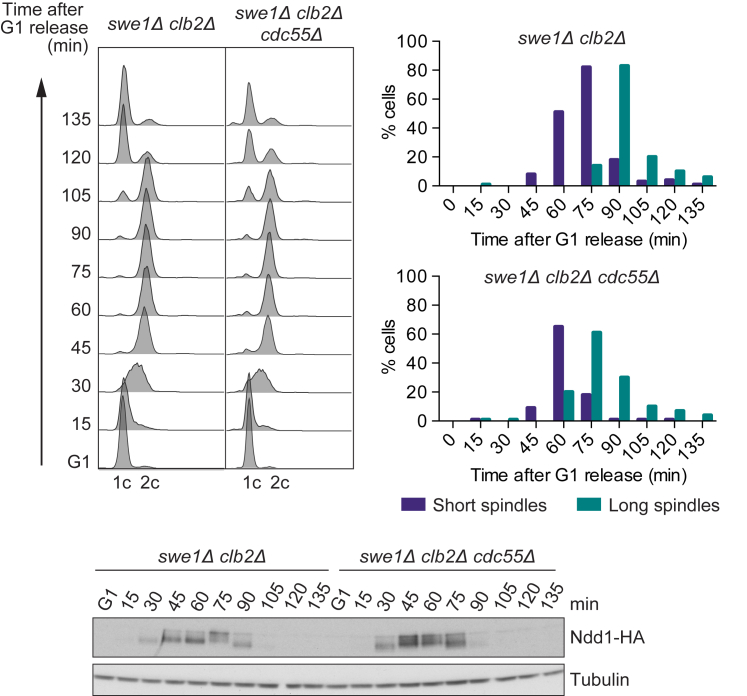
Accelerated Cell-Cycle Progression in the Absence of PP2A^Cdc55^ Cell-cycle progression in cells lacking Swe1 and Clb2 was compared to cells that additionally lacked PP2A^Cdc55^. Cells were arrested in G1 by pheromone α-factor treatment and released to progress through a synchronous cell cycle before re-arrest in the next G1 phase by α-factor re-addition. Cell-cycle progression was monitored by FACS analysis of DNA content. The percentage of cells showing short (<2 μm) or long (≥2 μm) spindles is plotted over time. Ndd1 phosphorylation kinetics were analyzed by western blotting. Tubulin served as a loading control. See also Figure S6, which shows that APC and Net1 phosphorylation can only partly explain the impact of PP2A^Cdc55^ on mitotic timing.

If delayed threonine phosphorylation contributes to the ordering of cell-cycle progression, one should expect accelerated mitotic entry in the absence of PP2A^Cdc55^, when these late phosphorylation events are advanced. However, the relative timing of S phase and mitosis was similar in *swe1Δ* and *swe1Δ cdc55Δ* cells (Figure 1B). This could be because PP2A^Cdc55^ acts redundantly with another phosphatase that targets a complementary set of Cdk substrates or redundantly with another ordering principle. Alternatively, the timing of mitosis, which is advanced in the *swe1Δ* background, cannot be further advanced due to intrinsic time requirements of the underlying cell biological events (Georgi et al., 2002). To differentiate between these scenarios, we aimed to compensate for increased Cdk activity in *swe1Δ* cells by removing Clb2, one of the four mitotic cyclins (Fitch et al., 1992). This reversed the accelerated mitosis seen in *swe1Δ* cells (Figure S6A). In synchronized cultures, we monitored DNA content as a sign of S phase, spindle formation as a marker for G2, and the beginning of spindle elongation as indicator for mitosis when the anaphase-promoting complex (APC) is activated as Cdk activity reaches its peak (Figure 5). *cdc55* deletion in the *swe1Δ clb2Δ* background did not change the timing of S and G2 phases but caused a notable advance of anaphase onset. Ndd1 phosphorylation was also shifted to earlier time points. These observations suggest that PP2A^Cdc55^ indeed delays the onset of mitosis.

It has been suggested that PP2A^Cdc55^ counteracts APC phosphorylation as part of a Swe1-dependent morphogenesis checkpoint (Lianga et al., 2013). We therefore tested whether APC is also a PP2A^Cdc55^ target that defines mitotic timing. We utilized the *apc-12A* strain (Lianga et al., 2013), in which 12 activatory phosphorylation sites on three APC subunits (including five threonines) are replaced by alanine, thus mimicking a constitutively dephosphorylated state. The consequent mitotic delay in *swe1Δ apc-12A* cells (even more pronounced in *swe1Δ apc-12A clb2Δ* cells) was substantially reduced by *cdc55* deletion (Figure S6A), suggesting that PP2A^Cdc55^ targets substrates in addition to the APC. Another previously characterized PP2A^Cdc55^ target is the Cdc14 phosphatase inhibitor Net1 (Queralt et al., 2006). To test whether premature Net1 phosphorylation and consequent Cdc14 activation advances the cell cycle, we utilized the *CDC14*^*TAB6*^ allele (Shou and Deshaies, 2002), which similarly advances Cdc14 activation. *CDC14*^*TAB6*^ only slightly advanced mitosis in *clb2Δ* cells, while *cdc55* deletion in this background further accelerated mitotic progression (Figure S6B). Together, this suggests that APC and Net1 are two of potentially many PP2A^Cdc55^ targets that together define mitotic timing.

### PP2A^Cdc55^ Causes a Dosage-Dependent Cell-Cycle Delay

**Figure 6:**
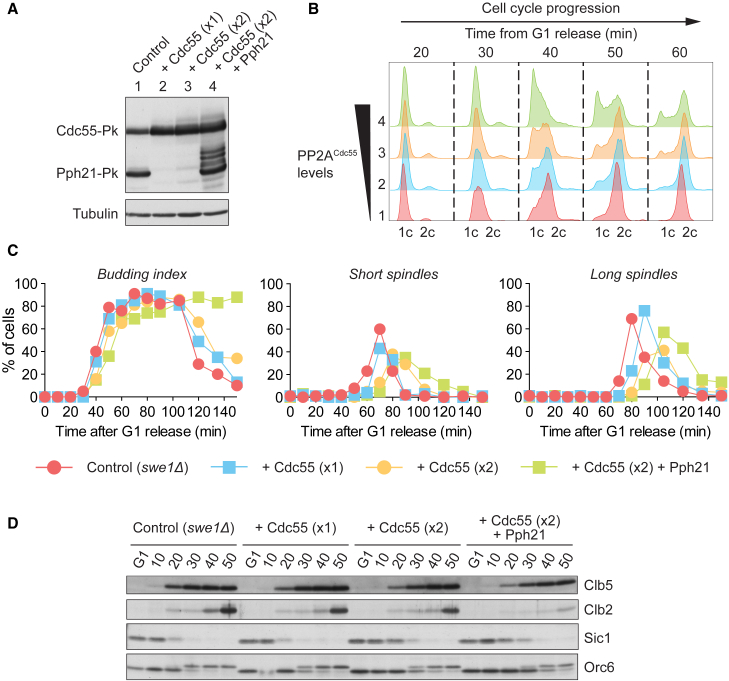
Ectopic PP2A^Cdc55^ Delays Cell-Cycle Progression in a Dosage-Dependent Manner (A) Increased Cdc55 and Pph21 levels following overexpression of these PP2A^Cdc55^ subunits were analyzed by western blotting from cells growing asynchronously in galactose-containing medium. (B) Cells shown in (A) were arrested in G1 by α-factor treatment and released to progress through a synchronous cell cycle before re-arrest in the next G1 phase by α-factor re-addition. FACS analysis of DNA content between 20 and 60 min after release is shown to compare S phase progression. (C) The budding index and fraction of cells with short (<2 μm) or long (≥2 μm) spindles were scored in cell aliquots from the experiment in (B). (D) Protein extracts were prepared from aliquots of the same cultures, and the abundance and phosphorylation status of the indicated proteins was analyzed by western blotting.

Additional evidence that PP2A^Cdc55^ is a rate-limiting factor that counteracts Cdk phosphorylation during cell-cycle progression came from an experiment using increased PP2A^Cdc55^ levels. Ectopic Cdc55 expression, and co-expression of the PP2A catalytic subunit Pph21, led to a dosage-dependent slowdown of cell-cycle progression (Figures 6A–6C). A delay was apparent both during S phase (DNA replication and bud formation) and more pronounced during mitosis (spindle formation and anaphase onset). Monitoring Cdk-dependent cell-cycle events by western blotting confirmed these observations. Early events, such as Sic1 degradation and Clb5 accumulation, were delayed to a lesser degree than later events, such as Orc6 phosphorylation and Clb2 accumulation (Figure 6D). This suggests that PP2A^Cdc55^ counteracts phosphorylation of substrates involved at multiple stages of the cell cycle. The fact that later events were more strongly affected than earlier events is consistent with the idea that late phosphorylated substrates are more sensitive toward the phosphatase.

## Discussion

Our results show that PP2A^Cdc55^ counteracts global Cdk phosphorylation during the budding yeast cell cycle in a manner that is independent of the phosphatase’s additional role in regulating Cdk tyrosine phosphorylation. We consider it likely that PP2A^Cdc55^ directly dephosphorylates Cdk substrates and thereby delays their phosphorylation until Cdk activity has risen above a threshold required to shift the phosphorylation equilibrium to the phosphorylated state. A reason to believe that PP2A^Cdc55^ acts at the Cdk substrate level is its threonine selectivity, consistent with the observed in vitro PP2A^Cdc55^ specificity (Agostinis et al., 1990, Cundell et al., 2016). Threonine selectivity goes some way to answer the question how PP2A^Cdc55^ selects its substrates. Additional substrate features are likely to play a role in defining PP2A^Cdc55^ targets. In particular, it will be interesting to investigate how PP2A^Cdc55^ dephosphorylation efficiency is quantitatively defined for each substrate, which we expect will equate to the Cdk threshold that must be surpassed to achieve net phosphorylation. Similar to reduced phosphatase activity, increased kinase activity should accelerate mitotic entry, consistent with what has been observed (Oikonomou and Cross, 2011). A preference of Cdk for serine over threonine, which has been observed in case of vertebrate cyclin B1-Cdk1 (Suzuki et al., 2015), might in addition contribute to early serine over threonine phosphorylation.

Threonines account for only one-fifth of the cell-cycle-regulated phosphorylation events that we detected in our phosphoproteome analysis. Clearly, ordered phosphorylation extends beyond threonines to the numerous serine-bearing substrates. One possible explanation for ordered serine phosphorylation is that another phosphatase sets substrate-specific thresholds for their phosphorylation. Budding yeast Cdc14 displays serine preference (Bremmer et al., 2012); however, it is thought to be inactive during interphase. Accordingly, our preliminary analysis did not reveal a notable impact of Cdc14 on interphase phosphorylation (data not shown). A yet-to-be-assigned phosphatase might set thresholds for serine phosphorylation during interphase.

Ordering of cell-cycle phosphorylation by Cdk-counteracting phosphatases implies that individual substrates undergo repeated cycles of phosphorylation and dephosphorylation. A similar, if reciprocal, mechanism has been suggested to order substrate dephosphorylation during mitotic exit (Bouchoux and Uhlmann, 2011). While such phosphorylation cycles can be seen as “futile,” they allow the cell-cycle control machinery to impose thresholds and timings. The resultant phosphorylation equilibria allow phosphorylation states to remain stable, for example, when cell-cycle progression is halted due to replication or DNA damage checkpoint activation. Depending on the turnover rate of kinase and phosphatase (Bouchoux and Uhlmann, 2011, Holt et al., 2009, Liakopoulos et al., 2003), tens to hundreds of ATPs might be spent to control a phosphorylation site over the duration of one cell cycle. While sizeable, this expenditure is small compared to the total ATP requirement for synthesizing a protein.

The finding that the nature of the phosphoacceptor amino acid affects its phosphorylation timing opens up an unanticipated level of regulatory potential, not only in cell-cycle control but also other processes that are controlled by phosphorylation. Phosphoacceptor amino acid specificity has been recorded in the cases of several phosphatases, including Cdc14, PP1, and PP2A (Agostinis et al., 1990, Agostinis et al., 1992, Bremmer et al., 2012). This opens the possibility that other biological processes that are affected by these phosphatases contain a regulatory element dependent on the phosphoacceptor amino acid.

## STAR★Methods

### Key Resources Table

**Table:** 

REAGENT or RESOURCE	SOURCE	IDENTIFIER
**Antibodies**		

α-Clb5	Santa Cruz	sc20170; RRID: AB_671845
α-Clb2	Santa Cruz	sc9071; RRID: AB_667962
α-Sic1	Santa Cruz	sc50441; RRID: AB_785671
α-Orc6	in house	clone SB49
α-Tub1 (α-tubulin)	Bio-Rad	YOL1/34
α-myc	in house	clone 9E10
α-HA	in house	clone 12CA5
α-Pk	Bio-Rad	SV5-Pk1
α-phosphoMAPK/CDK Substrates	Cell Signaling	#2325; RRID: AB_331820
α-phosphoCDK Substrate Motif	Cell Signaling	#9477
α-phosphothreonine-proline	Cell Signaling	#9391; RRID: AB_331801
Alexa Fluor 594 coupled goat α-rat secondary antibody	Molecular Probes	A11007; RRID: AB_141374

**Chemicals, Peptides, and Recombinant Proteins**		

Trypsin Gold, Mass Spectrometry Grade	Promega	V5280
rLys-C, Mass Spec Grade	Promega	V1671
Sep-Pak C18 Plus Light Cartridge, 130 mg	Waters	WAT023501
Titansphere TiO, 5 μm	GL Sciences	5020-75000

**Critical Commercial Assays**		

Liquid Chromatography and Columns		
Dionex UltiMate 3000 HPLC	Thermo Scientific	5041.0010
EASY-Spray C18 column, 75 μm × 50 cm	Thermo Scientific	ES803
Mass spectrometry		
LTQ-Orbitrap Velos	Thermo Scientific	1239200

**Deposited Data**		

Raw mass spectrometry proteomics data	ProteomeXchange Consortium via the PRIDE partner repository	ProteomeXchange: PXD004461
MaxQuant output mass spectrometry proteomics data files	ProteomeXchange Consortium via the PRIDE partner repository	ProteomeXchange: PXD004461

**Experimental Models: Organisms/Strains**		

All *Saccharomyces cerevisiae* yeast strains used in this study were of the W303 or S288C background and are listed in Table S2.		N/A

**Software and Algorithms**		

MaxQuant (version 1.3.0.5)	Open Source	http://www.biochem.mpg.de/5111795/maxquant
Perseus (version 1.4.0.11)	Open Source	http://www.biochem.mpg.de/5111810/perseus
WebLogo	Open Source	weblogo.berkeley.edu/logo.cgi
iceLogo	Open Source	http://iomics.ugent.be/icelogoserver/index.html

### Contact for Reagents

Further information and requests for reagents may be directed to, and will be fulfilled by, the corresponding author, Frank Uhlmann (frank.uhlmann@crick.ac.uk).

### Method Details

#### Yeast Strains and Techniques

Epitope tagging of endogenous genes and gene deletions were performed by gene targeting using polymerase chain reaction (PCR) products (Knop et al., 1999, Wach et al., 1994). Cdc55 overexpression was achieved by cloning the *CDC55* gene under *GAL1* promoter control into YIplac128 (Gietz and Sugino, 1988), including a Pk epitope tag for detection at the C terminus. The resulting plasmid was integrated into the budding yeast genome at the *LEU2* locus. Colonies were screened for various expression levels, following galactose induction, that are the consequence of different multiplicities of integration. Pph21 co-overexpression was similarly achieved by cloning *PPH21* under *GAL1* promoter control into a vector that also harbored the *GAL4* gene, expressed from the same bidirectional promoter in the *GAL10* direction. Increased Gal4 transcription factor levels facilitate expression of more than one protein under galactose control. The *NDD1-10S* allele was based on a synthetic DNA construct (GeneArt, Life Technologies), that was integrated to replace endogenous *NDD1*. Yeast cultures were grown in rich YP medium supplemented with 2% glucose or with 2% raffinose + 2% galactose (Rose et al., 1990), if not stated otherwise. Cell synchronization using α-factor block and release was performed as described (O’Reilly et al., 2012).

#### Western Blotting

Protein extracts for western blotting were prepared following cell fixation using trichloroacetic acid, as described by (Foiani et al., 1994), and analyzed by SDS-polyacrylamide gel electrophoresis. Antibodies used for detection are listed in the Key Resources Table.

#### Immunofluorescence Microscopy

Indirect immunofluorescence was performed on formaldehyde-fixed cells using the α-Tub1 antibody and Alexa Fluor 594-coupled secondary antibody listed in the Key Resources Table. Cells were counterstained with the DNA binding dye 4’,6-diamidino-2-phenylindole (DAPI). Fluorescent images were acquired using an Axioplan 2 imaging microscope (Zeiss) equipped with a 100x (NA = 1.45) Plan-Neofluar objective and an ORCA-ER camera (Hamamatsu). Spindles < 2 μm in length were counted as short (G2/M) spindles, spindles that were 2 μm or longer were classified as long (anaphase) spindles.

#### Stable Isotope Labeling with Amino Acids In Cell Culture (SILAC) and Mass Spectrometry

Cell cultures were grown for > 8 generations in synthetic complete medium containing 0.1 mg/ml of arginine and lysine or [^13^C_6_]arginine and [^13^C_6_]lysine, respectively (Gruhler et al., 2005, Rose et al., 1990), then synchronized and released. At the time points described in the individual experiments, aliquots of the cultures were retrieved and mixed. Cells were harvested by centrifugation and resuspended in 20% trichloroacetic acid for protein fixation. Following acetone washes, cells were resuspended in lysis buffer (50 mM ammonium bicarbonate, 5 mM EDTA pH 7.5, 8 M urea) and opened by glass bead breakage. The protein extract was cleared by centrifugation.

##### Sample Preparation for Mass Spectrometry

1 mg of protein sample was reduced by 5 mM dithiothreitol (DTT) (56°C, 25 min), alkylated with 10 mM iodoacetamide (room temperature, 30 min, dark) and quenched with 7.5 mM DTT. Samples were then diluted with 50 mM ammonium bicarbonate to reduce the urea concentration to < 2 M, prior to trypsin digestion (37°C, overnight). Peptides were then desalted using a C_18_ SepPak Lite (130 mg bed volume) under vacuum and dried. To ensure complete digestion, peptides were further digested using Lys-C in 10% acetonitrile, 50 mM ammonium bicarbonate (37°C, 2 h), followed by trypsin digestion (37°C, overnight). Digested proteins were then desalted again and dried.

Phosphopeptide enrichment using titanium dioxide (TiO_2_) was carried out as follows. Dried peptide mixtures were re-suspended in 1 M glycolic acid + 80% acetonitrile + 5% trifluoroacetic acid, sonicated (10 min) and added to titanium dioxide beads (5:1 (w/w) beads:protein). The beads were washed using 80% acetonitrile + 1% trifluoroacetic acid, followed by 10% acetonitrile + 0.2% trifluoroacetic acid, and dried under vacuum centrifugation. Phosphopeptides were eluted from the beads by adding 1% ammonium hydroxide followed by 5% ammonium hydroxide, and dried by vacuum centrifugation. Dried phosphopeptides were re-suspended in 100 μL of 1% trifluoroacetic acid and sonicated (15 min). A C_18_ membrane was packed into a 200 μL pipette tip and washed using methanol and equilibrated with 1% trifluoroacetic acid. The peptides were loaded onto the Stage Tip and washed with 1% trifluoroacetic acid followed by elution with 80% acetonitrile + 5% trifluoroacetic acid. The eluted peptides were again dried by vacuum centrifugation.

An LTQ-Orbitrap Velos was used for data acquisition. Phosphopeptide mixtures were re-suspended in 35 μL 0.1% trifluoroacetic acid and injected three times (10 μL per injection). Each run consisted of a 3 hr gradient elution (75 μm × 50 cm C_18_ column) with one activation method per run: collision induced dissociation (CID), multi-stage activation (MSA) and higher energy collision dissociation (HCD).

##### Processing and Analysis of Mass Spectrometry Data

MaxQuant (version 1.3.0.5) was used for all data processing. The data were searched against a UniProt extracted *S. cerevisiae* proteome FASTA file amended to include common contaminants. A decoy database containing reverse sequences was used to estimate false discovery rates and set the false discovery rate at 1%. Default MaxQuant parameters were used with the following adjustments: Phospho(STY) was added as a variable modification, ‘Filter labeled amino acids’ was deselected, re-quantify was selected with the instruction to keep low-scoring versions of identified peptides within parameter groups and match between runs was selected. MaxQuant output files were imported into Perseus (version 1.4.0.2) and the normalized heavy-to-light (H:L) ratios were used for all subsequent analyses. Data were also imported into R for statistical testing and data visualization.

### Quantification and Statistical Analysis

#### 2D Annotation Enrichment Analysis, Sequence Logos, and the “Lateness” of Threonine Phosphorylation

2D annotation enrichment analysis (Cox and Mann, 2012) was performed in Perseus, using p < 0.02 in the adapted Wilcoxon Mann-Whitney test as cutoff for flagging up enrichment. Sequence logos were created using WebLogo (Crooks et al., 2004) and iceLogo (Colaert et al., 2009). In the time-course experiment to follow protein phosphorylation during progression from G1 into mitosis, peptides were included in the analysis if they were identified in at least 8 out of the 10 time points. The conclusion that threonine phosphorylation is a late event in the cell cycle was tested using a χ^2^-test across the time categories presented in Figure 3, with ‘late’ compared to the other categories and corrected for multiple testing.

#### SILAC Experimental Design and Evaluation whether PP2A^Cdc55^ Preferentially Affects Threonines

For the experiment in Figure 2, comparing phosphosite abundance in the presence and absence of PP2A^Cdc55^, *S. cerevisiae* strains were grown in ‘heavy’ or ‘light’ SILAC medium and mixtures were prepared according an experimental design table, shown as Table S3. Each cell in the table represents a possible SILAC mixture, with its composition shown in the table margin. The six experimental mixtures are highlighted in green, whereas the six control mixtures are highlighted in yellow. The experimental mixtures in which the *cdc55Δ* strain was grown in ‘heavy’ and the wild-type in ‘light’ medium were considered the forward (F) conditions. The mixtures in which the *cdc55Δ* strain was grown in ‘light’ and the wild-type in ‘heavy’ medium were considered the reverse (R) conditions. The same strain was used in the ‘heavy’ and ‘light’ conditions for each of the control mixtures that are shown in the table diagonal. The expected heavy/light (H/L) ratios for phosphorylation sites in the control mixtures are 1.0 (0.0 in a log_2_ scale) and the ratio variability is a measure of the reproducibility of the experiment. The total number of phosphorylation sites identified (1% FDR) in the 12 SILAC experiments was 5949. The total number of phosphorylation sites quantified for each individual mixture is shown in each cell of Table S3, as well as the number of quantified sites that contained a phosphorylated serine or threonine within a full Cdk consensus motif (S/TPxK/R).

To assess the reproducibility of the experiments, the SILAC ratios of each phosphorylation site in the control experiments were plotted against the intensities in a log_2_ ratio versus log_10_ intensity plot. For each of the control experiments, over 90% of the data points were contained within a two-fold change interval (Figure S7).

Next, the phosphoproteomes of the *cdc55Δ* and wild-type strains were compared in cells synchronized in G1, S and G2. Ratio versus intensity plots consistently displayed an enrichment of Cdk consensus motif phosphorylation on threonine-containing sites but not, or only to a lesser extent, on Cdk consensus motifs containing serines. To test the significance of increased Cdk threonine site phosphorylation, we generated cumulative frequency graphs in which all sites are ordered as a function of their change between the ‘heavy’ and ‘light’ samples. This confirmed the enrichment of Cdk consensus motif phosphorylation on threonines (Figure S8). As expected, this effect was opposite in forward and reverse experiments whereas little to no effect was observed in the control mixtures. The p values of Wilcoxon-Mann-Whitney tests, indicating whether the category is enriched at high or low values, revealed highly significant enrichment of Cdk threonine site phosphorylation in the absence of PP2A^Cdc55^ (Figure S8).

### Data and Software Availability

Abridged tables of the mass spectrometry data are contained in Data S1, S2, and S3. The accession number for the full mass spectrometry proteomics data reported in this paper is ProteomeXchange: PXD004461 (Vizcaíno et al., 2016; http://www.ebi.ac.uk/prode/archive/). Raw data, all MaxQuant output files, and MaxQuant output Phospho(STY) have been deposited.

## Author Contributions

M.G. and F.U. conceived the study, M.G. performed most experiments, S.A.T. and M.K. contributed the Ndd1 phosphorylation acceptor site analysis and performed additional experiments during the revisions, A.J. and A.P.S. performed the phosphoproteome analyses, and M.G. and F.U. wrote the manuscript with input from all authors.

## Supplemental Information

Document S1. Figures S1–S8 and Tables S1–S3

Data S1. Abridged Mass Spectrometry Data Comparing Phosphosite Abundance between a Wild-Type and *cdc55Δ* Strain at Three Cell-Cycle Stages, G1, S and G2, Related to Figure 2

Data S2. Abridged Mass Spectrometry Data of the Experiment to Follow Phosphosite Abundance during Synchronous Cell-Cycle Progression of a Wild-Type Strain, Related to Figure 3

Data S3. Abridged Mass Spectrometry Data Comparing the Phosphosite Abundance in a Wild-Type Strain between G2 and M, Related to Figure S4D

Document S2. Article plus Supplemental Information
