# Supplementary material for: PP2ACdc55 Phosphatase Imposes Ordered Cell-Cycle Phosphorylation by Opposing Threonine Phosphorylation
Source: Mol Cell. 2017 Feb 2;65(3):393–402.e3. doi: 10.1016/j.molcel.2016.12.018 (PMC5296252; doi:10.1016/j.molcel.2016.12.018)
Supplement: Document S1. Figures S1–S8 and Tables S1–S3 [file mmc1.pdf]

Molecular Cell, Volume *65*

Supplemental Information

PP2A^{Cdc55} Phosphatase Imposes

Ordered Cell-Cycle Phosphorylation

by Opposing Threonine Phosphorylation

Molly Godfrey, Sandra A. Touati, Meghna Kataria, Andrew Jones, Ambrosius P. Snijders, and Frank Uhlmann

Figure S1

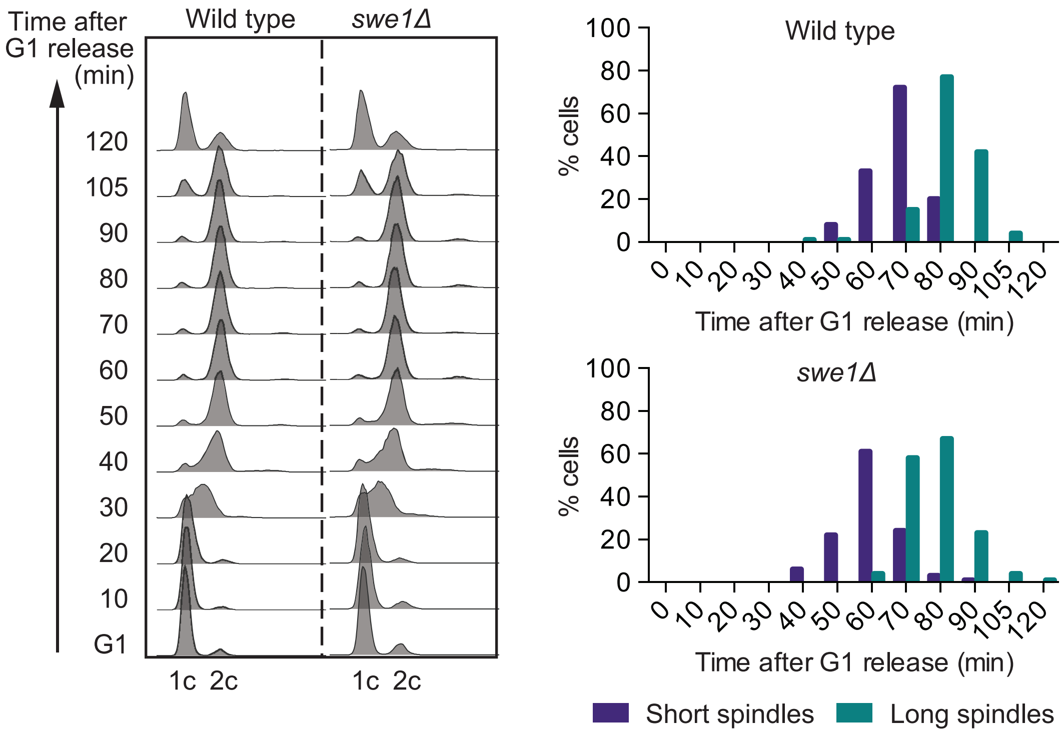

Time after G1 release (min)
Wild type
swe1Δ
120
105
90
80
70
60
50
40
30
20
10
G1
1c 2c
1c 2c
Wild type
% cells
100
80
60
40
20
0
0 10 20 30 40 50 60 70 80 90 105 120
Time after G1 release (min)
swe1Δ
% cells
Time after G1 release (min)
Short spindles
Long spindles

Figure S2

A

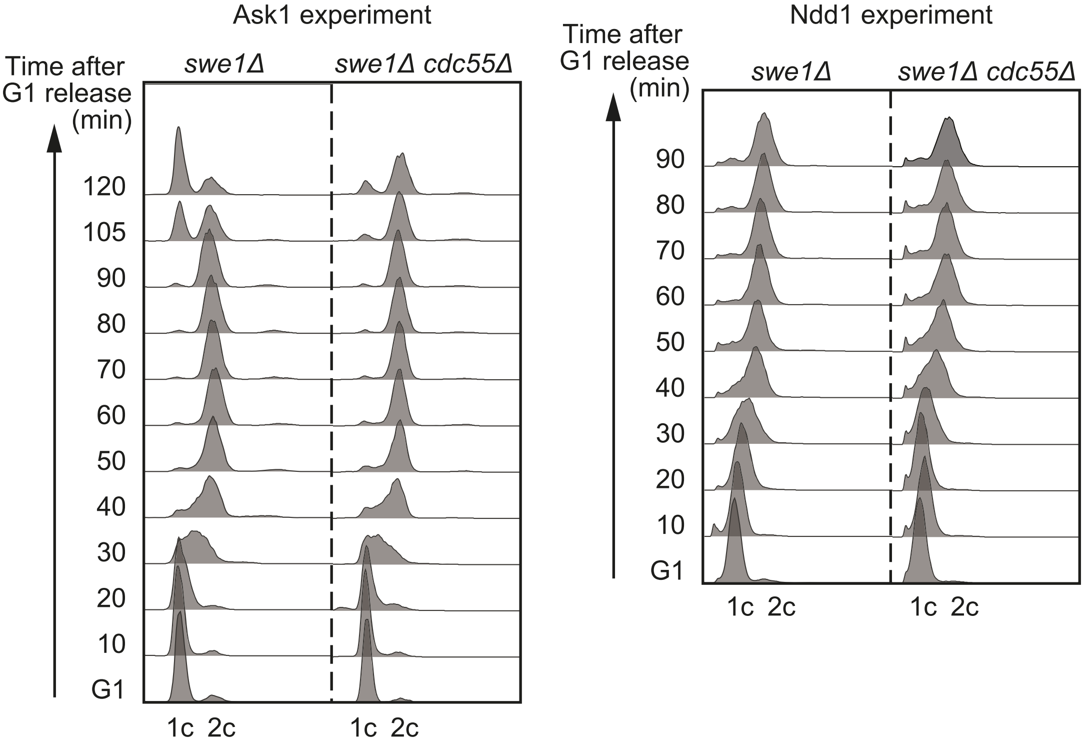
Ask1 experiment
Time after G1 release (min)
swe1Δ
swe1Δ cdc55Δ
120
105
90
80
70
60
50
40
30
20
10
G1
1c 2c
1c 2c
Ndd1 experiment
Time after G1 release (min)
swe1Δ
swe1Δ cdc55Δ
90
80
70
60
50
40
30
20
10
G1
1c 2c
1c 2c

B

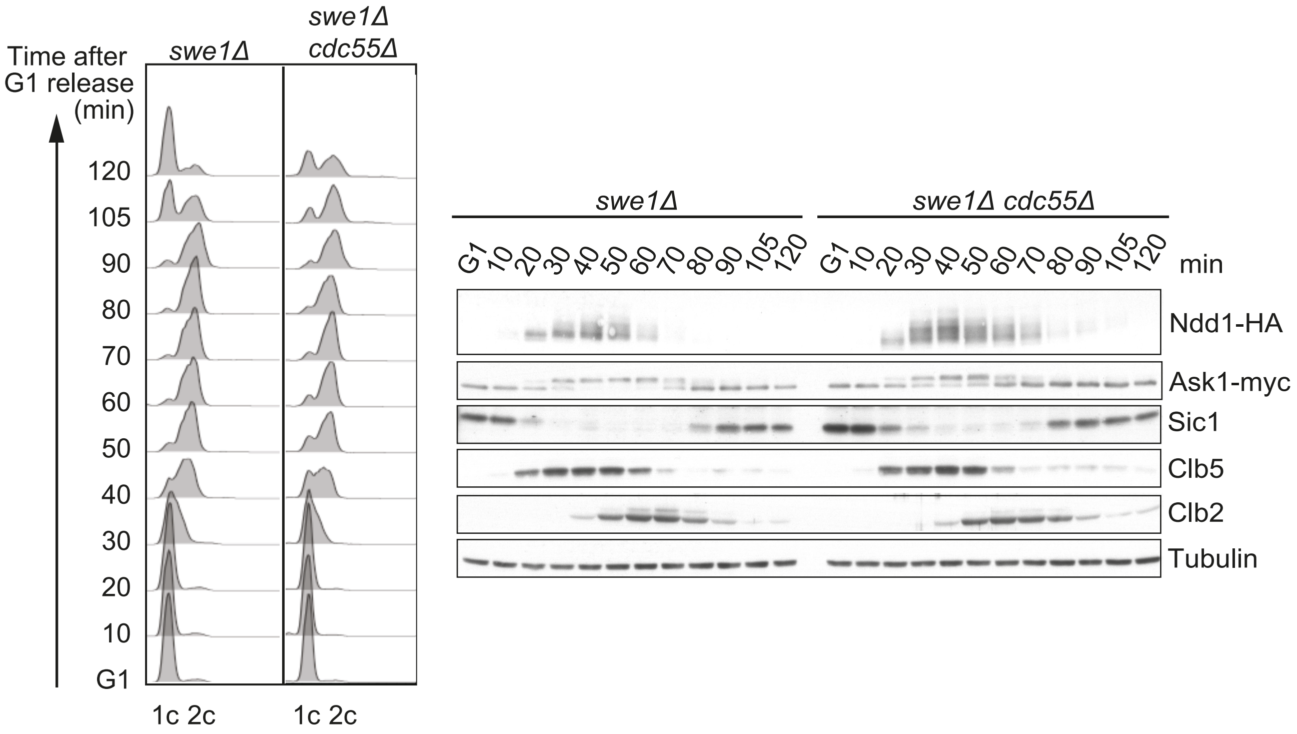
Time after G1 release (min)
swe1Δ
swe1Δ cdc55Δ
120
105
90
80
70
60
50
40
30
20
10
G1
1c 2c
1c 2c
swe1Δ
swe1Δ cdc55Δ
G1 10 20 30 40 50 60 70 80 90 105 120
G1 10 20 30 40 50 60 70 80 90 105 120
min
Ndd1-HA
Ask1-myc
Sic1
Clb5
Clb2
Tubulin

Figure S3

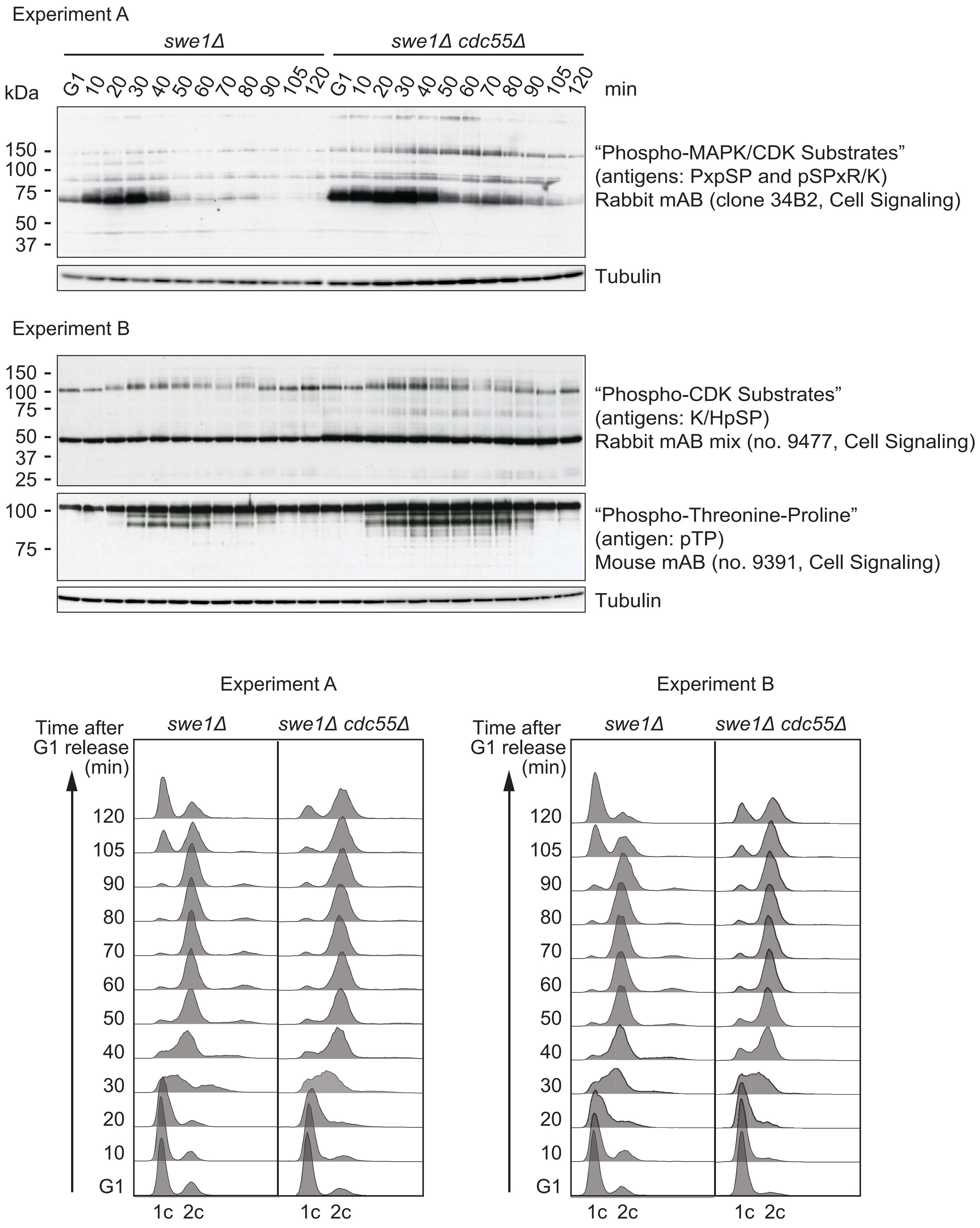
Experiment A
swe1Δ
swe1Δ cdc55Δ
G1 10 20 30 40 50 60 70 80 90 105 120 G1 10 20 30 40 50 60 70 80 90 105 120 min
kDa
150
100
75
50
37
“Phospho-MAPK/CDK Substrates”
(antigens: PxpSP and pSPxR/K)
Rabbit mAB (clone 34B2, Cell Signaling)
Tubulin
Experiment B
150
100
75
50
37
25
“Phospho-CDK Substrates”
(antigens: K/HpSP)
Rabbit mAB mix (no. 9477, Cell Signaling)
100
75
“Phospho-Threonine-Proline”
(antigen: pTP)
Mouse mAB (no. 9391, Cell Signaling)
Tubulin
Experiment A
Experiment B
Time after G1 release (min)
swe1Δ
swe1Δ cdc55Δ
120
105
90
80
70
60
50
40
30
20
10
G1
1c 2c
1c 2c

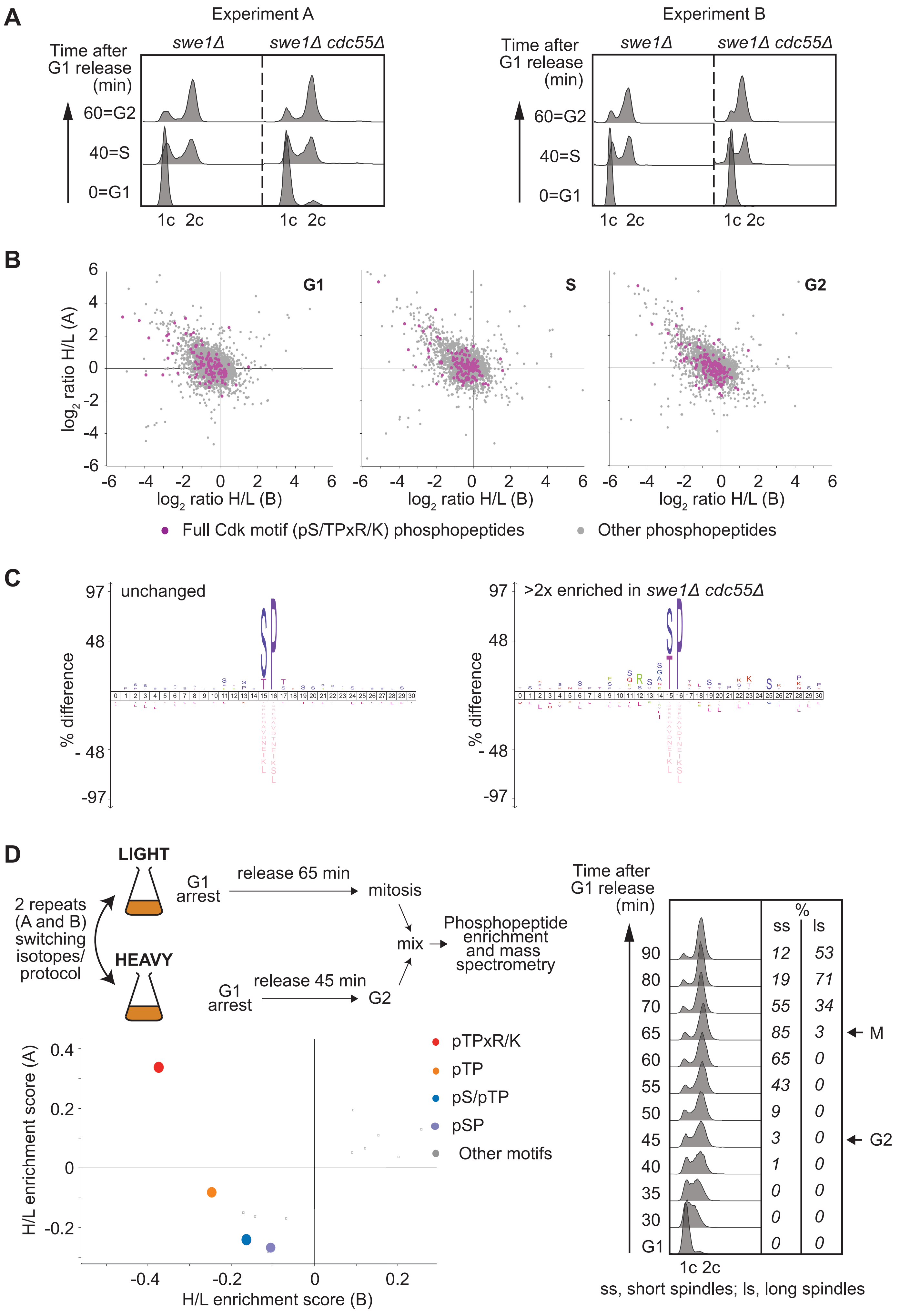
A
Experiment A
swe1Δ
swe1Δ cdc55Δ
Time after G1 release (min)
60=G2
40=S
0=G1
1c 2c
Experiment B
B
G1
S
G2
log2 ratio H/L (A)
log2 ratio H/L (B)
Full Cdk motif (pS/TPxR/K) phosphopeptides
Other phosphopeptides
C
unchanged
>2x enriched in swe1Δ cdc55Δ
% difference
97
48
- 48
-97
D
LIGHT
HEAVY
2 repeats (A and B) switching isotopes/ protocol
G1 arrest
release 65 min
mitosis
release 45 min
G2
mix
Phosphopeptide enrichment and mass spectrometry
H/L enrichment score (A)
H/L enrichment score (B)
pTPxR/K
pTP
pS/pTP
pSP
Other motifs
Time after G1 release (min)
%
ss
ls
90 12 53
80 19 71
70 55 34
65 85 3
60 65 0
55 43 0
50 9 0
45 3 0
40 1 0
35 0 0
30 0 0
G1 0 0
M
G2
1c 2c
ss, short spindles; ls, long spindles

Figure S5

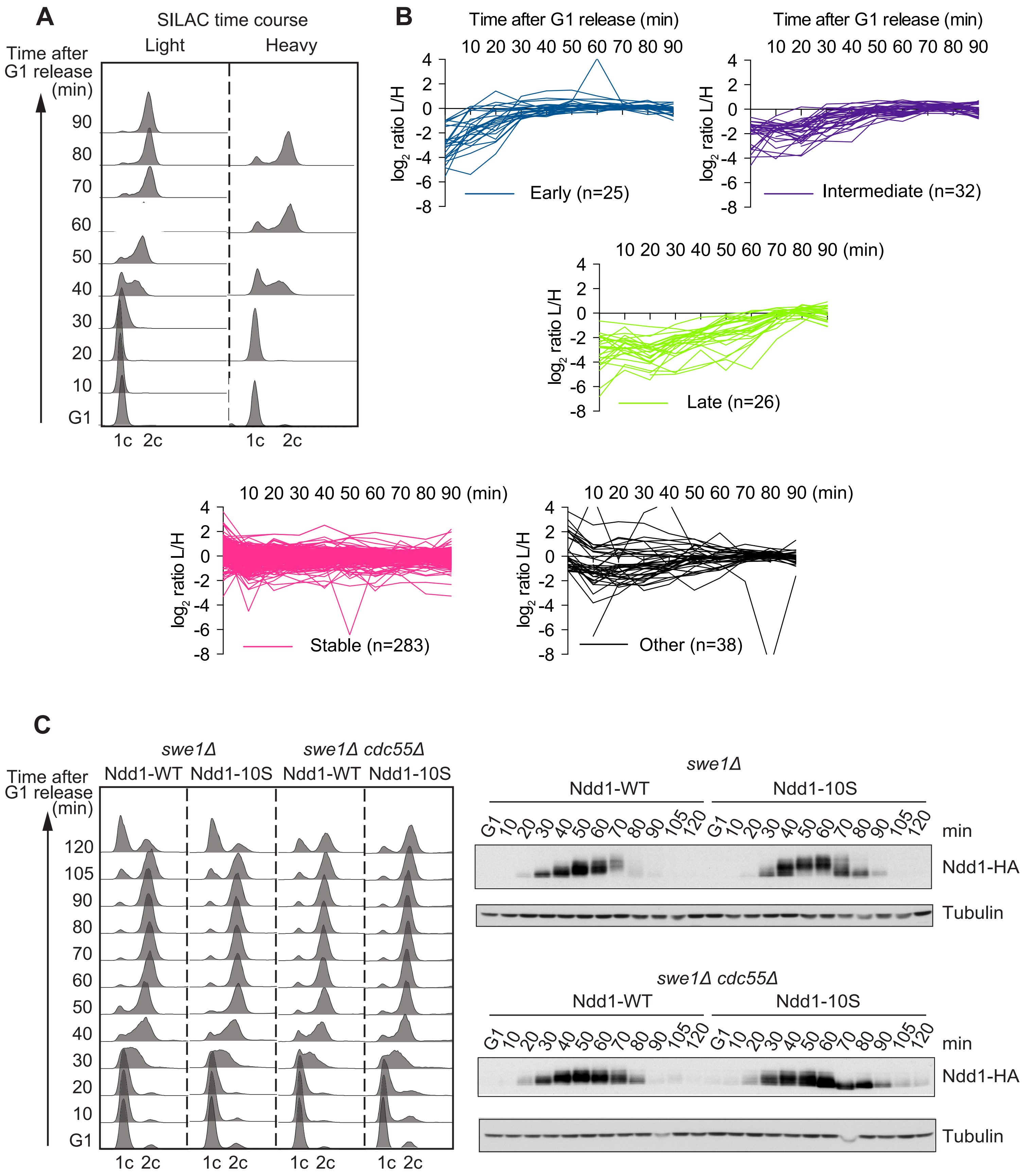

A
SILAC time course
Light
Heavy
Time after G1 release (min)
90
80
70
60
50
40
30
20
10
G1
1c 2c
1c 2c
B
Time after G1 release (min)
10 20 30 40 50 60 70 80 90
log2 ratio L/H
Early (n=25)
Intermediate (n=32)
10 20 30 40 50 60 70 80 90 (min)
Late (n=26)
Stable (n=283)
Other (n=38)
C
swe1Δ
swe1Δ cdc55Δ
Ndd1-WT
Ndd1-10S
Time after G1 release (min)
120
105
90
80
70
60
50
40
30
20
10
G1
1c 2c
G1 10 20 30 40 50 60 70 80 90 105 120
min
Ndd1-HA
Tubulin

Figure S6

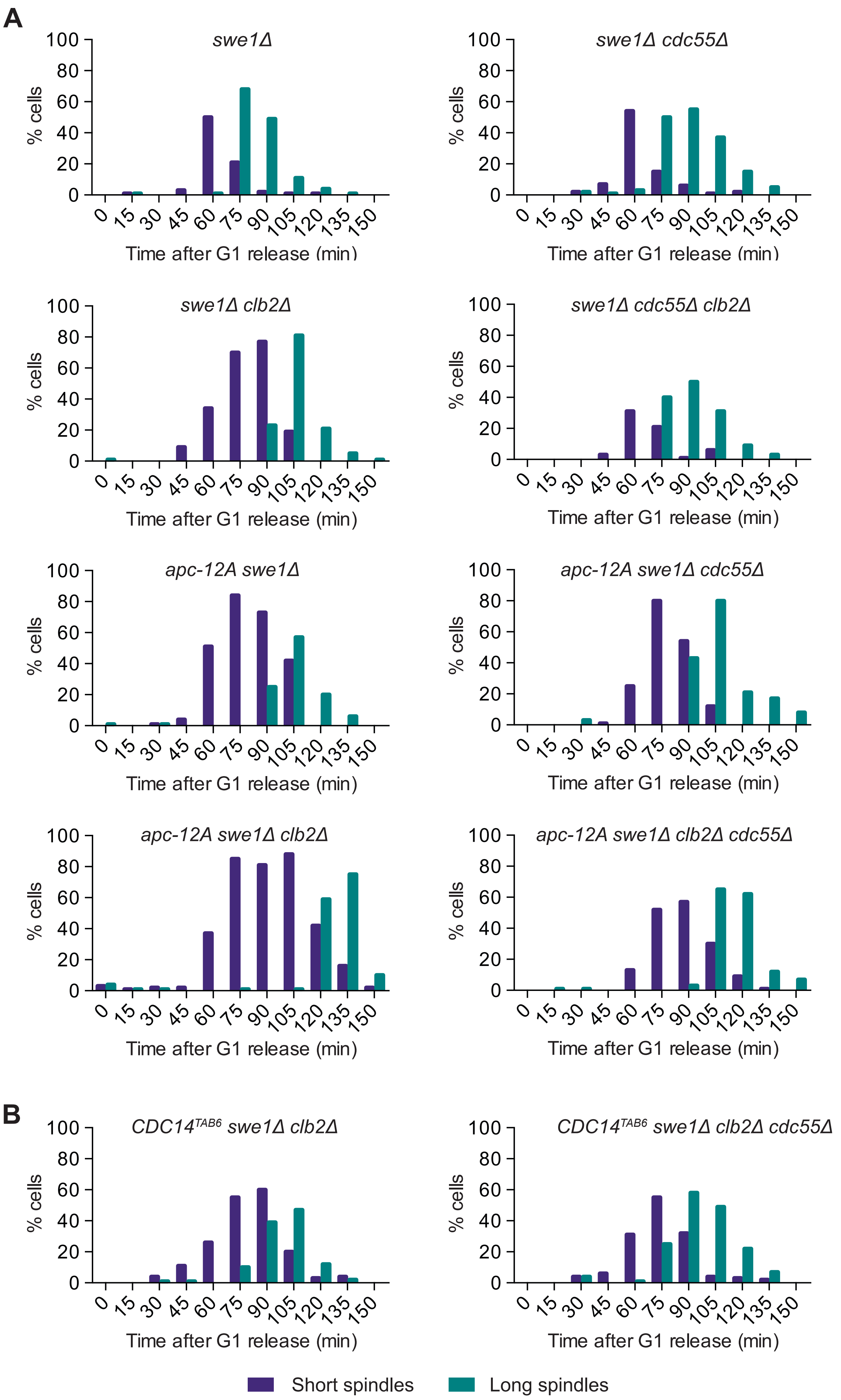
A
swe1Δ
swe1Δ cdc55Δ
swe1Δ clb2Δ
swe1Δ cdc55Δ clb2Δ
apc-12A swe1Δ
apc-12A swe1Δ cdc55Δ
apc-12A swe1Δ clb2Δ
apc-12A swe1Δ clb2Δ cdc55Δ
B
CDC14TAB6 swe1Δ clb2Δ
CDC14TAB6 swe1Δ clb2Δ cdc55Δ
% cells
0
20
40
60
80
100
0 15 30 45 60 75 90 105 120 135 150
Time after G1 release (min)
Short spindles
Long spindles

Figure S7

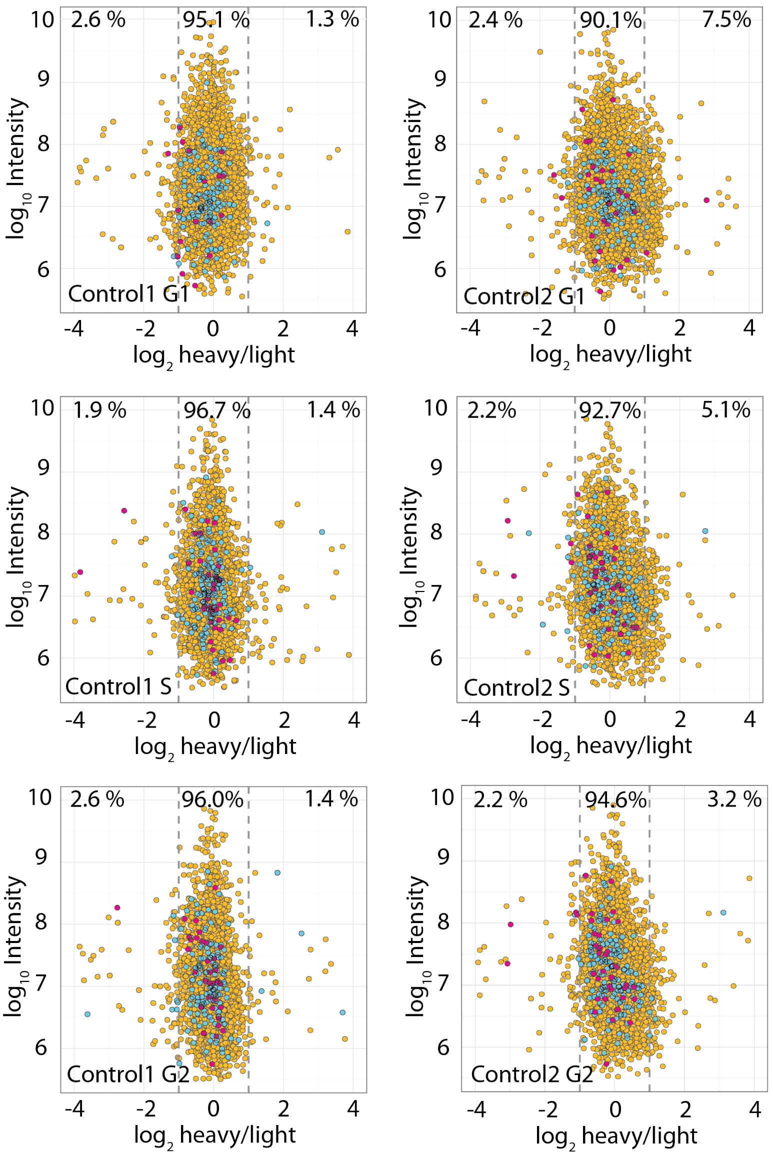

2.6 %
95.1 %
1.3 %
Control1 G1
2.4 %
90.1%
7.5%
Control2 G1
1.9 %
96.7 %
1.4 %
Control1 S
2.2%
92.7%
5.1%
Control2 S
2.6 %
96.0%
1.4 %
Control1 G2
2.2 %
94.6%
3.2 %
Control2 G2
log10 Intensity
log2 heavy/light

Figure S8

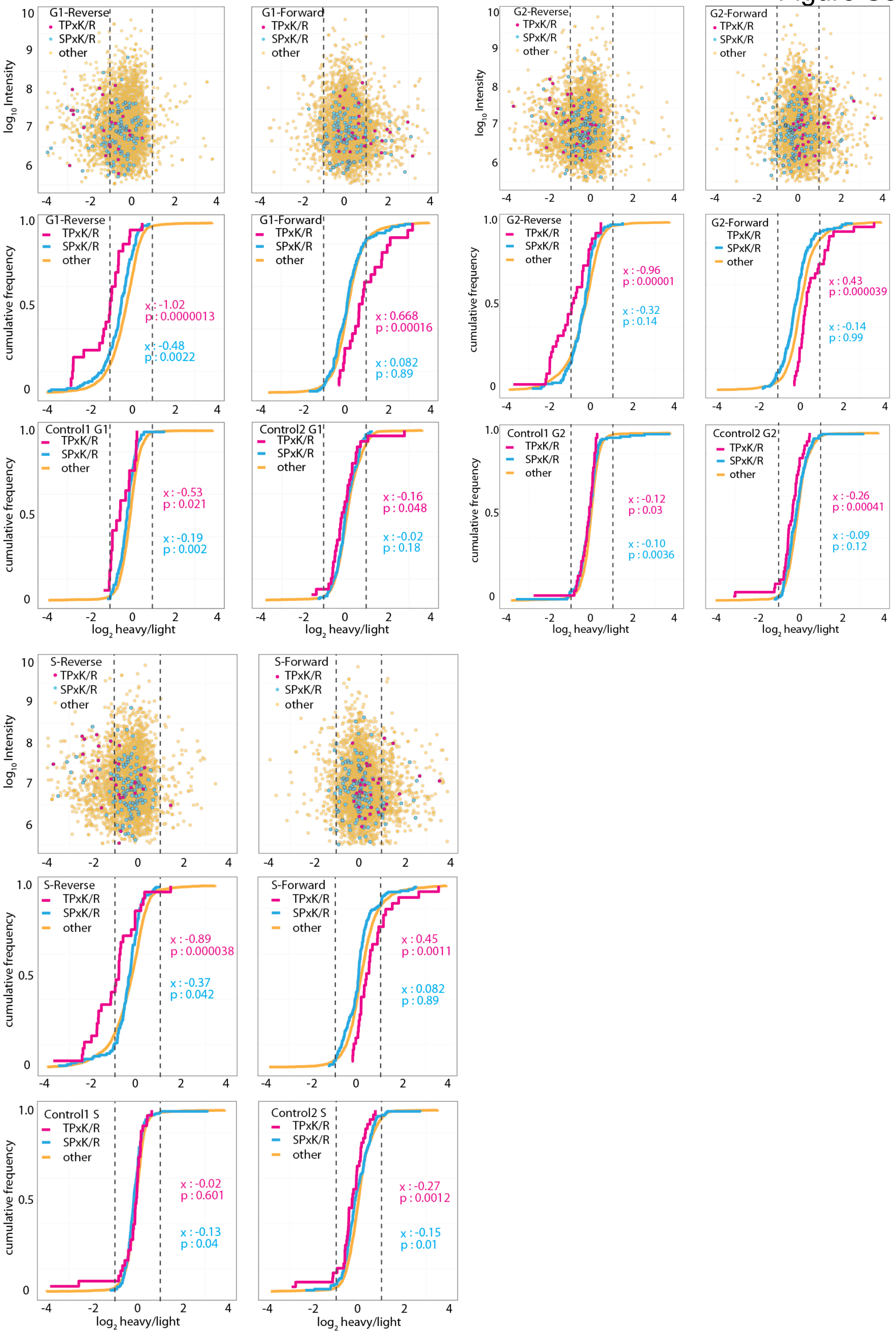

G1-Reverse
TPxK/R
SPxK/R
other
G1-Forward
G2-Reverse
G2-Forward
log10 Intensity
G1-Reverse
x: -1.02
p: 0.0000013
x: -0.48
p: 0.0022
G1-Forward
x: 0.668
p: 0.00016
x: 0.082
p: 0.89
G2-Reverse
x: -0.96
p: 0.00001
x: -0.32
p: 0.14
G2-Forward
x: 0.43
p: 0.000039
x: -0.14
p: 0.99
cumulative frequency
Control1 G1
x: -0.53
p: 0.021
x: -0.19
p: 0.002
Control2 G1
x: -0.16
p: 0.048
x: -0.02
p: 0.18
Control1 G2
x: -0.12
p: 0.03
x: -0.10
p: 0.0036
Ccontrol2 G2
x: -0.26
p: 0.00041
x: -0.09
p: 0.12
log2 heavy/light
S-Reverse
S-Forward
S-Reverse
x: -0.89
p: 0.000038
x: -0.37
p: 0.042
S-Forward
x: 0.45
p: 0.0011
x: 0.082
p: 0.89
Control1 S
x: -0.02
p: 0.601
x: -0.13
p: 0.04
Control2 S
x: -0.27
p: 0.0012
x: -0.15
p: 0.01

SUPPLEMENTAL FIGURE LEGENDS

Figure S1. Comparison of cell cycle progression between *swe1Δ* and wild type cells, Related to Figure 1

swe1Δ and wild type cells were arrested in G1 by pheromone α-factor treatment, released to progress through a synchronous cell cycle before re-arrest in the next G1 phase by α-factor re-addition. Cell cycle progression was monitored by FACS analysis of DNA content and by scoring the fraction of cells with short (<2 μm) and long (≥2 μm) spindles. This revealed that wild type and *swe1Δ* cells progress through S phase at similar rates, but that *swe1Δ* cells enter mitosis and progress into anaphase approximately 10-15 minutes earlier than wild type cells, consistent with published observations (Wang and Burke, 1997).

Figure S2. Additional FACS analyses and repeat of the Ndd1 phosphorylation analysis including internal timing controls, Related to Figure 1

(A) FACS profiles to monitor cell cycle progression during the experiments, shown in Figure 1B, in which the kinetics of Ask1 and Ndd1 phosphorylation were analyzed by western blotting.

(B) Repeat of the Ndd1 phosphorylation timecourse analysis. As in Figure 1B, but Ask1 phosphorylation and the levels of Sic1, Clb5 and Clb2 were also analyzed and served as internal timing controls. This confirmed advanced Ndd1 phosphorylation, relative to Ask1, in cells lacking $PP2A^{Cdc55}$. Tubulin served as a loading control.

Figure S3. Comparison of cell cycle-regulated Cdk phosphorylation in the presence and absence of $PP2A^{Cdc55}$, using antibodies raised against phosphorylated Cdk consensus peptides, Related to Figure 1

Cells were arrested in G1 by pheromone α-factor treatment, released to progress through a synchronous cell cycle, before re-arrest in the next G1 phase by α-factor re-addition. Cell extracts were prepared at the indicated times and analyzed by western blotting using the indicated antibodies. While these antibodies were raised against either phosphoserine or phosphothreonine-containing peptides, their ability to discriminate between phosphoserine and phosphothreonine is not known. Tubulin served as a loading control. Cell cycle stages were confirmed by FACS analysis of DNA content. A discernable cell cycle advance and intensity increase of bands reactive against all three antibodies is apparent in the absence of $PP2A^{Cdc55}$.

Figure S4. Analysis of the $PP2A^{Cdc55}$-dependent phosphoproteomes at three timepoints during synchronous cell cycle progression and a G2/M comparison of the phosphoproteomes in wild type cells, Related to Figure 2

(A) FACS analysis of DNA content from the two inverse SILAC experiments documented in Figure 2, illustrating the cell cycle stage at which the timepoints were taken. Immunofluorescence samples were also analyzed to confirm that the G2 sample was taken before mitotic spindle formation (not shown).

(B) The heavy/light (H/L) ratio of all phosphopeptides in the 2 repeats, (A) and (B) as in Figure 2, are shown. Full Cdk consensus motif-containing phosphopeptides are highlighted. Their increased levels in the absence of $PP2A^{Cdc55}$ become apparent in the upper left quadrant of the diagram.

(C) Sequence logos of pSP/pTP-sites, enriched greater than two-fold in the absence of $PP2A^{Cdc55}$, or unchanged, were prepared as in Figure 2D, but the iceLogo algorithm (Colaert et al., 2009) was used that takes the species-specific probability of amino acid occurrence into account.

(D) Phosphoproteome comparison between G2 phase and mitosis. A schematic of the SILAC experiment is shown, indicating the times when G2 and mitosis samples were taken after the staggered synchronous release of cultures grown in light and heavy amino acids. Cell cycle progression was monitored by FACS analysis of DNA content and by scoring the fraction of cells with short and long spindles. 3,124 phosphopeptides were reproducibly identified in both cultures in both repeats. A 2D annotation enrichment plot is shown of motifs preferentially phosphorylated in mitosis. Other phosphorylation consensus sites are indicated in grey. Compare Figure 2C for details. The strongest enrichment in mitosis, compared to G2, was observed for the threonine-directed full Cdk consensus motif pTPxR/K. See also Dataset 3 for the abridged mass spectrometry data.

Figure S5. Phosphoproteome analysis during synchronous cell cycle progression and the effect of PP2A^{Cdc55} on Ndd1-10S phosphorylation, Related to Figures 3 and 4

(A) FACS analysis of DNA content to monitor cell cycle progression during the SILAC time course experiment shown in Figure 3.

(B) The traces of all pSP and pTP sites are shown, subdivided by whether they reached their maximal phosphorylation at early (10 – 30 minutes, G1-S), intermediate (40 – 60 minutes, S-G2) or late (60 – 90 minutes, mitosis) timepoints. Stable phosphopeptides are also shown, as well as those in the 'other' category.

(C) PP2A^{Cdc55} has little influence on the phosphorylation timing of Ndd1-10S. Strains of the indicated genotypes, expressing Ndd1 or Ndd1-10S, progressed synchronously through the cell cycle following α-factor arrest and release. The Ndd1 phosphorylation status was analyzed by western blotting. Tubulin served as a loading control.

Figure S6. APC and Net1 phosphorylation only partly explain the role of PP2A^{Cdc55} in ordering S-phase and mitosis, Related to Figure 5

Cells of the indicated genotypes were arrested in G1 by pheromone α-factor treatment, released to progress through a synchronous cell cycle before re-arrest in the next G1 phase by α-factor re-addition. Cell cycle progression was monitored by scoring the percentage of cells with short or long spindles.

(A) The mitotic delay caused by the *apc-12A* mutations, even more pronounced in the *clb2Δ* background, was substantially reduced by *cdc55* deletion, indicating that mitotic control exerted by PP2A^{Cdc55} acts on targets in addition to the APC.

(B) Similarly, *CDC14^{TAB6}* was less able than *cdc55Δ* to advance mitosis in the *clb2Δ* background, indicating that PP2A^{Cdc55} acts on targets in addition to the Cdc14 inhibitor Net1.

Figure S7. Intensity plots of the SILAC ratios in the six control experiments, Related to the STAR*Methods

The $\log_2$ ratio versus $\log_{10}$ intensity of each phosphorylation site was plotted for each of the six control experiments in which the same strains were labeled with light or heavy amino acids at three cell cycle stages. The two-fold change intervals are indicated by the dashed lines and the percentages of phosphorylation sites contained within this interval is displayed in the panels. Each datapoint represents a quantified phosphorylation site. Phosphorylated TPxK/R sites are indicated in pink, SPxK/R sites in blue.

Figure S8. Cumulative frequency graphs comparing the differences in threonine and serine phosphorylation in the absence of PP2A^{Cdc55}, Related to the STAR*Methods

In these cumulative frequency graphs, all phosphrylation sites are ordered as a function of their change between the 'heavy' and 'light' samples. The median $\log_2$ SILAC ratio difference 'x' for both TPxK/R and SPxK/R categories, compared to all other phosphosites is given. Also given is the p-value of a Wilcoxon-Mann-Whitney test, indicating whether the category is enriched at high or low values, as determined by a 1D annotation enrichment test (Cox and Mann, 2012). This revealed highly significant enrichment of Cdk threonine site phosphorylation in the absence of PP2A^{Cdc55}.

Dataset 1. Abridged mass spectrometry data comparing phosphosite abundance between a wild type and a *cdc55Δ* strain at three cell cycle stages, G1, S and G2, Related to Figure 2.

Dataset 2. Abridged mass spectrometry data of the experiment to follow phosphosite abundance during synchonous cell cycle progression of a wild type strain, Related to Figure 3.

Dataset 3. Abridged mass spectrometry data comparing the phosphosite abundance in a wild type strain between G2 and M, Related to Figure S4D

SUPPLEMENTAL TABLES

Table S1 Overview of peptide counts in the phosphoproteome datasets, Related to Figures 2 and 3

Figure 2

Top left quadrant	Peptides	Proteins
G1	1286	588
S	1286	589
G2	1305	585

Others	Peptides	Proteins
G1	2208	924
S	1962	838
G2	1998	868

	Number of phosphopeptides			
	G1	S	G2	Total
pTPxK/R	19	22	27	35
pTP	223	206	208	320
pS/TP	914	846	851	1209
pSP	691	640	634	889
pSPxR/K	106	121	127	143
pS/TPxR/K	125	145	155	178
Aurora	--	49	50	61

	Phosphopeptides			
	All	S/TP-containing	enriched >2X	S/TP enriched >2X
G1	3122	914	192	117
S	2901	846	210	28
G2	2867	851	237	143
Total	4589	1203		197

Figure 3

	Peptides	Proteins	pSP peptides	pTP peptides
All	405	298		
Early	25	16	22	3
Intermediate	32	30	26	6
Late	26	22	12	14
Stable	295	209	230	53
Oher	27	21	30	8

Table S2 Yeast strains used in this study, Related to the STAR*Methods section.

Strain name	Genotype	Background	Source
**5059**	*MATa ASK1-HA3::TRP1 swe1Δ::HIS3*	W303	This study
**5060**	*MATa ASK1-HA3::TRP1 swe1Δ::HIS3 cdc55Δ::LEU2*	W303	This study
**5057**	*MATa SLI15-HA3::TRP1 swe1Δ::HIS3*	W303	This study
**5058**	*MATa SLI15-HA3::TRP1 swe1Δ::HIS3 cdc55Δ::LEU2*	W303	This study
**5049**	*MATa NDD1-HA3::TRP1 swe1Δ::HIS3*	W303	This study
**5050**	*MATa NDD1-HA3::TRP1 swe1Δ::HIS3 cdc55Δ::LEU2*	W303	This study
**5048**	*MATa NDD1-HA3::TRP1*	W303	This study
**5231**	*MATa NDD1-HA3::TRP1 swe1Δ::HIS3 ASK1-myc18::URA3*	W303	This study
**5232**	*MATa NDD1-HA3::TRP1 swe1Δ::HIS3 cdc55Δ::LEU2 ASK1-myc18::URA3*	W303	This study
**2369 (YAL6B)**	*MATa arg4Δ(YHR018C)::kan^R lys1Δ(YIR034C)::kan^R*	S288C	Gruhler *et al.* (2005)
**4639**	*MATa arg4Δ(YHR018C)::kan^R lys1Δ(YIR034C)::kan^R swe1Δ::HIS3*	S288C	This study
**4640**	*MATa arg4Δ(YHR018C)::kan^R lys1Δ(YIR034C)::kan^R swe1Δ::HIS3 cdc55Δ::LEU2*	S288C	This study
**5230**	*MATa NDD1-HA3::TRP1 ASK1-myc18::URA3*		
**5233**	*MATa NDD1-10S-HA3::TRP1 ASK1-myc18::URA3*		
**5126**	*MATa NDD1-10S-HA3::TRP1*	W303	This study
**5127**	*MATa NDD1-10S-HA3::TRP1 swe1Δ::HIS3*	W303	This study
**5128**	*MATa NDD1-10S-HA3::TRP1 swe1Δ::HIS3 cdc55Δ::LEU2*	W303	This study
**5093**	*MATa NDD1-HA3::TRP1 swe1Δ::HIS3 clb2Δ::URA3*	W303	This study
**5095**	*MATa NDD1-HA3::TRP1 swe1Δ::HIS3 cdc55Δ::LEU2 clb2Δ::URA3*	W303	This study
**5076**	*MATa swe1Δ::HIS3 CDC55-PK3::TRP1 PPH21-PK3::LEU2*	W303	This study
**5067**	*MATa swe1Δ::HIS3 GAL1pr-CDC55-PK3::LEU2 (x1)*	W303	This study
**5068**	*MATa swe1Δ::HIS3 GAL1pr-CDC55-PK3::LEU2 (x2)*	W303	This study
**5078**	*MATa swe1Δ::HIS3 GAL1pr-CDC55-PK3::LEU2 (x2) GAL10pr-GAL4 GAL1pr-PPH21-PK3::ADE2*	W303	This study
**5065 (ADR6624)**	*MATa cdc16-6A::TRP1 cdc27-5A::kan^R cdc23-A::hyg^R bar1Δ*	W303	Gift from A. Rudner
**5071**	*MATa cdc16-6A::TRP1 cdc27-5A::kan^R cdc23-A::hyg^R bar1Δ swe1Δ::HIS3*	W303	This study
**5072**	*MATa cdc16-6A::TRP1 cdc27-5A::kan^R cdc23-A::hyg^R bar1Δ swe1Δ::HIS3 cdc55Δ::LEU2*	W303	This study
**5073**	*MATa cdc16-6A::TRP1 cdc27-5A::kan^R cdc23-A::hyg^R bar1Δ swe1Δ::HIS3 clb2Δ::URA3*	W303	This study
**5074**	*MATa cdc16-6A::TRP1 cdc27-5A::kan^R cdc23-A::hyg^R bar1Δ swe1Δ::HIS3 cdc55Δ::LEU2 clb2Δ::URA3*	W303	This study
**5097**	*MATa CDC14^{TAB6}-PK6::TRP1 swe1Δ::HIS3 clb2Δ::URA3*	W303	This study
**5098**	*MATa CDC14^{TAB6}-PK6::TRP1 swe1Δ::HIS3 cdc55Δ::LEU2 clb2Δ::URA3*	W303	This study

Table S3 Experimental design table for the experiment in Figure 2, comparing phosphosite abundance in the presence and absence of PP2A^{Cdc55}. The total number of phosphorylation sites quantified for each individual mixture is shown in each cell, as well as the number of quantified sites that contained a phosphorylated serine or threonine within a full Cdk consensus motif (S/TPxK/R), Related to the STAR*Methods section.

			heavy					
			WT			*cdc55Δ*		
			G1	S	G2	G1	S	G2
light	WT	G1	**Control1 G1**<br>total: 4240<br>SPxK/R: 122<br>TPxK/R: 17			**G1-F**<br>total: 4190<br>SPxK/R: 135<br>TPxK/R: 23		
		S		**Control1 S**<br>total: 4117<br>SPxK/R: 157<br>TPxK/R: 35			**S-F**<br>total: 4000<br>SPxK/R: 146<br>TPxK/R: 32	
		G2			**Control1 G2**<br>total: 3453<br>SPxK/R: 139<br>TPxK/R: 34			**G2-F**<br>total: 3746<br>SPxK/R: 149<br>TPxK/R: 37
	cdc55Δ	G1	**G1-R**<br>total: 3945<br>SPxK/R: 122<br>TPxK/R: 26			**Control2 G1**<br>total: 4188<br>SPxK/R: 125<br>TPxK/R: 31		
		S		**S-R**<br>total: 3610<br>SPxK/R: 135<br>TPxK/R: 30			**Control2 S**<br>total: 3907<br>SPxK/R: 151<br>TPxK/R: 39	
		G2			**G2-R**<br>total: 3709<br>SPxK/R: 144<br>TPxK/R: 32			**Control2 G2**<br>total: 3904<br>SPxK/R: 153<br>TPxK/R: 40
