# Supplementary material for: PP2ACdc55 Phosphatase Imposes Ordered Cell-Cycle Phosphorylation by Opposing Threonine Phosphorylation
Source: Mol Cell. 2017 Feb 2;65(3):393–402.e3. doi: 10.1016/j.molcel.2016.12.018 (PMC5296252; doi:10.1016/j.molcel.2016.12.018)
Supplement: Document S2. Article plus Supplemental Information [file mmc5.pdf]

Article
Molecular Cell

PP2A^{Cdc55} Phosphatase Imposes Ordered Cell-Cycle Phosphorylation by Opposing Threonine Phosphorylation

Graphical Abstract

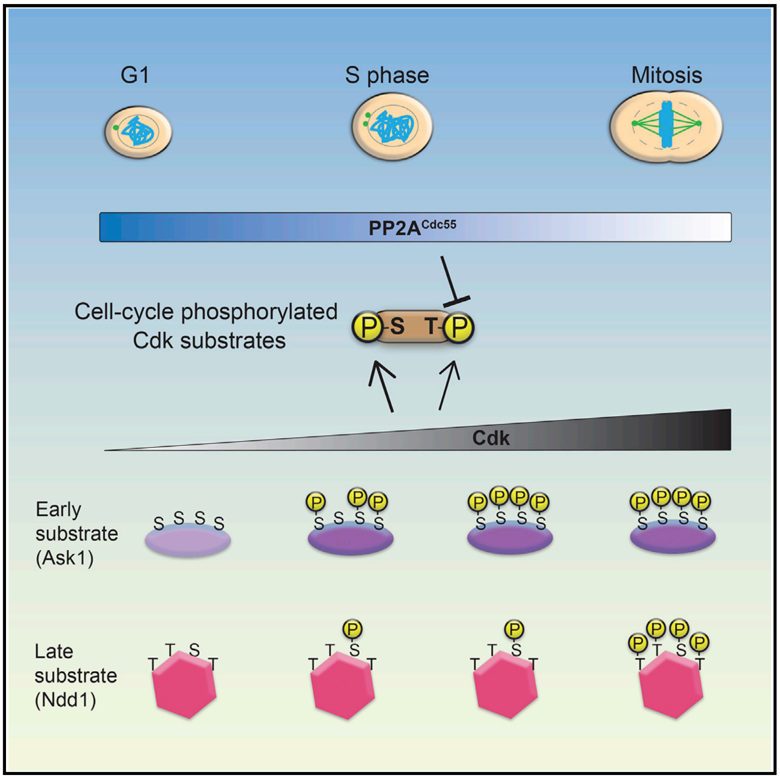

Highlights

- The phosphatase PP2A^{Cdc55} counteracts Cdk phosphorylation during the cell cycle
- It does so specifically on threonine, but not serine, phosphorylation sites
- Consequently, threonine phosphorylation is a late event in the cell cycle
- Thereby, PP2A^{Cdc55} contributes to setting the timing of mitosis

Authors

Molly Godfrey, Sandra A. Touati, Meghna Kataria, Andrew Jones, Ambrosius P. Snijders, Frank Uhlmann

Correspondence

frank.uhlmann@crick.ac.uk

In Brief

The eukaryotic cell cycle comprises a robustly ordered series of phosphorylation events. Godfrey et al. show that the phosphatase PP2A^{Cdc55} counteracts and thereby delays cell-cycle phosphorylation by cyclin-dependent kinase specifically on threonine, but not serine, phosphorylation sites. This reveals an ordering principle that might be also relevant in other signaling networks.

Godfrey et al., 2017, Molecular Cell *65*, 393–402
February 2, 2017 © 2016 The Author(s). Published by Elsevier Inc.
http://dx.doi.org/10.1016/j.molcel.2016.12.018

Molecular Cell
Article

CellPress

PP2A^{Cdc55} Phosphatase Imposes Ordered Cell-Cycle Phosphorylation by Opposing Threonine Phosphorylation

Molly Godfrey,[1,3] Sandra A. Touati,[1] Meghna Kataria,[1] Andrew Jones,[2] Ambrosius P. Snijders,[2] and Frank Uhlmann[1,4,*]
[1]Chromosome Segregation Laboratory
[2]Mass Spectrometry Proteomics Science Technology Platform
The Francis Crick Institute, London NW1 1AT, UK
[3]Present address: Developmental Biology Division, Department of Biology, Utrecht University, 3584 CH Utrecht, the Netherlands
[4]Lead Contact
*Correspondence: frank.uhlmann@crick.ac.uk
http://dx.doi.org/10.1016/j.molcel.2016.12.018

SUMMARY

In the quantitative model of cell-cycle control, progression from G1 through S phase and into mitosis is ordered by thresholds of increasing cyclin-dependent kinase (Cdk) activity. How such thresholds are read out by substrates that respond with the correct phosphorylation timing is not known. Here, using the budding yeast model, we show that the abundant PP2A^{Cdc55} phosphatase counteracts Cdk phosphorylation during interphase and delays phosphorylation of late Cdk substrates. PP2A^{Cdc55} specifically counteracts phosphorylation on threonine residues, and consequently, we find that threonine-directed phosphorylation occurs late in the cell cycle. Furthermore, the late phosphorylation of a model substrate, Ndd1, depends on threonine identity of its Cdk target sites. Our results support a model in which Cdk-counteracting phosphatases contribute to cell-cycle ordering by imposing Cdk thresholds. They also unveil a regulatory principle based on the phosphoacceptor amino acid, which is likely to apply to signaling pathways beyond cell-cycle control.

INTRODUCTION

The cell-division cycle is made up of an ordered series of events that ensure faithful replication and accurate partitioning of the genetic material. A major impetus for cell-cycle progression stems from the phosphorylation of numerous target proteins by the master cyclin-dependent kinase (Cdk) (Morgan, 2007). Cdk substrates whose phosphorylation triggers DNA replication (Tanaka et al., 2007; Zegerman and Diffley, 2007), as well as many substrates that are important for entry into and progression through mitosis (Errico et al., 2010; Ubersax et al., 2003), are known, but how the correct substrate phosphorylation timing is defined, such that DNA replication occurs before mitosis, is not well understood. Sequential ordering of phosphorylation events is in part thought to arise from transcriptional waves of G1-, S phase-, and mitosis-specific cyclins that confer substrate specificity to the kinase. For example, G1- and S phase-specific cyclins recognize hydrophobic substrate motifs that increase kinase-substrate affinity and thereby enhance the phosphorylation rate of certain substrates (Bhaduri and Pryciak, 2011; Brown et al., 1999; Kõivomägi et al., 2011).

While cyclin specificity provides an apparently straightforward ordering principle, cell-cycle progression is remarkably resilient to its abolition. For example, the ordering of S phase and mitosis remains uncompromised if S phase cyclins are replaced by mitotic cyclins in both budding yeast and vertebrates (Hu and Aparicio, 2005; Moore et al., 2003). In another example, all budding yeast G1-specific cyclins can be removed so long as their role in upregulating S phase cyclin expression is compensated for (Tyers, 1996). What is more, ordered cell-cycle progression is maintained with a single source of cyclin-Cdk activity in fission yeast (Coudreuse and Nurse, 2010; Fisher and Nurse, 1996). This has led to the proposal that the quantitative increase, rather than qualitative differences, in Cdk activity controls ordered Cdk substrate phosphorylation (Stern and Nurse, 1996). How such a quantitative increase of Cdk activity is read out by substrates that respond by phosphorylation at the correct time is not known. In budding yeast, Cdk phosphorylates its many substrates with widely varying efficiencies (Loog and Morgan, 2005), but there is no obvious correlation between phosphorylation efficiency and phosphorylation timing. Thus, ordered cell-cycle phosphorylation is not simply defined by kinase affinity for its substrates, as the best substrates are not necessarily those that are phosphorylated first.

If Cdk activity is chemically inhibited at various cell-cycle stages, Cdk substrate phosphorylation is rapidly lost (Holt et al., 2009; Liakopoulos et al., 2003). This suggests that potent Cdk-counteracting phosphatase(s) are active in cells at most times. By reversing phosphorylation, such Cdk-counteracting phosphatases could set thresholds that Cdk activity must overcome to achieve net substrate phosphorylation (Uhlmann et al., 2011). Whether and how Cdk-counteracting phosphatases indeed act during interphase to order cell-cycle

CrossMark

This is an open access article under the CC BY license (http://creativecommons.org/licenses/by/4.0/).

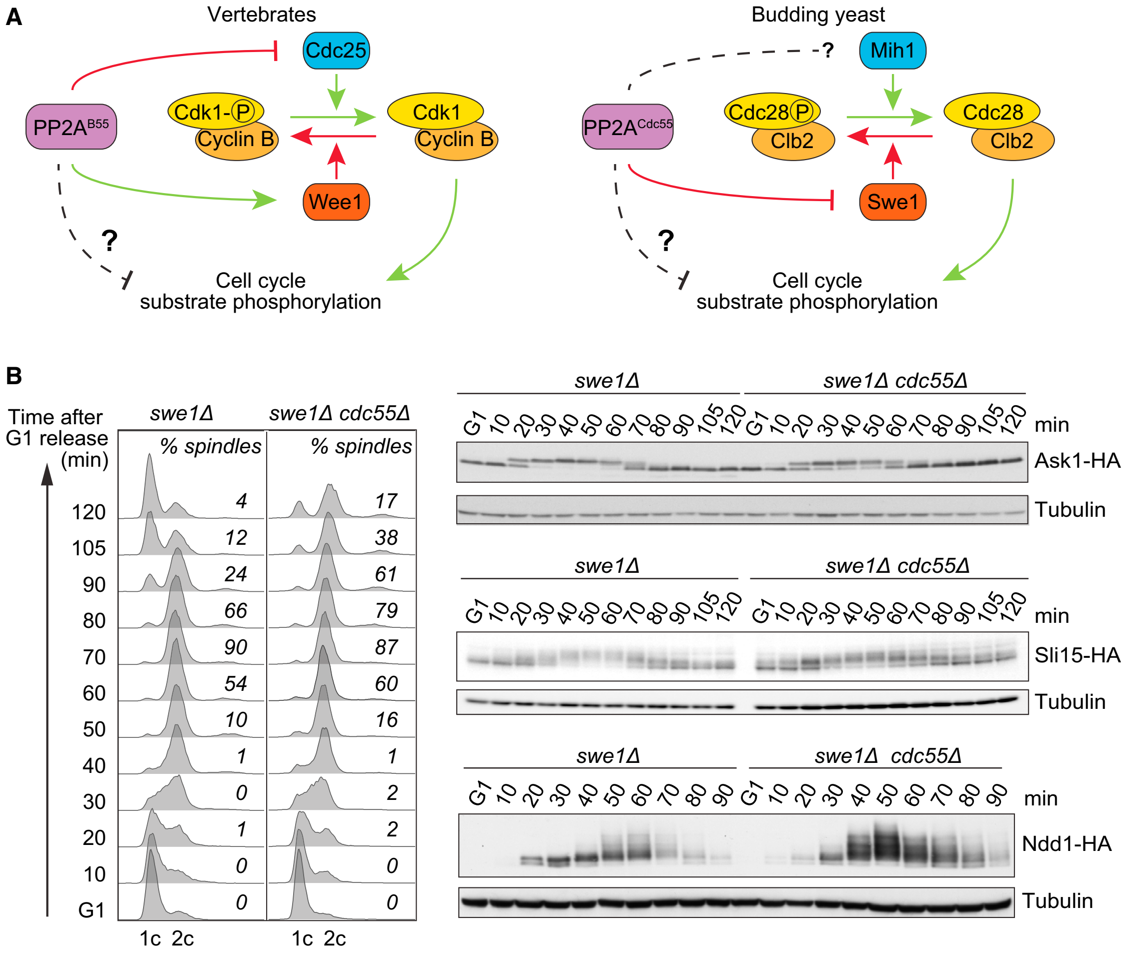

Figure 1. PP2A^{Cdc55} Delays Cdk Substrate Phosphorylation Independently of Impacting Cdk Tyrosine Phosphorylation

(A) Simplified schematics of how PP2A^{Cdc55} is thought to affect Cdk activity in vertebrates and budding yeast (Chica et al., 2016; Harvey et al., 2011; Kinoshita et al., 1993; Lee et al., 1991; Minshull et al., 1996). See also Figure S1 for a comparison of cell-cycle progression in wild-type and *swe1Δ* strains.

(B) PP2A^{Cdc55} delays cell-cycle phosphorylation of a subset of Cdk substrates. *swe1Δ* and *swe1Δ cdc55Δ* cells were arrested in G1 by pheromone α-factor treatment and released to progress through a synchronous cell cycle before re-arrest in the next G1 phase by α-factor re-addition. Protein extracts were prepared at the indicated times from strains in which Ask1, Sli15, or Ndd1 was fused to a hemagglutinin (HA) epitope tag. Cell-cycle progression was monitored by fluorescence-activated cell sorting (FACS) analysis of DNA content. Shown is a representative profile of the Sli15-HA experiment, as well as representative scores of cells containing mitotic (short or long) spindles.

See also Figure S2A for FACS analyses of the other experiments, Figure S2B for a repeat of the Ndd1 phosphorylation analysis, including internal timing controls, and Figure S3 for cell-cycle phosphorylation analysis using antibodies raised against phosphorylated Cdk consensus peptides.

phosphorylation is not yet known. It is also unknown how such phosphatases might differentiate early from late phosphorylated Cdk targets to set a higher Cdk threshold for phosphorylation of the latter. Here, we analyze the contribution of the budding yeast PP2A^{Cdc55} phosphatase to the dynamics of cell-cycle phosphorylation. We find that PP2A^{Cdc55} indeed counteracts and thereby delays Cdk phosphorylation, but only on a subset of substrates. These are enriched for threonines as the phosphoacceptor sites, consistent with the known biochemical PP2A substrate preference. Our results portray cell-cycle phosphorylation as a dynamic process in which the steady-state level of phosphorylation is under control of the changing activity ratio of Cdk and its counteracting phosphatases.

RESULTS

PP2A^{Cdc55} Phosphatase Imposes Late Cdk Substrate Phosphorylation

We analyzed a possible contribution of the abundant PP2A^{Cdc55} phosphatase to the regulation of Cdk substrate phosphorylation during the budding yeast cell cycle. PP2A^{Cdc55} and its orthologs in vertebrates have been studied for their impact on cell-cycle progression by controlling inhibitory Cdk tyrosine phosphorylation (Chica et al., 2016; Harvey et al., 2011; Kinoshita et al., 1993; Lee et al., 1991; Minshull et al., 1996) (Figure 1A). Budding yeast cells lacking PP2A^{Cdc55} show gross morphological defects and poor growth due to attenuated Cdk activity (Wang and Burke, 1997). To disambiguate a possible role of

PP2A^{Cdc55} in dephosphorylating Cdk substrates, we therefore employed a budding yeast strain background lacking the Cdk tyrosine kinase Swe1 (*swe1Δ*). Without inhibitory Cdk phosphorylation, *swe1Δ* cells enter mitosis slightly earlier than wild-type cells but show otherwise undisturbed ordering of cell-cycle events (Harvey and Kellogg, 2003) (Figure S1, available online). Notably, absence of PP2A^{Cdc55} no longer causes morphological defects in cells lacking Swe1 (Wang and Burke, 1997).

To examine the impact of PP2A^{Cdc55} on the kinetics of Cdk substrate phosphorylation, we compared the electrophoretic mobility changes of three known Cdk targets, Ask1, Sli15, and Ndd1 (Li and Elledge, 2003; Pereira and Schiebel, 2003; Reynolds et al., 2003), during synchronous cell-cycle progression of *swe1Δ* and *swe1Δ cdc55Δ* cells (Figure 1B). Ask1 is phosphorylated early in the cell cycle as soon as cells enter S phase. Its phosphorylation timing was unaltered in the absence of PP2A^{Cdc55}. In contrast, both Sli15 and Ndd1 are phosphorylated later, around the time of mitotic entry. Phosphorylation of both late Cdk substrates was markedly advanced in the absence of PP2A^{Cdc55} (Figure 1B). Sli15 phosphorylation was advanced by at least 20 min, with some increased phosphorylation evident already in G1-arrested cells. Ndd1 phosphorylation was advanced by ~10 min, a substantial difference during the short budding yeast cell cycle. These phosphorylation timing changes were reproducible between repeats of the experiment. Figure S2 shows a repeat of the Ndd1 phosphorylation time course, including additional cell-cycle markers as internal timing controls. This confirmed that Ndd1 phosphorylation is advanced in the absence of PP2A^{Cdc55}.

We also probed cell extracts with antibodies raised against phosphorylated Cdk consensus peptides. This provided further evidence for both advanced as well as increased Cdk phosphorylation in cells lacking PP2A^{Cdc55} (Figure S3). Despite these examples of advanced Cdk phosphorylation, the overall kinetics of interphase progression were similar between the two strains, while mitotic exit and cytokinesis were delayed in the absence of PP2A^{Cdc55}. This suggests that PP2A^{Cdc55} counteracts and thereby delays phosphorylation of some, but not all, Cdk substrates during interphase.

PP2A^{Cdc55} Counteracts Threonine Phosphorylation

To obtain a global view of Cdk substrates affected by PP2A^{Cdc55}, we quantitatively compared the phosphoproteomes of *swe1Δ* and *swe1Δ cdc55Δ* cells at three time points during synchronous cell-cycle progression in G1, S, and G2 (Figures 2A and S4A). We used stable isotope labeling with amino acids in culture (SILAC) followed by phosphopeptide enrichment and mass spectrometry. This allowed us to compare the abundance of 4,589 phosphosites on 1,309 proteins in the presence or absence of PP2A^{Cdc55} (for details of the peptide and protein numbers identified in the three cell-cycle stages, see Table S1). Plotting the heavy/light ratio of phosphosites from two repeats with inverse isotope labeling allowed reliable identification of phosphosites enriched in the absence of PP2A^{Cdc55}. These are found in the upper left quadrants of the corresponding 2D scatterplots (Figure 2B). This analysis revealed that PP2A^{Cdc55} counteracts protein phosphorylation throughout interphase in G1, S, and G2 phases. Numerous phosphosites adhering to the minimal (pS/pTP) or full (pS/pTPxR/K; Figure S4B) Cdk consensus sites were among those enriched in the absence of PP2A^{Cdc55}.

As a control, to assess the variability introduced by the SILAC method, we differentially labeled two cultures of the same wild-type or mutant strains, respectively. This revealed a small degree of variation, with the vast majority of phosphosites (94.3%) detected with a less than 2-fold difference between the two labeling conditions (see the Quantification and Statistical Analysis). We therefore used a greater than 2-fold change as our selection criterion to compile phosphosites enriched in the absence of PP2A^{Cdc55}, including an acceptable false discovery rate. Among those, in samples taken in S and G2 phase, we found several Ndd1-derived sites. At the same time, other Cdk phosphosites were not affected by the absence of PP2A^{Cdc55}, clustering around the origin of the enrichment plots. This confirms that PP2A^{Cdc55} counteracts phosphorylation of some, but not all, Cdk substrates.

We next explored whether PP2A^{Cdc55} shows phosphorylation site preferences when selecting its substrates. To this end, we assigned each phosphosite to one or more phosphorylation site consensus motifs. We then performed a 2D annotation enrichment analysis, which tests for the enrichment of motifs at either high or low SILAC ratios, based on their rank (Cox and Mann, 2012). This revealed that Cdk phosphorylation was significantly affected by PP2A^{Cdc55}. In particular, this was the case for phosphothreonine-containing, but not phosphoserine-containing, Cdk sites (Figure 2C). In a complementary approach, we generated a sequence logo of the 197 Cdk phosphosites that increased over 2-fold in the absence of PP2A^{Cdc55} and compared it to the logo of the 713 sites that increased by less or remained unchanged (Figure 2D). This again demonstrated that PP2A^{Cdc55} preferentially affects threonine phosphorylation, a result that was statistically highly significant (see the Quantification and Statistical Analysis). An in vitro preference for threonine dephosphorylation has been noted during the early biochemical characterization of PP2A using peptide substrates (Agostinis et al., 1990). Recently, a preferential impact of PP2A on threonine phosphorylation was observed in protein extracts prepared from HeLa cells (Cundell et al., 2016). Our results show that this translates into a pronounced in vivo substrate preference.

Substrate recognition by human PP2A^{Cdc55} was suggested to involve a polybasic recognition determinant (Cundell et al., 2016). Our above sequence logo of PP2A^{Cdc55}-dependent phosphosites did not reveal a similar requirement (Figure 2D). When we analyzed the surrounding of PP2A^{Cdc55}-regulated phosphosites using an algorithm that takes the species-specific probability of amino acid occurrence into account (Colaert et al., 2009), a slight enrichment of positively charged amino acids became detectable (Figure S4C). An acidic patch on Cdc55 that has been implicated in substrate interactions (Xu et al., 2008) could be a reason for this preference. The same argument might explain why the impact of PP2A^{Cdc55} was quantitatively greatest on full Cdk consensus sites (Figure 2C), which include an oppositely charged basic residue.

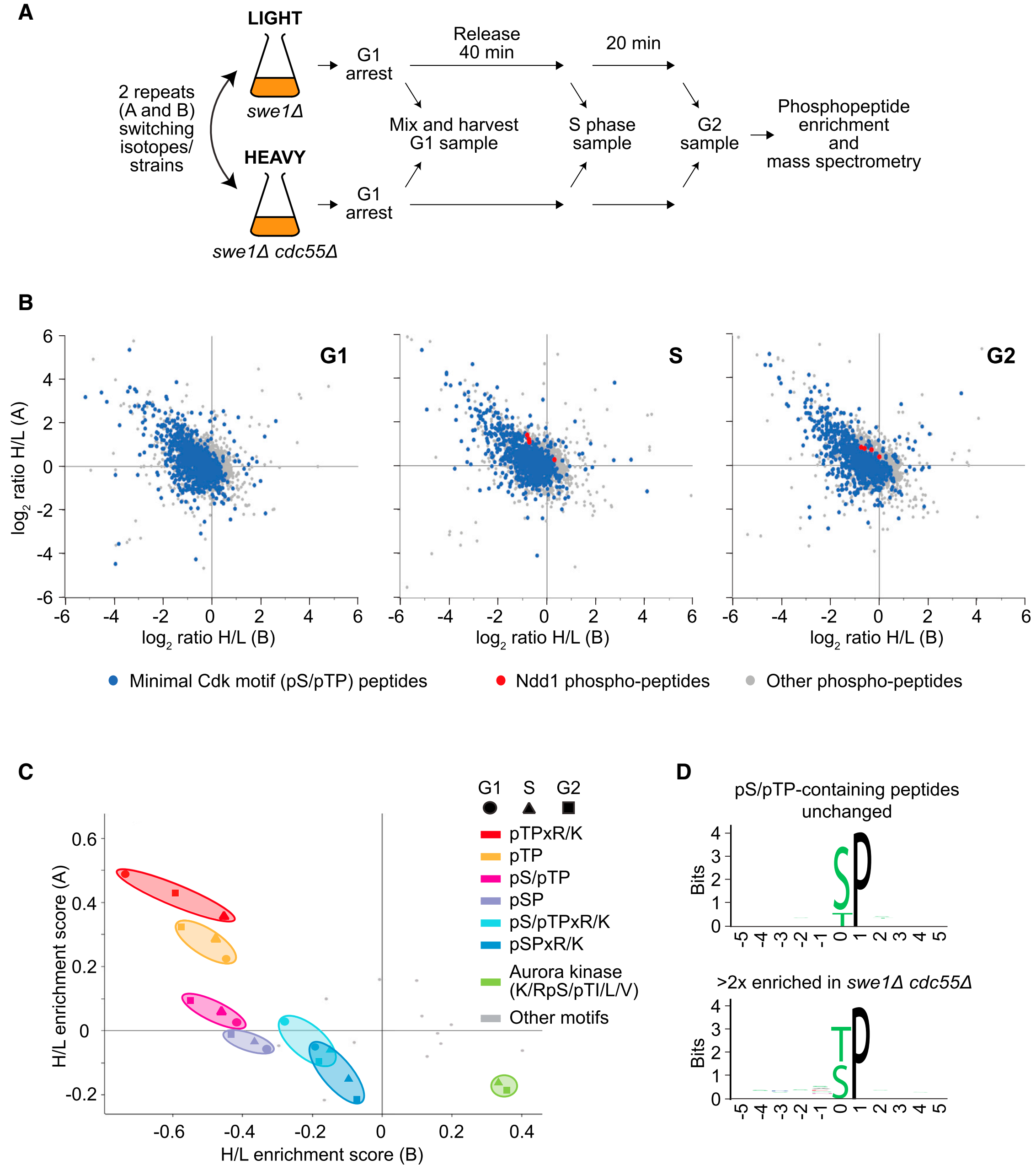

Figure 2. Increased Threonine-Directed Phosphorylation in Cells Lacking PP2A^{Cdc55}

(A) Schematic of the experiment to compare PP2A^{Cdc55}-dependent phosphoproteomes at three time points during synchronous cell-cycle progression.

(B) The normalized heavy/light (H/L) ratio of all phosphosites in two repeats of the experiment, (A) and (B), is shown. 4,589 phosphosites on 1,309 proteins were quantified in both repeats, many at more than one cell-cycle stage (compare Table S1). Minimal Cdk motif-containing phosphosites are highlighted, as well as those derived from Ndd1.

(C) 2D annotation enrichment analysis of phosphosite motifs whose abundance changed significantly in the absence of PP2A^{Cdc55} ($p < 0.02$, using an adapted Wilcoxon Mann-Whitney test as described previously; Cox and Mann, 2012).

(D) Sequence logos of pSP/pTP sites enriched greater than 2-fold in the absence of PP2A^{Cdc55} or unchanged.

See also Figure S4A for FACS analysis to confirm the cell-cycle state of cells at the three time points, Figure S4B for a 2D annotation of full Cdk consensus sites, Figure S4C for sequence logos taking into account the species-specific probability of amino acid occurrence, Table S1 for a summary of peptide counts, and Data S1 for the abridged mass spectrometry data.

Among all other phosphorylation site motifs analyzed, the Aurora kinase motif stood out as showing significantly decreased phosphorylation in the absence of PP2A^{Cdc55} (Figure 2C). This could be a secondary consequence of increased Cdk phosphorylation on its regulatory Sli15 subunit (Figure 1B), which is thought to attenuate Aurora kinase activity (Zimniak et al., 2012).

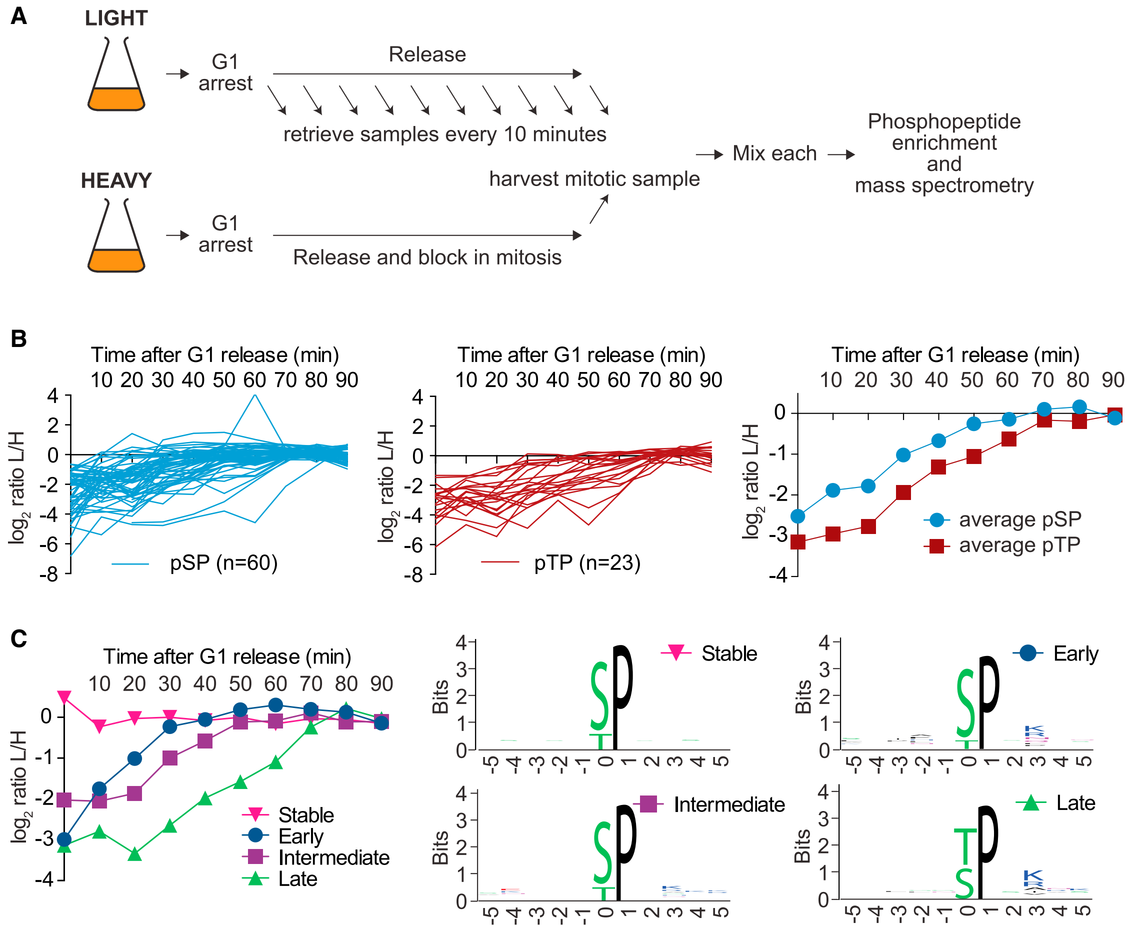

Figure 3. Threonine-Directed Phosphorylation Occurs Late in the Cell Cycle

(A) Schematic of the experiment to analyze time-resolved phosphoproteome changes during synchronous cell-cycle progression. See also Figure S4D for an experiment comparing phosphoproteome changes between G2 and M.

(B) The intensity profiles of all increasing pSP and pTP sites are plotted over the course of cell-cycle progression and normalized to the intensity at 90 min (left and middle). The averages of all profiles are compared (right).

(C) The average intensity profiles of all pSP/pTP sites, divided into sequential early, intermediate, and late categories (reaching their maximal levels at 10–30, 40–60, and 70–90 min, respectively) as well as stable phosphosites, are plotted together with the sequence logos surrounding the phosphorylation site in each category.

See also Figures S5A and S5B for cell-cycle analysis and for the aggregate traces of all the sites, subdivided into phosphorylation timing categories, Table S1 for a summary of peptide counts, and Data S2 for the abridged mass spectrometry data.

Threonine Phosphorylation Is a Late Cell-Cycle Event

If PP2A^{Cdc55} specifically targets phosphothreonines to set an increased Cdk threshold for their phosphorylation, threonines should be phosphorylated later in the cell cycle compared to serines. To address whether this is the case, we used SILAC followed by mass spectrometry to compare the phosphoproteomes of G2 and mitosis, using a pair of sequential samples taken from wild-type cells that synchronously passed through the cell cycle. 2D annotation enrichment analysis, incorporating a repeat experiment using inverse labeling, revealed that the phosphorylation site motif most strongly enriched in mitosis, compared to G2, was pTPxR/K (Figure S4D). This corresponds to the threonine-directed motif whose phosphorylation is most strongly counteracted by PP2A^{Cdc55}. Thus, PP2A^{Cdc55}-regulated phosphorylation events indeed correspond to those that are reserved until late in the cell cycle.

To gain a time-resolved picture of serine and threonine phosphorylation, we performed a mass-spectrometry-based time course experiment. Samples were taken at 10-min intervals from a synchronous culture grown in light amino acids that progressed from G1 to anaphase. Each sample was combined with an aliquot of a metaphase sample grown in heavy amino acids (Figure 3A). This allowed us to follow the abundance of 405 phosphoserine-proline (pSP)- or phosphothreonine-proline (pTP)-containing phosphosites during cell-cycle progression. 83 (20%) of these showed a greater than 2-fold increase between G1 and mitosis, the rest were either stable or displayed unclear patterns (Figure S5). Categorizing the increasing phosphosites into pSPs and pTPs and plotting their abundance

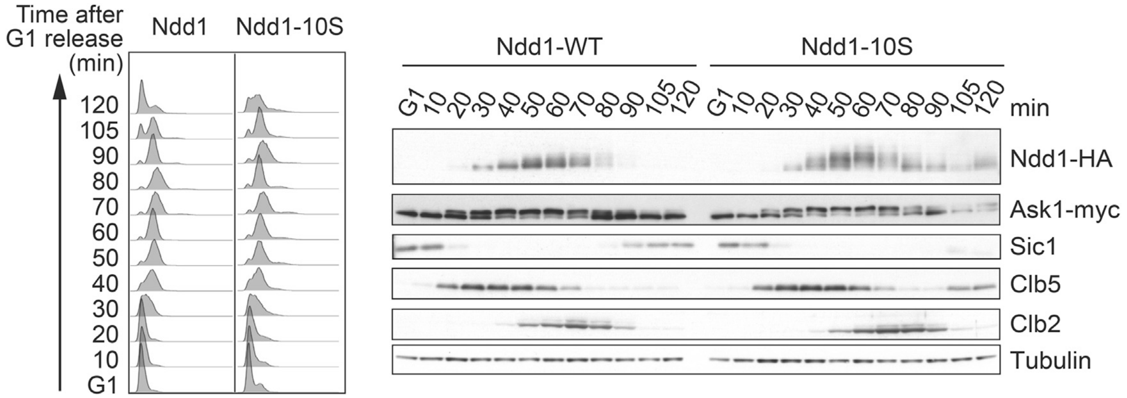

Figure 4. Threonine Identity of the Cdk Acceptor Sites Defines Late Ndd1 Phosphorylation

Wild-type strains, carrying wild-type Ndd1 or Ndd1-10S, progressed synchronously through the cell cycle following α-factor arrest and release. The Ndd1 phosphorylation status was analyzed by western blotting. Ask1 phosphorylation and the levels of Sic1, Clb5, and Clb2 served as internal timing controls. Tubulin was the loading control.

See also Figure S5C, which shows that PP2A^{Cdc55} retains only a small effect on the phosphorylation timing of Ndd1-10S.

over time made apparent that pSP sites indeed increased earlier in the cell cycle compared to pTP sites (Figure 3B). This became additionally evident from plotting the average phosphorylation kinetics of all the increasing phosphosites within these two categories. pTP sites started out in G1 at a lower relative level of phosphorylation and reached their peak of phosphorylation 10–20 min after pSP sites.

In a complementary analysis, we subdivided all Cdk sites into early, intermediate, late, or stable categories depending on the time they reached their maximal level of phosphorylation. Early sites included known early Cdk targets (e.g., Whi5 and Stb1), while late sites included known late substrates (e.g., Ndd1 and Net1), thus validating our assignments. The averaged phosphorylation kinetics of each group over time is shown in Figure 3C (aggregate traces of all phosphosites can be found in Figure S5B). We then created sequence logos of the phosphosites in each category. This revealed that only 3 out of 25 early phosphorylated sites were pTP, which increased over 4-fold to 14 out of 26 late phosphosites (Table S1). A χ^2 test across the time categories confirmed that late phosphorylated sites are significantly enriched for pTP compared to the other categories ($p = 0.0013$, corrected for multiple testing). Together, these results reveal that phosphothreonines are preferentially kept underphosphorylated by PP2A^{Cdc55} and that this correlates with their late phosphorylation during the cell cycle.

Threonine Identity Confers Late Ndd1 Phosphorylation

We next wanted to establish a causal relationship between the nature of the phosphoacceptor amino acid and its phosphorylation timing. Therefore, we asked whether threonine identity not only correlates with but also is responsible for late Cdk substrate phosphorylation. To do this, we replaced ten threonine Cdk target sites in Ndd1 (Reynolds et al., 2003) with serines. In an otherwise wild-type strain background, phosphorylation of the resulting Ndd1-10S protein was both advanced and increased during cell-cycle progression (Figure 4). Ask1 phosphorylation, which remained unaffected, served as an internal timing comparison. This confirms that the threonine nature of the Cdk target sites on Ndd1 is instrumental in restricting their phosphorylation to mitosis. Consistent with the idea that PP2A^{Cdc55} delays Ndd1 phosphorylation by targeting threonines, PP2A^{Cdc55} impacted on the phosphorylation timing of Ndd1-10S to a smaller degree when compared to wild-type Ndd1 (Figure S5C).

Accelerated Cell-Cycle Progression in the Absence of PP2A^{Cdc55}

If delayed threonine phosphorylation contributes to the ordering of cell-cycle progression, one should expect accelerated mitotic entry in the absence of PP2A^{Cdc55}, when these late phosphorylation events are advanced. However, the relative timing of S phase and mitosis was similar in *swe1Δ* and *swe1Δ cdc55Δ* cells (Figure 1B). This could be because PP2A^{Cdc55} acts redundantly with another phosphatase that targets a complementary set of Cdk substrates or redundantly with another ordering principle. Alternatively, the timing of mitosis, which is advanced in the *swe1Δ* background, cannot be further advanced due to intrinsic time requirements of the underlying cell biological events (Georgi et al., 2002). To differentiate between these scenarios, we aimed to compensate for increased Cdk activity in *swe1Δ* cells by removing Clb2, one of the four mitotic cyclins (Fitch et al., 1992). This reversed the accelerated mitosis seen in *swe1Δ* cells (Figure S6A). In synchronized cultures, we monitored DNA content as a sign of S phase, spindle formation as a marker for G2, and the beginning of spindle elongation as indicator for mitosis when the anaphase-promoting complex (APC) is activated as Cdk activity reaches its peak (Figure 5). *cdc55* deletion in the *swe1Δ clb2Δ* background did not change the timing of S and G2 phases but caused a notable advance of anaphase onset. Ndd1 phosphorylation was also shifted to earlier time points. These observations suggest that PP2A^{Cdc55} indeed delays the onset of mitosis.

It has been suggested that PP2A^{Cdc55} counteracts APC phosphorylation as part of a Swe1-dependent morphogenesis checkpoint (Lianga et al., 2013). We therefore tested whether APC is also a PP2A^{Cdc55} target that defines mitotic timing.

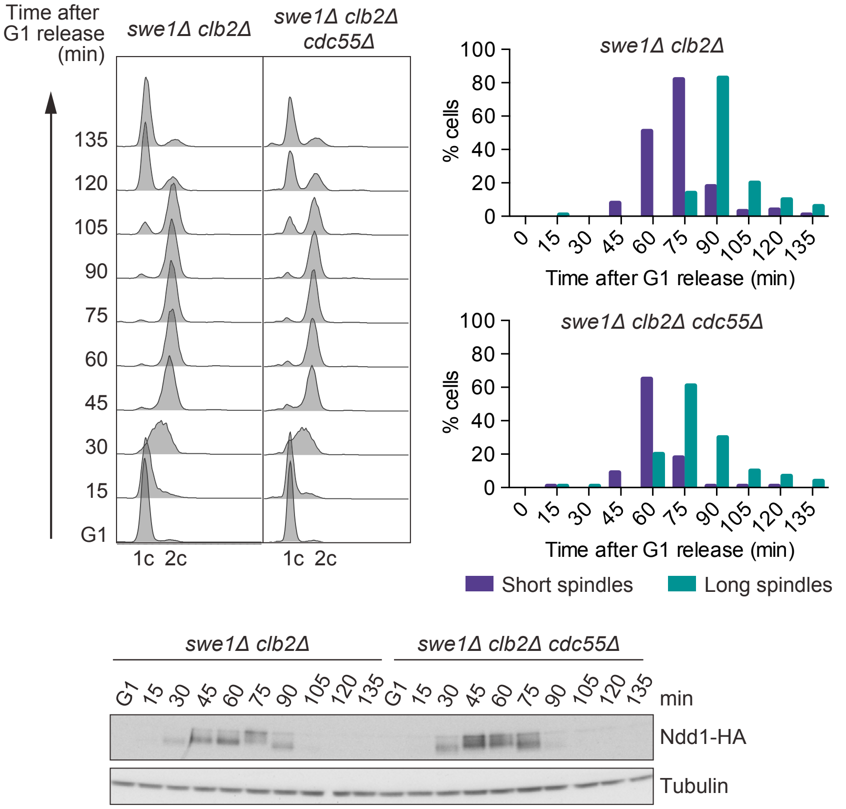

Figure 5. Accelerated Cell-Cycle Progression in the Absence of PP2A^{Cdc55}

Cell-cycle progression in cells lacking Swe1 and Clb2 was compared to cells that additionally lacked PP2A^{Cdc55}. Cells were arrested in G1 by pheromone α-factor treatment and released to progress through a synchronous cell cycle before re-arrest in the next G1 phase by α-factor re-addition. Cell-cycle progression was monitored by FACS analysis of DNA content. The percentage of cells showing short (<2 μm) or long (≥2 μm) spindles is plotted over time. Ndd1 phosphorylation kinetics were analyzed by western blotting. Tubulin served as a loading control.

See also Figure S6, which shows that APC and Net1 phosphorylation can only partly explain the impact of PP2A^{Cdc55} on mitotic timing.

We utilized the *apc-12A* strain (Lianga et al., 2013), in which 12 activatory phosphorylation sites on three APC subunits (including five threonines) are replaced by alanine, thus mimicking a constitutively dephosphorylated state. The consequent mitotic delay in *swe1Δ apc-12A* cells (even more pronounced in *swe1Δ apc-12A clb2Δ* cells) was substantially reduced by *cdc55* deletion (Figure S6A), suggesting that PP2A^{Cdc55} targets substrates in addition to the APC. Another previously characterized PP2A^{Cdc55} target is the Cdc14 phosphatase inhibitor Net1 (Queralt et al., 2006). To test whether premature Net1 phosphorylation and consequent Cdc14 activation advances the cell cycle, we utilized the *CDC14^{TAB6}* allele (Shou and Deshaies, 2002), which similarly advances Cdc14 activation. *CDC14^{TAB6}* only slightly advanced mitosis in *clb2Δ* cells, while *cdc55* deletion in this background further accelerated mitotic progression (Figure S6B). Together, this suggests that APC and Net1 are two of potentially many PP2A^{Cdc55} targets that together define mitotic timing.

PP2A^{Cdc55} Causes a Dosage-Dependent Cell-Cycle Delay

Additional evidence that PP2A^{Cdc55} is a rate-limiting factor that counteracts Cdk phosphorylation during cell-cycle progression came from an experiment using increased PP2A^{Cdc55} levels. Ectopic Cdc55 expression, and co-expression of the PP2A catalytic subunit Pph21, led to a dosage-dependent slowdown of cell-cycle progression (Figures 6A–6C). A delay was apparent both during S phase (DNA replication and bud formation) and more pronounced during mitosis (spindle formation and anaphase onset). Monitoring Cdk-dependent cell-cycle events by western blotting confirmed these observations. Early events, such as Sic1 degradation and Clb5 accumulation, were delayed to a lesser degree than later events, such as Orc6 phosphorylation and Clb2 accumulation (Figure 6D). This suggests that PP2A^{Cdc55} counteracts phosphorylation of substrates involved at multiple stages of the cell cycle. The fact that later events were more strongly affected than earlier events is consistent with the idea that late phosphorylated substrates are more sensitive toward the phosphatase.

DISCUSSION

Our results show that PP2A^{Cdc55} counteracts global Cdk phosphorylation during the budding yeast cell cycle in a manner that is independent of the phosphatase's additional role in regulating Cdk tyrosine phosphorylation. We consider it likely that PP2A^{Cdc55} directly dephosphorylates Cdk substrates and thereby delays their phosphorylation until Cdk activity has risen above a threshold required to shift the phosphorylation equilibrium to the phosphorylated state. A reason to believe that PP2A^{Cdc55} acts at the Cdk substrate level is its threonine selectivity, consistent with the observed in vitro PP2A^{Cdc55} specificity (Agostinis et al., 1990; Cundell et al., 2016). Threonine selectivity goes some way to answer the question how PP2A^{Cdc55} selects its substrates. Additional substrate features are likely to play a role in defining PP2A^{Cdc55} targets. In particular, it will be interesting to investigate how PP2A^{Cdc55} dephosphorylation efficiency is quantitatively defined for each substrate, which we expect will equate to the Cdk threshold that must be surpassed to achieve net phosphorylation. Similar to reduced phosphatase activity, increased kinase activity should accelerate mitotic entry, consistent with what has been observed (Oikonomou and Cross, 2011). A preference of Cdk for serine over threonine, which has been observed in case of vertebrate cyclin B1-Cdk1

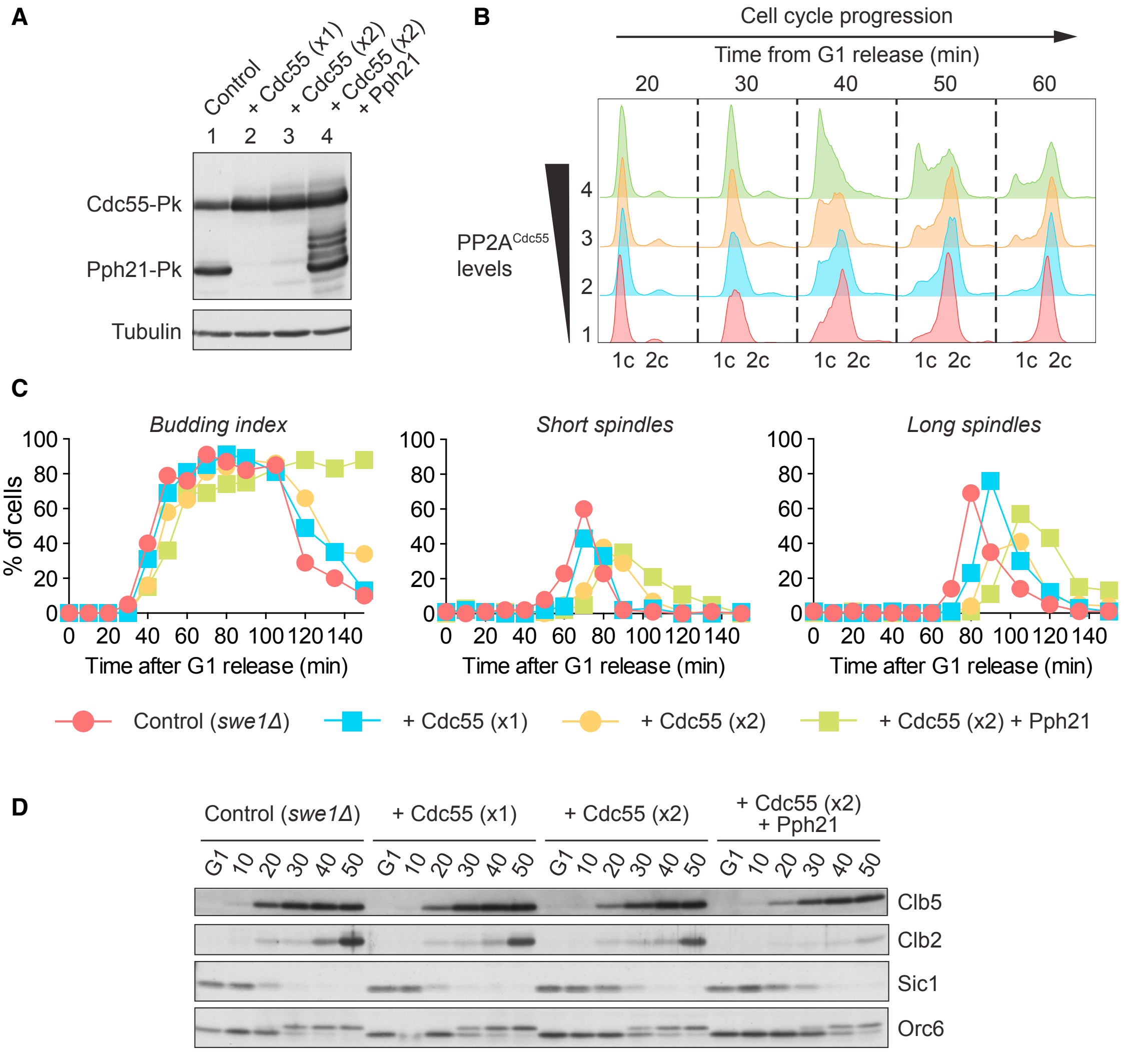

Figure 6. Ectopic PP2A^{Cdc55} Delays Cell-Cycle Progression in a Dosage-Dependent Manner

(A) Increased Cdc55 and Pph21 levels following overexpression of these PP2A^{Cdc55} subunits were analyzed by western blotting from cells growing asynchronously in galactose-containing medium.

(B) Cells shown in (A) were arrested in G1 by α-factor treatment and released to progress through a synchronous cell cycle before re-arrest in the next G1 phase by α-factor re-addition. FACS analysis of DNA content between 20 and 60 min after release is shown to compare S phase progression.

(C) The budding index and fraction of cells with short (<2 μm) or long (≥2 μm) spindles were scored in cell aliquots from the experiment in (B).

(D) Protein extracts were prepared from aliquots of the same cultures, and the abundance and phosphorylation status of the indicated proteins was analyzed by western blotting.

(Suzuki et al., 2015), might in addition contribute to early serine over threonine phosphorylation.

Threonines account for only one-fifth of the cell-cycle-regulated phosphorylation events that we detected in our phosphoproteome analysis. Clearly, ordered phosphorylation extends beyond threonines to the numerous serine-bearing substrates. One possible explanation for ordered serine phosphorylation is that another phosphatase sets substrate-specific thresholds for their phosphorylation. Budding yeast Cdc14 displays serine preference (Bremmer et al., 2012); however, it is thought to be inactive during interphase. Accordingly, our preliminary analysis did not reveal a notable impact of Cdc14 on interphase phosphorylation (data not shown). A yet-to-be-assigned phosphatase might set thresholds for serine phosphorylation during interphase.

Ordering of cell-cycle phosphorylation by Cdk-counteracting phosphatases implies that individual substrates undergo repeated cycles of phosphorylation and dephosphorylation. A similar, if reciprocal, mechanism has been suggested to order substrate dephosphorylation during mitotic exit (Bouchoux and Uhlmann, 2011). While such phosphorylation cycles can be seen as "futile," they allow the cell-cycle control machinery to impose thresholds and timings. The resultant phosphorylation equilibria allow phosphorylation states to remain stable, for example, when cell-cycle progression is halted due to replication or DNA damage checkpoint activation. Depending on the

turnover rate of kinase and phosphatase (Bouchoux and Uhlmann, 2011; Holt et al., 2009; Liakopoulos et al., 2003), tens to hundreds of ATPs might be spent to control a phosphorylation site over the duration of one cell cycle. While sizeable, this expenditure is small compared to the total ATP requirement for synthesizing a protein.

The finding that the nature of the phosphoacceptor amino acid affects its phosphorylation timing opens up an unanticipated level of regulatory potential, not only in cell-cycle control but also other processes that are controlled by phosphorylation. Phosphoacceptor amino acid specificity has been recorded in the cases of several phosphatases, including Cdc14, PP1, and PP2A (Agostinis et al., 1990, 1992; Bremmer et al., 2012). This opens the possibility that other biological processes that are affected by these phosphatases contain a regulatory element dependent on the phosphoacceptor amino acid.

STAR★METHODS

Detailed methods are provided in the online version of this paper and include the following:

- KEY RESOURCES TABLE
- CONTACT FOR REAGENTS
- METHOD DETAILS
  - Yeast Strains and Techniques
  - Western Blotting
  - Immunofluorescence Microscopy
  - Stable Isotope Labeling with Amino Acids In Cell Culture (SILAC) and Mass Spectrometry
- QUANTIFICATION AND STATISTICAL ANALYSIS
  - 2D Annotation Enrichment Analysis, Sequence Logos, and the "Lateness" of Threonine Phosphorylation
  - SILAC Experimental Design and Evaluation whether PP2A^{Cdc55} Preferentially Affects Threonines
- DATA AND SOFTWARE AVAILABILITY

SUPPLEMENTAL INFORMATION

Supplemental Information includes eight figures, three tables, and three data files and can be found with this article online at http://dx.doi.org/10.1016/j.molcel.2016.12.018.

AUTHOR CONTRIBUTIONS

M.G. and F.U. conceived the study, M.G. performed most experiments, S.A.T. and M.K. contributed the Ndd1 phosphorylation acceptor site analysis and performed additional experiments during the revisions, A.J. and A.P.S. performed the phosphoproteome analyses, and M.G. and F.U. wrote the manuscript with input from all authors.

ACKNOWLEDGMENTS

We would like to thank Gavin Kelly for biostatistics support, Adam Rudner for his gift of the *apc-12A* strain, Egon Ogris and Gustav Ammerer for insightful discussions, and Takashi Toda and the members of our laboratory for discussions and critical reading of the manuscript. This work was supported by The Francis Crick Institute, which receives its core funding from Cancer Research UK (FC001198), the UK Medical Research Council (FC001198), and the Wellcome Trust (FC001198).

Received: July 8, 2016
Revised: October 10, 2016
Accepted: December 20, 2016
Published: January 26, 2017

REFERENCES

Agostinis, P., Goris, J., Pinna, L.A., Marchiori, F., Perich, J.W., Meyer, H.E., and Merlevede, W. (1990). Synthetic peptides as model substrates for the study of the specificity of the polycation-stimulated protein phosphatases. Eur. J. Biochem. *189*, 235–241.

Agostinis, P., Derua, R., Sarno, S., Goris, J., and Merlevede, W. (1992). Specificity of the polycation-stimulated (type-2A) and ATP,Mg-dependent (type-1) protein phosphatases toward substrates phosphorylated by P34^{cdc2} kinase. Eur. J. Biochem. *205*, 241–248.

Bhaduri, S., and Pryciak, P.M. (2011). Cyclin-specific docking motifs promote phosphorylation of yeast signaling proteins by G1/S Cdk complexes. Curr. Biol. *21*, 1615–1623.

Bouchoux, C., and Uhlmann, F. (2011). A quantitative model for ordered Cdk substrate dephosphorylation during mitotic exit. Cell *147*, 803–814.

Bremmer, S.C., Hall, H., Martinez, J.S., Eissler, C.L., Hinrichsen, T.H., Rossie, S., Parker, L.L., Hall, M.C., and Charbonneau, H. (2012). Cdc14 phosphatases preferentially dephosphorylate a subset of cyclin-dependent kinase (Cdk) sites containing phosphoserine. J. Biol. Chem. *287*, 1662–1669.

Brown, N.R., Noble, M.E.M., Endicott, J.A., and Johnson, L.N. (1999). The structural basis for specificity of substrate and recruitment peptides for cyclin-dependent kinases. Nat. Cell Biol. *1*, 438–443.

Chica, N., Rozalén, A.E., Pérez-Hidalgo, L., Rubio, A., Novak, B., and Moreno, S. (2016). Nutritional control of cell size by the greatwall-endosulfine-PP2A B55 pathway. Curr. Biol. *26*, 319–330.

Colaert, N., Helsens, K., Martens, L., Vandekerckhove, J., and Gevaert, K. (2009). Improved visualization of protein consensus sequences by iceLogo. Nat. Methods *6*, 786–787.

Coudreuse, D., and Nurse, P. (2010). Driving the cell cycle with a minimal CDK control network. Nature *468*, 1074–1079.

Cox, J., and Mann, M. (2012). 1D and 2D annotation enrichment: a statistical method integrating quantitative proteomics with complementary high-throughput data. BMC Bioinformatics *13* (*Suppl 16*), S12.

Crooks, G.E., Hon, G., Chandonia, J.M., and Brenner, S.E. (2004). WebLogo: a sequence logo generator. Genome Res. *14*, 1188–1190.

Cundell, M.J., Hutter, L.H., Nunes Bastos, R., Poser, E., Holder, J., Mohammed, S., Novak, B., and Barr, F.A. (2016). A PP2A-B55 recognition signal controls substrate dephosphorylation kinetics during mitotic exit. J. Cell Biol. *214*, 539–554.

Errico, A., Deshmukh, K., Tanaka, Y., Pozniakovsky, A., and Hunt, T. (2010). Identification of substrates for cyclin dependent kinases. Adv. Enzyme Regul. *50*, 375–399.

Fisher, D.L., and Nurse, P. (1996). A single fission yeast mitotic cyclin B p34^{cdc2} kinase promotes both S-phase and mitosis in the absence of G_1 cyclins. EMBO J. *15*, 850–860.

Fitch, I., Dahmann, C., Surana, U., Amon, A., Nasmyth, K., Goetsch, L., Byers, B., and Futcher, B. (1992). Characterization of four B-type cyclin genes of the budding yeast *Saccharomyces cerevisiae*. Mol. Biol. Cell *3*, 805–818.

Foiani, M., Marini, F., Gamba, D., Lucchini, G., and Plevani, P. (1994). The B subunit of the DNA polymerase alpha-primase complex in Saccharomyces cerevisiae executes an essential function at the initial stage of DNA replication. Mol. Cell. Biol. *14*, 923–933.

Georgi, A.B., Stukenberg, P.T., and Kirschner, M.W. (2002). Timing of events in mitosis. Curr. Biol. *12*, 105–114.

Gietz, R.D., and Sugino, A. (1988). New yeast-Escherichia coli shuttle vectors constructed with in vitro mutagenized yeast genes lacking six-base pair restriction sites. Gene *74*, 527–534.

Gruhler, A., Olsen, J.V., Mohammed, S., Mortensen, P., Faergeman, N.J., Mann, M., and Jensen, O.N. (2005). Quantitative phosphoproteomics applied to the yeast pheromone signaling pathway. Mol. Cell. Proteomics *4*, 310–327.

Harvey, S.L., and Kellogg, D.R. (2003). Conservation of mechanisms controlling entry into mitosis: budding yeast wee1 delays entry into mitosis and is required for cell size control. Curr. Biol. *13*, 264–275.

Harvey, S.L., Enciso, G., Dephoure, N., Gygi, S.P., Gunawardena, J., and Kellogg, D.R. (2011). A phosphatase threshold sets the level of Cdk1 activity in early mitosis in budding yeast. Mol. Biol. Cell *22*, 3595–3608.

Holt, L.J., Tuch, B.B., Villén, J., Johnson, A.D., Gygi, S.P., and Morgan, D.O. (2009). Global analysis of Cdk1 substrate phosphorylation sites provides insights into evolution. Science *325*, 1682–1686.

Hu, F., and Aparicio, O.M. (2005). Swe1 regulation and transcriptional control restrict the activity of mitotic cyclins toward replication proteins in *Saccharomyces cerevisiae*. Proc. Natl. Acad. Sci. USA *102*, 8910–8915.

Kinoshita, N., Yamano, H., Niwa, H., Yoshida, T., and Yanagida, M. (1993). Negative regulation of mitosis by the fission yeast protein phosphatase ppa2. Genes Dev. *7*, 1059–1071.

Knop, M., Siegers, K., Pereira, G., Zachariae, W., Winsor, B., Nasmyth, K., and Schiebel, E. (1999). Epitope tagging of yeast genes using a PCR-based strategy: more tags and improved practical routines. Yeast *15* (10B), 963–972.

Kõivomägi, M., Valk, E., Venta, R., Iofik, A., Lepiku, M., Morgan, D.O., and Loog, M. (2011). Dynamics of Cdk1 substrate specificity during the cell cycle. Mol. Cell *42*, 610–623.

Lee, T.H., Solomon, M.J., Mumby, M.C., and Kirschner, M.W. (1991). INH, a negative regulator of MPF, is a form of protein phosphatase 2A. Cell *64*, 415–423.

Li, Y., and Elledge, S.J. (2003). The DASH complex component Ask1 is a cell cycle-regulated Cdk substrate in *Saccharomyces cerevisiae*. Cell Cycle *2*, 143–148.

Liakopoulos, D., Kusch, J., Grava, S., Vogel, J., and Barral, Y. (2003). Asymmetric loading of Kar9 onto spindle poles and microtubules ensures proper spindle alignment. Cell *112*, 561–574.

Lianga, N., Williams, E.C., Kennedy, E.K., Doré, C., Pilon, S., Girard, S.L., Deneault, J.-S., and Rudner, A.D. (2013). A Wee1 checkpoint inhibits anaphase onset. J. Cell Biol. *201*, 843–862.

Loog, M., and Morgan, D.O. (2005). Cyclin specificity in the phosphorylation of cyclin-dependent kinase substrates. Nature *434*, 104–108.

Minshull, J., Straight, A., Rudner, A.D., Dernburg, A.F., Belmont, A., and Murray, A.W. (1996). Protein phosphatase 2A regulates MPF activity and sister chromatid cohesion in budding yeast. Curr. Biol. *6*, 1609–1620.

Moore, J.D., Kirk, J.A., and Hunt, T. (2003). Unmasking the S-phase-promoting potential of cyclin B1. Science *300*, 987–990.

Morgan, D. (2007). The Cell Cycle: Principles of Control (New Science Press).

O'Reilly, N., Charbin, A., Lopez-Serra, L., and Uhlmann, F. (2012). Facile synthesis of budding yeast a-factor and its use to synchronize cells of α mating type. Yeast *29*, 233–240.

Oikonomou, C., and Cross, F.R. (2011). Rising cyclin-CDK levels order cell cycle events. PLoS ONE *6*, e20788.

Pereira, G., and Schiebel, E. (2003). Separase regulates INCENP-Aurora B anaphase spindle function through Cdc14. Science *302*, 2120–2124.

Queralt, E., Lehane, C., Novak, B., and Uhlmann, F. (2006). Downregulation of PP2A(^{Cdc55}) phosphatase by separase initiates mitotic exit in budding yeast. Cell *125*, 719–732.

Reynolds, D., Shi, B.J., McLean, C., Katsis, F., Kemp, B., and Dalton, S. (2003). Recruitment of Thr 319-phosphorylated Ndd1p to the FHA domain of Fkh2p requires Clb kinase activity: a mechanism for CLB cluster gene activation. Genes Dev. *17*, 1789–1802.

Rose, M.D., Winston, F., and Hieter, P. (1990). Laboratory Course Manual for Methods in Yeast Genetics (Cold Spring Harbor Laboratory Press).

Shou, W., and Deshaies, R.J. (2002). Multiple t*elophase arrest bypassed (tab)* mutants alleviate the essential requirement for Cdc15 in exit from mitosis in *S. cerevisiae*. BMC Genet. *3*, 4.

Stern, B., and Nurse, P. (1996). A quantitative model for the cdc2 control of S phase and mitosis in fission yeast. Trends Genet. *12*, 345–350.

Suzuki, K., Sako, K., Akiyama, K., Isoda, M., Senoo, C., Nakajo, N., and Sagata, N. (2015). Identification of non-Ser/Thr-Pro consensus motifs for Cdk1 and their roles in mitotic regulation of C2H2 zinc finger proteins and Ect2. Sci. Rep. *5*, 7929.

Tanaka, S., Umemori, T., Hirai, K., Muramatsu, S., Kamimura, Y., and Araki, H. (2007). CDK-dependent phosphorylation of Sld2 and Sld3 initiates DNA replication in budding yeast. Nature *445*, 328–332.

Tyers, M. (1996). The cyclin-dependent kinase inhibitor p40^{SIC1} imposes the requirement for Cln G1 cyclin function at Start. Proc. Natl. Acad. Sci. USA *93*, 7772–7776.

Ubersax, J.A., Woodbury, E.L., Quang, P.N., Paraz, M., Blethrow, J.D., Shah, K., Shokat, K.M., and Morgan, D.O. (2003). Targets of the cyclin-dependent kinase Cdk1. Nature *425*, 859–864.

Uhlmann, F., Bouchoux, C., and López-Avilés, S. (2011). A quantitative model for cyclin-dependent kinase control of the cell cycle: revisited. Philos. Trans. R. Soc. Lond. B Biol. Sci. *366*, 3572–3583.

Vizcaíno, J.A., Csordas, A., del-Toro, N., Dianes, J.A., Griss, J., Lavidas, I., Mayer, G., Perez-Riverol, Y., Reisinger, F., Ternent, T., et al. (2016). 2016 update of the PRIDE database and its related tools. Nucleic Acids Res. *44* (D1), D447–D456.

Wach, A., Brachat, A., Pöhlmann, R., and Philippsen, P. (1994). New heterologous modules for classical or PCR-based gene disruptions in *Saccharomyces cerevisiae*. Yeast *10*, 1793–1808.

Wang, Y., and Burke, D.J. (1997). Cdc55p, the B-type regulatory subunit of protein phosphatase 2A, has multiple functions in mitosis and is required for the kinetochore/spindle checkpoint in Saccharomyces cerevisiae. Mol. Cell. Biol. *17*, 620–626.

Xu, Y., Chen, Y., Zhang, P., Jeffrey, P.D., and Shi, Y. (2008). Structure of a protein phosphatase 2A holoenzyme: insights into B55-mediated Tau dephosphorylation. Mol. Cell *31*, 873–885.

Zegerman, P., and Diffley, J.F.X. (2007). Phosphorylation of Sld2 and Sld3 by cyclin-dependent kinases promotes DNA replication in budding yeast. Nature *445*, 281–285.

Zimniak, T., Fitz, V., Zhou, H., Lampert, F., Opravil, S., Mechtler, K., Stolt-Bergner, P., and Westermann, S. (2012). Spatiotemporal regulation of Ipl1/Aurora activity by direct Cdk1 phosphorylation. Curr. Biol. *22*, 787–793.

STAR★METHODS

KEY RESOURCES TABLE

REAGENT or RESOURCE	SOURCE	IDENTIFIER
Antibodies		
α-Clb5	Santa Cruz	sc20170; RRID: AB_671845
α-Clb2	Santa Cruz	sc9071; RRID: AB_667962
α-Sic1	Santa Cruz	sc50441; RRID: AB_785671
α-Orc6	in house	clone SB49
α-Tub1 (α-tubulin)	Bio-Rad	YOL1/34
α-myc	in house	clone 9E10
α-HA	in house	clone 12CA5
α-Pk	Bio-Rad	SV5-Pk1
α-phosphoMAPK/CDK Substrates	Cell Signaling	#2325; RRID: AB_331820
α-phosphoCDK Substrate Motif	Cell Signaling	#9477
α-phosphothreonine-proline	Cell Signaling	#9391; RRID: AB_331801
Alexa Fluor 594 coupled goat α-rat secondary antibody	Molecular Probes	A11007; RRID: AB_141374
Chemicals, Peptides, and Recombinant Proteins		
Trypsin Gold, Mass Spectrometry Grade	Promega	V5280
rLys-C, Mass Spec Grade	Promega	V1671
Sep-Pak C18 Plus Light Cartridge, 130 mg	Waters	WAT023501
Titansphere TiO, 5 µm	GL Sciences	5020-75000
Critical Commercial Assays		
Liquid Chromatography and Columns		
Dionex UltiMate 3000 HPLC	Thermo Scientific	5041.0010
EASY-Spray C18 column, 75 µm × 50 cm	Thermo Scientific	ES803
Mass spectrometry		
LTQ-Orbitrap Velos	Thermo Scientific	1239200
Deposited Data		
Raw mass spectrometry proteomics data	ProteomeXchange Consortium via the PRIDE partner repository	ProteomeXchange: PXD004461
MaxQuant output mass spectrometry proteomics data files	ProteomeXchange Consortium via the PRIDE partner repository	ProteomeXchange: PXD004461
Experimental Models: Organisms/Strains		
All *Saccharomyces cerevisiae* yeast strains used in this study were of the W303 or S288C background and are listed in Table S2.		N/A
Software and Algorithms		
MaxQuant (version 1.3.0.5)	Open Source	http://www.biochem.mpg.de/5111795/maxquant
Perseus (version 1.4.0.11)	Open Source	http://www.biochem.mpg.de/5111810/perseus
WebLogo	Open Source	weblogo.berkeley.edu/logo.cgi
iceLogo	Open Source	http://iomics.ugent.be/icelogoserver/index.html

CONTACT FOR REAGENTS

Further information and requests for reagents may be directed to, and will be fulfilled by, the corresponding author, Frank Uhlmann (frank.uhlmann@crick.ac.uk).

METHOD DETAILS

Yeast Strains and Techniques

Epitope tagging of endogenous genes and gene deletions were performed by gene targeting using polymerase chain reaction (PCR) products (Knop et al., 1999; Wach et al., 1994). Cdc55 overexpression was achieved by cloning the *CDC55* gene under *GAL1* promoter control into YIplac128 (Gietz and Sugino, 1988), including a Pk epitope tag for detection at the C terminus. The resulting plasmid was integrated into the budding yeast genome at the *LEU2* locus. Colonies were screened for various expression levels, following galactose induction, that are the consequence of different multiplicities of integration. Pph21 co-overexpression was similarly achieved by cloning *PPH21* under *GAL1* promoter control into a vector that also harbored the *GAL4* gene, expressed from the same bidirectional promoter in the *GAL10* direction. Increased Gal4 transcription factor levels facilitate expression of more than one protein under galactose control. The *NDD1-10S* allele was based on a synthetic DNA construct (GeneArt, Life Technologies), that was integrated to replace endogenous *NDD1*. Yeast cultures were grown in rich YP medium supplemented with 2% glucose or with 2% raffinose + 2% galactose (Rose et al., 1990), if not stated otherwise. Cell synchronization using α-factor block and release was performed as described (O'Reilly et al., 2012).

Western Blotting

Protein extracts for western blotting were prepared following cell fixation using trichloroacetic acid, as described by (Foiani et al., 1994), and analyzed by SDS-polyacrylamide gel electrophoresis. Antibodies used for detection are listed in the Key Resources Table.

Immunofluorescence Microscopy

Indirect immunofluorescence was performed on formaldehyde-fixed cells using the α-Tub1 antibody and Alexa Fluor 594-coupled secondary antibody listed in the Key Resources Table. Cells were counterstained with the DNA binding dye 4',6-diamidino-2-phenylindole (DAPI). Fluorescent images were acquired using an Axioplan 2 imaging microscope (Zeiss) equipped with a 100x (NA = 1.45) Plan-Neofluar objective and an ORCA-ER camera (Hamamatsu). Spindles < 2 μm in length were counted as short (G2/M) spindles, spindles that were 2 μm or longer were classified as long (anaphase) spindles.

Stable Isotope Labeling with Amino Acids In Cell Culture (SILAC) and Mass Spectrometry

Cell cultures were grown for > 8 generations in synthetic complete medium containing 0.1 mg/ml of arginine and lysine or [$^{13}C_6$]arginine and [$^{13}C_6$]lysine, respectively (Gruhler et al., 2005; Rose et al., 1990), then synchronized and released. At the time points described in the individual experiments, aliquots of the cultures were retrieved and mixed. Cells were harvested by centrifugation and resuspended in 20% trichloroacetic acid for protein fixation. Following acetone washes, cells were resuspended in lysis buffer (50 mM ammonium bicarbonate, 5 mM EDTA pH 7.5, 8 M urea) and opened by glass bead breakage. The protein extract was cleared by centrifugation.

Sample Preparation for Mass Spectrometry

1 mg of protein sample was reduced by 5 mM dithiothreitol (DTT) (56°C, 25 min), alkylated with 10 mM iodoacetamide (room temperature, 30 min, dark) and quenched with 7.5 mM DTT. Samples were then diluted with 50 mM ammonium bicarbonate to reduce the urea concentration to < 2 M, prior to trypsin digestion (37°C, overnight). Peptides were then desalted using a C_{18} SepPak Lite (130 mg bed volume) under vacuum and dried. To ensure complete digestion, peptides were further digested using Lys-C in 10% acetonitrile, 50 mM ammonium bicarbonate (37°C, 2 h), followed by trypsin digestion (37°C, overnight). Digested proteins were then desalted again and dried.

Phosphopeptide enrichment using titanium dioxide (TiO_2) was carried out as follows. Dried peptide mixtures were re-suspended in 1 M glycolic acid + 80% acetonitrile + 5% trifluoroacetic acid, sonicated (10 min) and added to titanium dioxide beads (5:1 (w/w) beads:protein). The beads were washed using 80% acetonitrile + 1% trifluoroacetic acid, followed by 10% acetonitrile + 0.2% trifluoroacetic acid, and dried under vacuum centrifugation. Phosphopeptides were eluted from the beads by adding 1% ammonium hydroxide followed by 5% ammonium hydroxide, and dried by vacuum centrifugation. Dried phosphopeptides were re-suspended in 100 μL of 1% trifluoroacetic acid and sonicated (15 min). A C_{18} membrane was packed into a 200 μL pipette tip and washed using methanol and equilibrated with 1% trifluoroacetic acid. The peptides were loaded onto the Stage Tip and washed with 1% trifluoroacetic acid followed by elution with 80% acetonitrile + 5% trifluoroacetic acid. The eluted peptides were again dried by vacuum centrifugation.

An LTQ-Orbitrap Velos was used for data acquisition. Phosphopeptide mixtures were re-suspended in 35 μL 0.1% trifluoroacetic acid and injected three times (10 μL per injection). Each run consisted of a 3 hr gradient elution (75 μm × 50 cm C_{18} column) with one activation method per run: collision induced dissociation (CID), multi-stage activation (MSA) and higher energy collision dissociation (HCD).

Processing and Analysis of Mass Spectrometry Data

MaxQuant (version 1.3.0.5) was used for all data processing. The data were searched against a UniProt extracted *S. cerevisiae* proteome FASTA file amended to include common contaminants. A decoy database containing reverse sequences was used to estimate false discovery rates and set the false discovery rate at 1%. Default MaxQuant parameters were used with the following adjustments: Phospho(STY) was added as a variable modification, ‘Filter labeled amino acids’ was deselected, re-quantify was selected with the instruction to keep low-scoring versions of identified peptides within parameter groups and match between runs was selected. MaxQuant output files were imported into Perseus (version 1.4.0.2) and the normalized heavy-to-light (H:L) ratios were used for all subsequent analyses. Data were also imported into R for statistical testing and data visualization.

QUANTIFICATION AND STATISTICAL ANALYSIS

2D Annotation Enrichment Analysis, Sequence Logos, and the “Lateness” of Threonine Phosphorylation

2D annotation enrichment analysis (Cox and Mann, 2012) was performed in Perseus, using $p < 0.02$ in the adapted Wilcoxon Mann-Whitney test as cutoff for flagging up enrichment. Sequence logos were created using WebLogo (Crooks et al., 2004) and iceLogo (Colaert et al., 2009). In the time-course experiment to follow protein phosphorylation during progression from G1 into mitosis, peptides were included in the analysis if they were identified in at least 8 out of the 10 time points. The conclusion that threonine phosphorylation is a late event in the cell cycle was tested using a χ^2-test across the time categories presented in Figure 3, with ‘late’ compared to the other categories and corrected for multiple testing.

SILAC Experimental Design and Evaluation whether PP2A^{Cdc55} Preferentially Affects Threonines

For the experiment in Figure 2, comparing phosphosite abundance in the presence and absence of PP2A^{Cdc55}, *S. cerevisiae* strains were grown in ‘heavy’ or ‘light’ SILAC medium and mixtures were prepared according an experimental design table, shown as Table S3. Each cell in the table represents a possible SILAC mixture, with its composition shown in the table margin. The six experimental mixtures are highlighted in green, whereas the six control mixtures are highlighted in yellow. The experimental mixtures in which the *cdc55Δ* strain was grown in ‘heavy’ and the wild-type in ‘light’ medium were considered the forward (F) conditions. The mixtures in which the *cdc55Δ* strain was grown in ‘light’ and the wild-type in ‘heavy’ medium were considered the reverse (R) conditions. The same strain was used in the ‘heavy’ and ‘light’ conditions for each of the control mixtures that are shown in the table diagonal. The expected heavy/light (H/L) ratios for phosphorylation sites in the control mixtures are 1.0 (0.0 in a $\log_2$ scale) and the ratio variability is a measure of the reproducibility of the experiment. The total number of phosphorylation sites identified (1% FDR) in the 12 SILAC experiments was 5949. The total number of phosphorylation sites quantified for each individual mixture is shown in each cell of Table S3, as well as the number of quantified sites that contained a phosphorylated serine or threonine within a full Cdk consensus motif (S/TPxK/R).

To assess the reproducibility of the experiments, the SILAC ratios of each phosphorylation site in the control experiments were plotted against the intensities in a $\log_2$ ratio versus $\log_{10}$ intensity plot. For each of the control experiments, over 90% of the data points were contained within a two-fold change interval (Figure S7).

Next, the phosphoproteomes of the *cdc55Δ* and wild-type strains were compared in cells synchronized in G1, S and G2. Ratio versus intensity plots consistently displayed an enrichment of Cdk consensus motif phosphorylation on threonine-containing sites but not, or only to a lesser extent, on Cdk consensus motifs containing serines. To test the significance of increased Cdk threonine site phosphorylation, we generated cumulative frequency graphs in which all sites are ordered as a function of their change between the ‘heavy’ and ‘light’ samples. This confirmed the enrichment of Cdk consensus motif phosphorylation on threonines (Figure S8). As expected, this effect was opposite in forward and reverse experiments whereas little to no effect was observed in the control mixtures. The p values of Wilcoxon-Mann-Whitney tests, indicating whether the category is enriched at high or low values, revealed highly significant enrichment of Cdk threonine site phosphorylation in the absence of PP2A^{Cdc55} (Figure S8).

DATA AND SOFTWARE AVAILABILITY

Abridged tables of the mass spectrometry data are contained in Data S1, S2, and S3. The accession number for the full mass spectrometry proteomics data reported in this paper is ProteomeXchange: PXD004461 (Vizcaíno et al., 2016; http://www.ebi.ac.uk/prode/archive/). Raw data, all MaxQuant output files, and MaxQuant output Phospho(STY) have been deposited.

Molecular Cell, Volume *65*

Supplemental Information

PP2A^{Cdc55} Phosphatase Imposes Ordered Cell-Cycle Phosphorylation by Opposing Threonine Phosphorylation

Molly Godfrey, Sandra A. Touati, Meghna Kataria, Andrew Jones, Ambrosius P. Snijders, and Frank Uhlmann

Figure S1

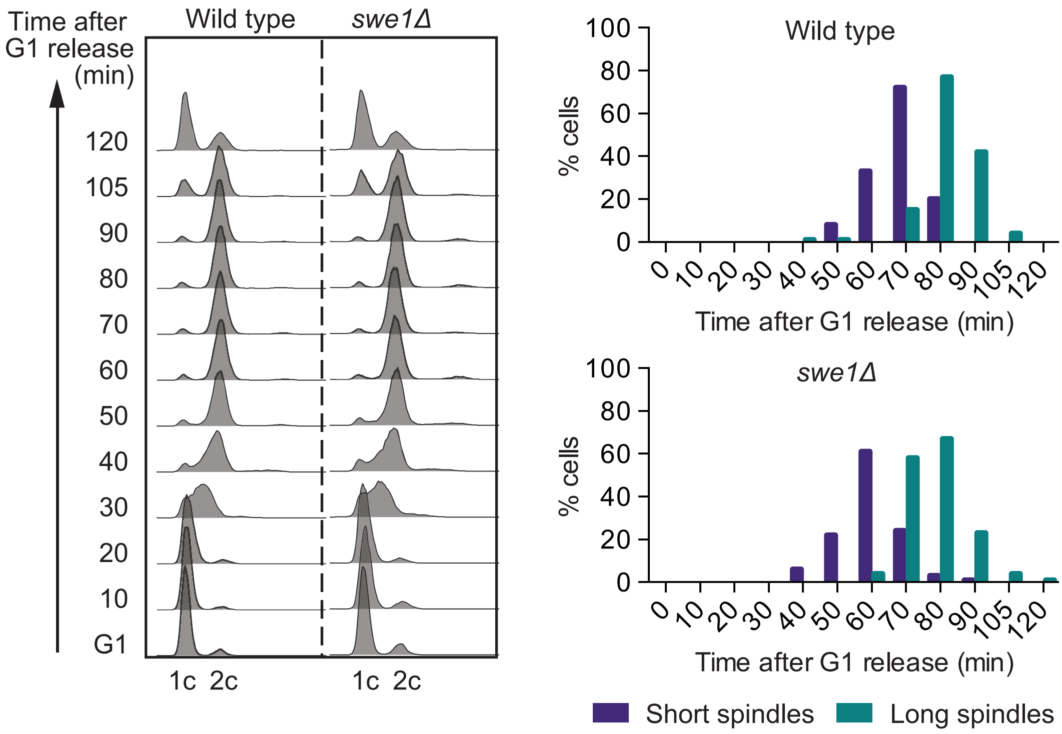

Time after G1 release (min)
Wild type
swe1Δ
120
105
90
80
70
60
50
40
30
20
10
G1
1c 2c
1c 2c
Wild type
% cells
100
80
60
40
20
0
0 10 20 30 40 50 60 70 80 90 105 120
Time after G1 release (min)
swe1Δ
% cells
Time after G1 release (min)
Short spindles
Long spindles

Figure S2

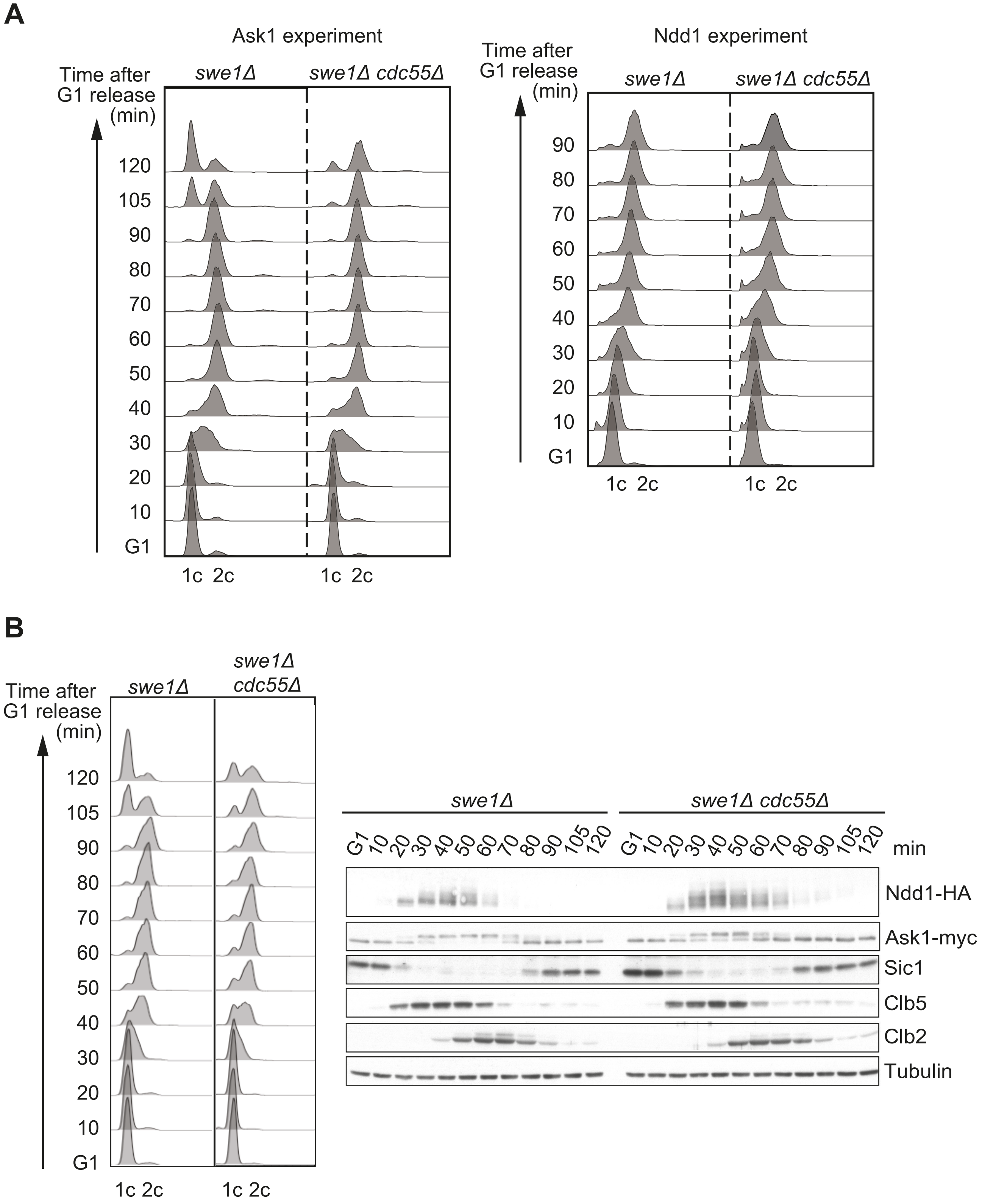
A
Ask1 experiment
Time after G1 release (min)
swe1Δ
swe1Δ cdc55Δ
120
105
90
80
70
60
50
40
30
20
10
G1
1c 2c
1c 2c
Ndd1 experiment
Time after G1 release (min)
swe1Δ
swe1Δ cdc55Δ
90
80
70
60
50
40
30
20
10
G1
1c 2c
1c 2c
B
Time after G1 release (min)
swe1Δ
swe1Δ cdc55Δ
120
105
90
80
70
60
50
40
30
20
10
G1
1c 2c
1c 2c
swe1Δ
swe1Δ cdc55Δ
G1 10 20 30 40 50 60 70 80 90 105 120
G1 10 20 30 40 50 60 70 80 90 105 120
min
Ndd1-HA
Ask1-myc
Sic1
Clb5
Clb2
Tubulin

Figure S3

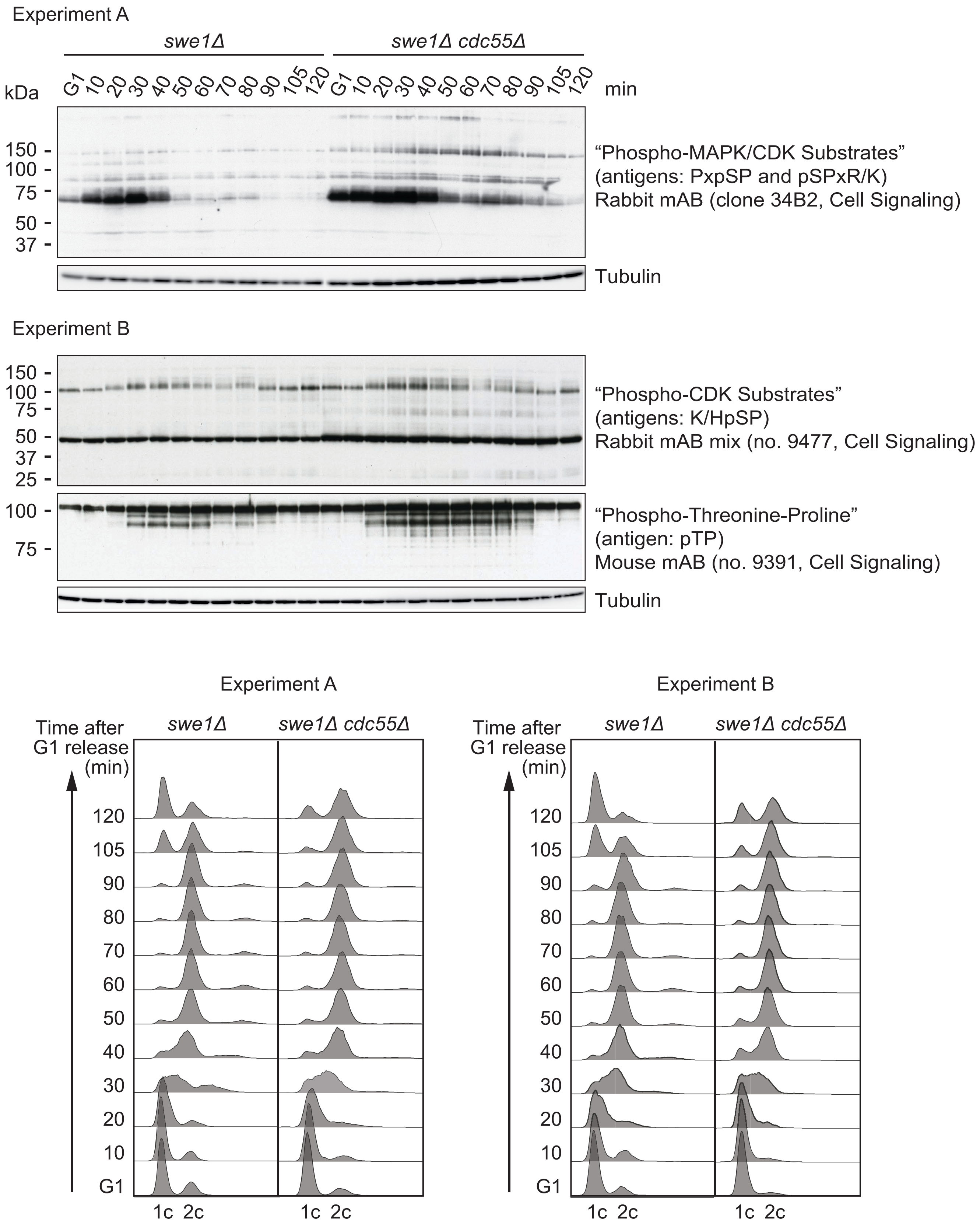

Experiment A
swe1Δ
swe1Δ cdc55Δ
kDa
G1 10 20 30 40 50 60 70 80 90 105 120 G1 10 20 30 40 50 60 70 80 90 105 120 min
150
100
75
50
37
"Phospho-MAPK/CDK Substrates"
(antigens: PxpSP and pSPxR/K)
Rabbit mAB (clone 34B2, Cell Signaling)
Tubulin
Experiment B
150
100
75
50
37
25
"Phospho-CDK Substrates"
(antigens: K/HpSP)
Rabbit mAB mix (no. 9477, Cell Signaling)
100
75
"Phospho-Threonine-Proline"
(antigen: pTP)
Mouse mAB (no. 9391, Cell Signaling)
Tubulin
Experiment A
Time after G1 release (min)
swe1Δ
swe1Δ cdc55Δ
120
105
90
80
70
60
50
40
30
20
10
G1
1c 2c
1c 2c
Experiment B
Time after G1 release (min)
swe1Δ
swe1Δ cdc55Δ
120
105
90
80
70
60
50
40
30
20
10
G1
1c 2c
1c 2c

Figure S4

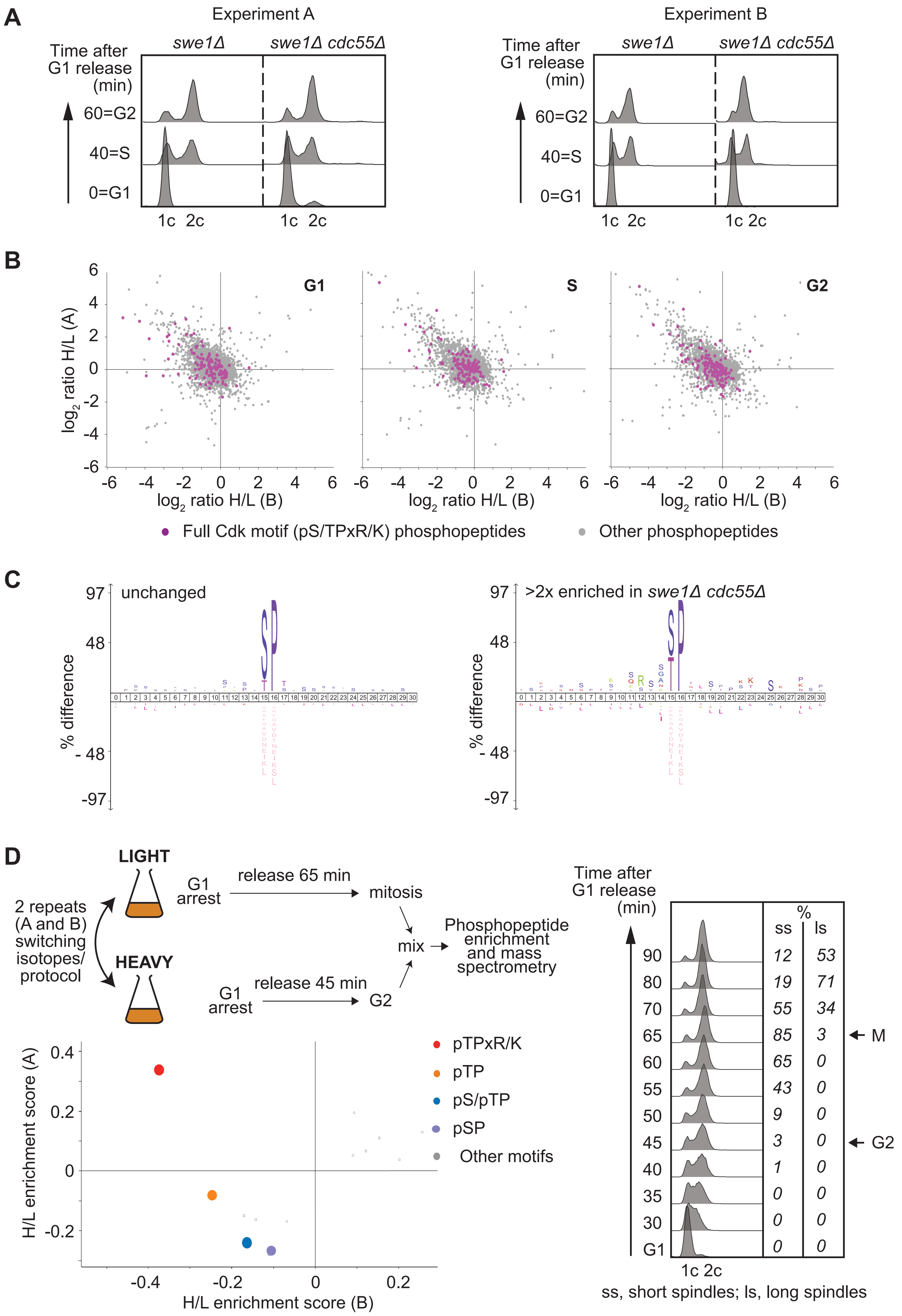
A
Experiment A
Experiment B
swe1Δ
swe1Δ cdc55Δ
Time after G1 release (min)
60=G2
40=S
0=G1
1c 2c
B
G1
S
G2
log2 ratio H/L (A)
log2 ratio H/L (B)
Full Cdk motif (pS/TPxR/K) phosphopeptides
Other phosphopeptides
C
unchanged
>2x enriched in swe1Δ cdc55Δ
% difference
97
48
- 48
-97
D
LIGHT
HEAVY
2 repeats (A and B) switching isotopes/ protocol
G1 arrest
release 65 min
mitosis
release 45 min
G2
mix
Phosphopeptide enrichment and mass spectrometry
H/L enrichment score (A)
H/L enrichment score (B)
pTPxR/K
pTP
pS/pTP
pSP
Other motifs
Time after G1 release (min)
%
ss
ls
90 12 53
80 19 71
70 55 34
65 85 3
60 65 0
55 43 0
50 9 0
45 3 0
40 1 0
35 0 0
30 0 0
G1 0 0
M
G2
1c 2c
ss, short spindles; ls, long spindles

Figure S5

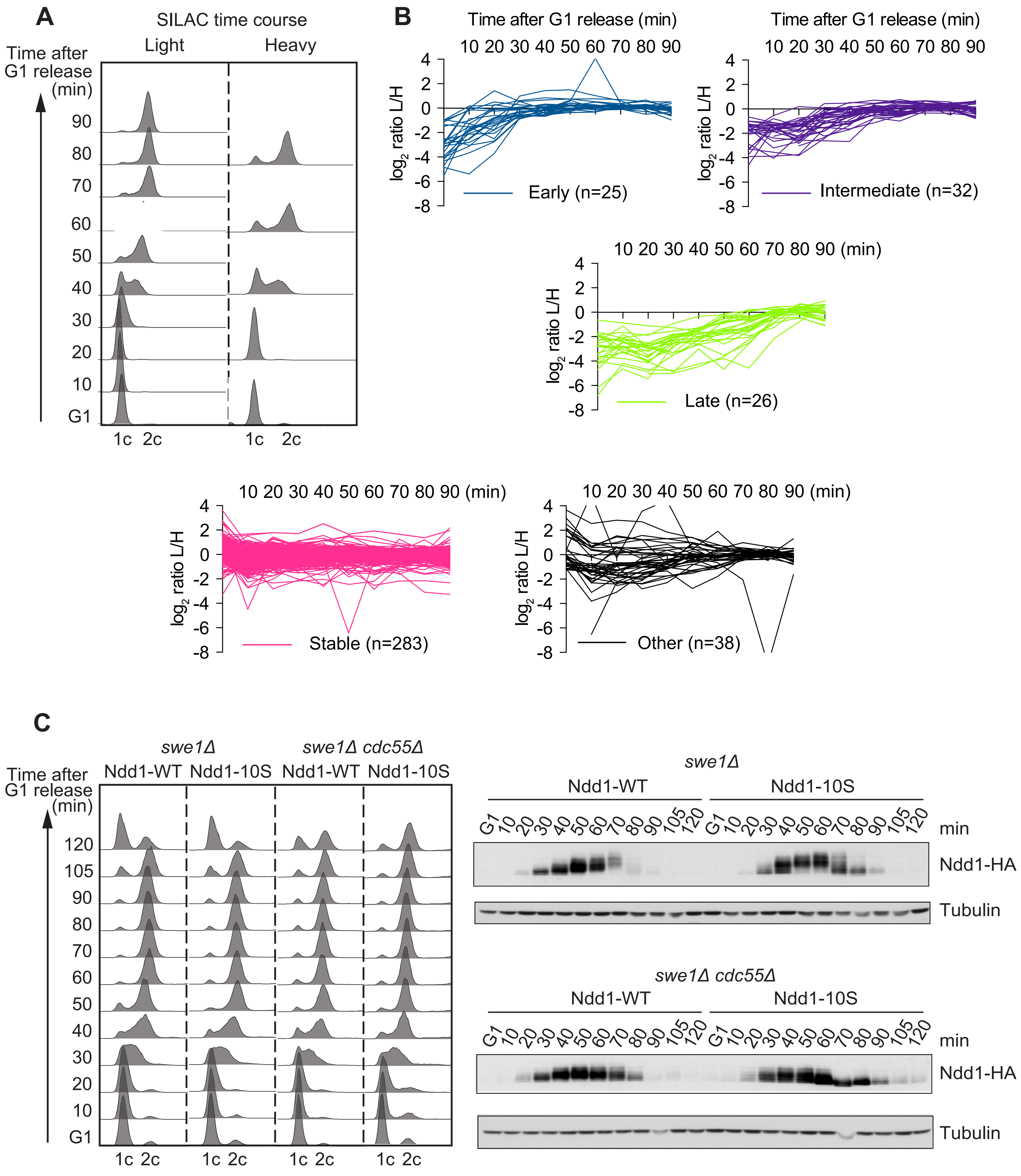
A
SILAC time course
Light
Heavy
Time after G1 release (min)
90
80
70
60
50
40
30
20
10
G1
1c 2c
1c 2c
B
Time after G1 release (min)
10 20 30 40 50 60 70 80 90
log2 ratio L/H
Early (n=25)
Intermediate (n=32)
10 20 30 40 50 60 70 80 90 (min)
Late (n=26)
Stable (n=283)
Other (n=38)
C
swe1Δ
swe1Δ cdc55Δ
Ndd1-WT
Ndd1-10S
Time after G1 release (min)
120
105
G1 10 20 30 40 50 60 70 80 90 105 120
min
Ndd1-HA
Tubulin

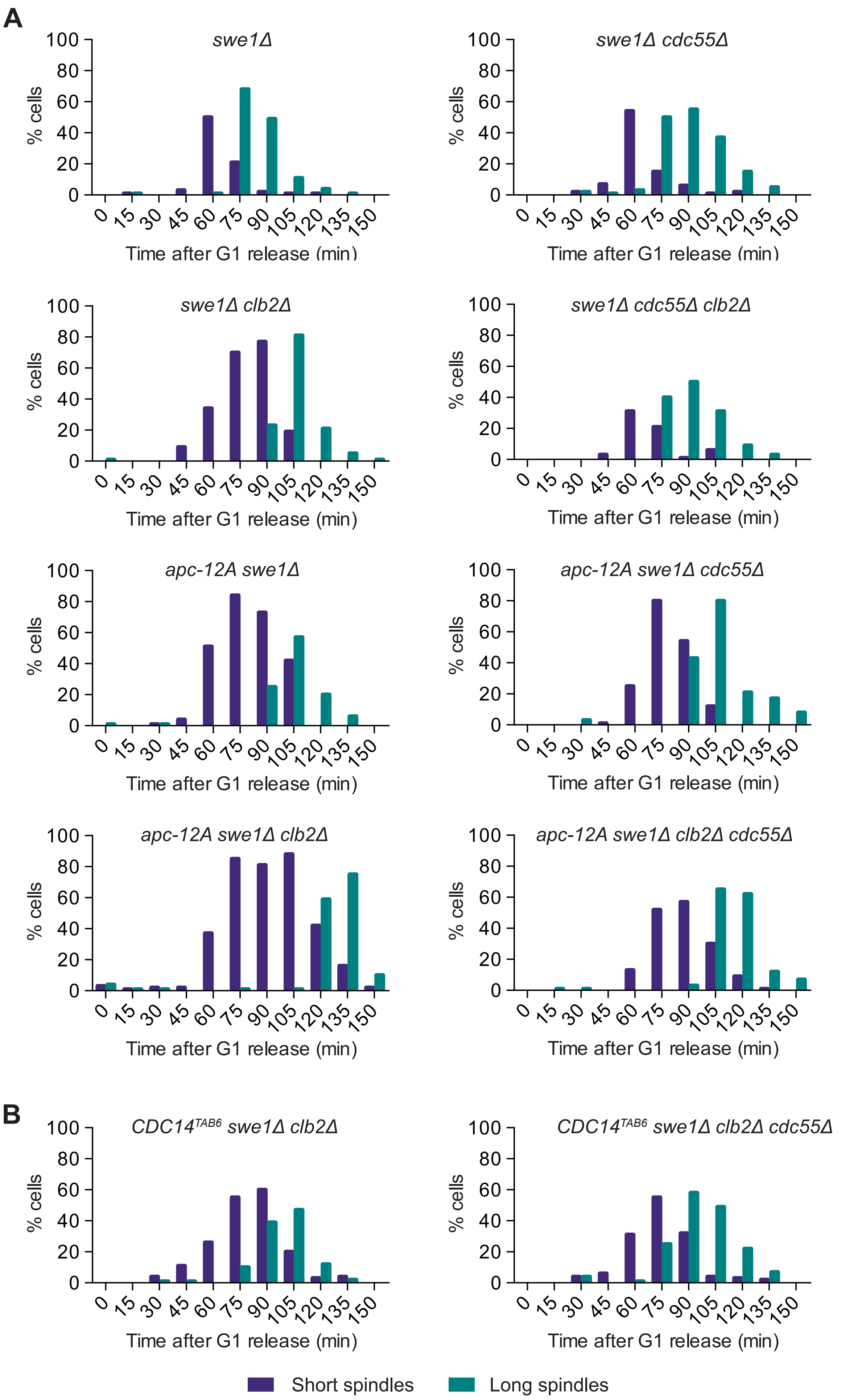
A
swe1Δ
swe1Δ cdc55Δ
swe1Δ clb2Δ
swe1Δ cdc55Δ clb2Δ
apc-12A swe1Δ
apc-12A swe1Δ cdc55Δ
apc-12A swe1Δ clb2Δ
apc-12A swe1Δ clb2Δ cdc55Δ
B
CDC14TAB6 swe1Δ clb2Δ
CDC14TAB6 swe1Δ clb2Δ cdc55Δ
% cells
Time after G1 release (min)
Short spindles
Long spindles

Figure S7

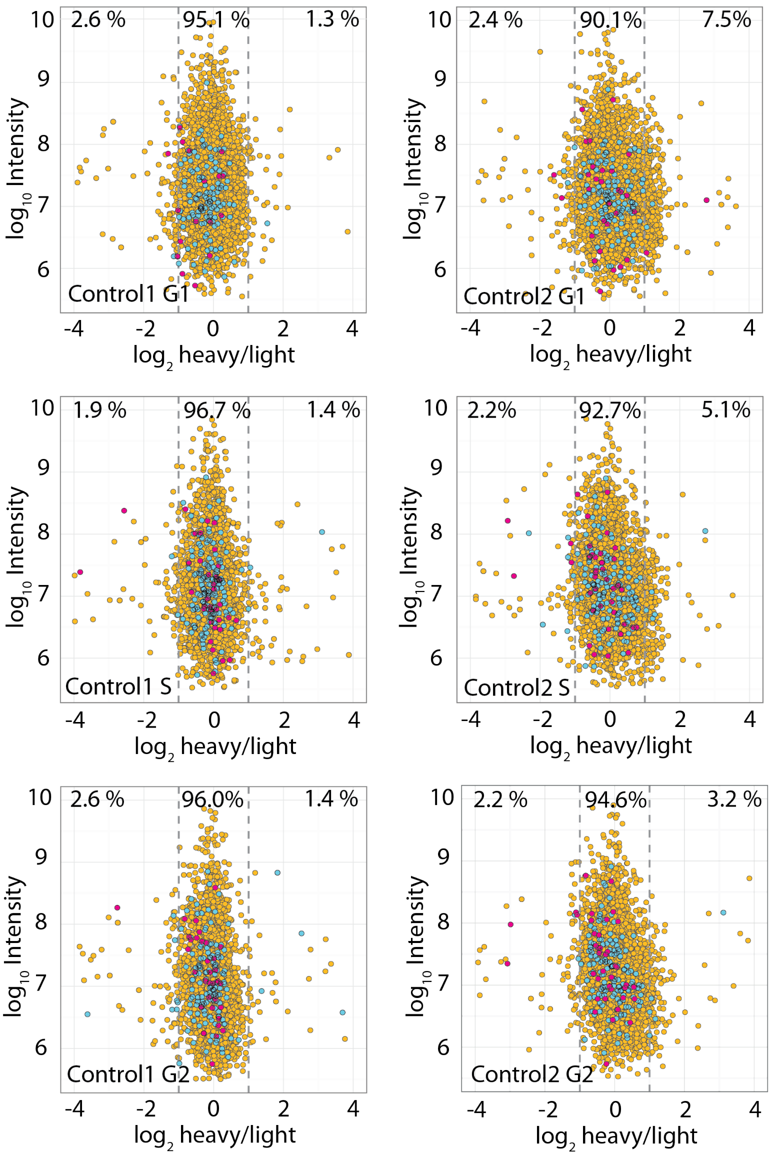

2.6 %
95.1 %
1.3 %
Control1 G1
2.4 %
90.1%
7.5%
Control2 G1
1.9 %
96.7 %
1.4 %
Control1 S
2.2%
92.7%
5.1%
Control2 S
2.6 %
96.0%
1.4 %
Control1 G2
2.2 %
94.6%
3.2 %
Control2 G2
log10 Intensity
log2 heavy/light

Figure S8

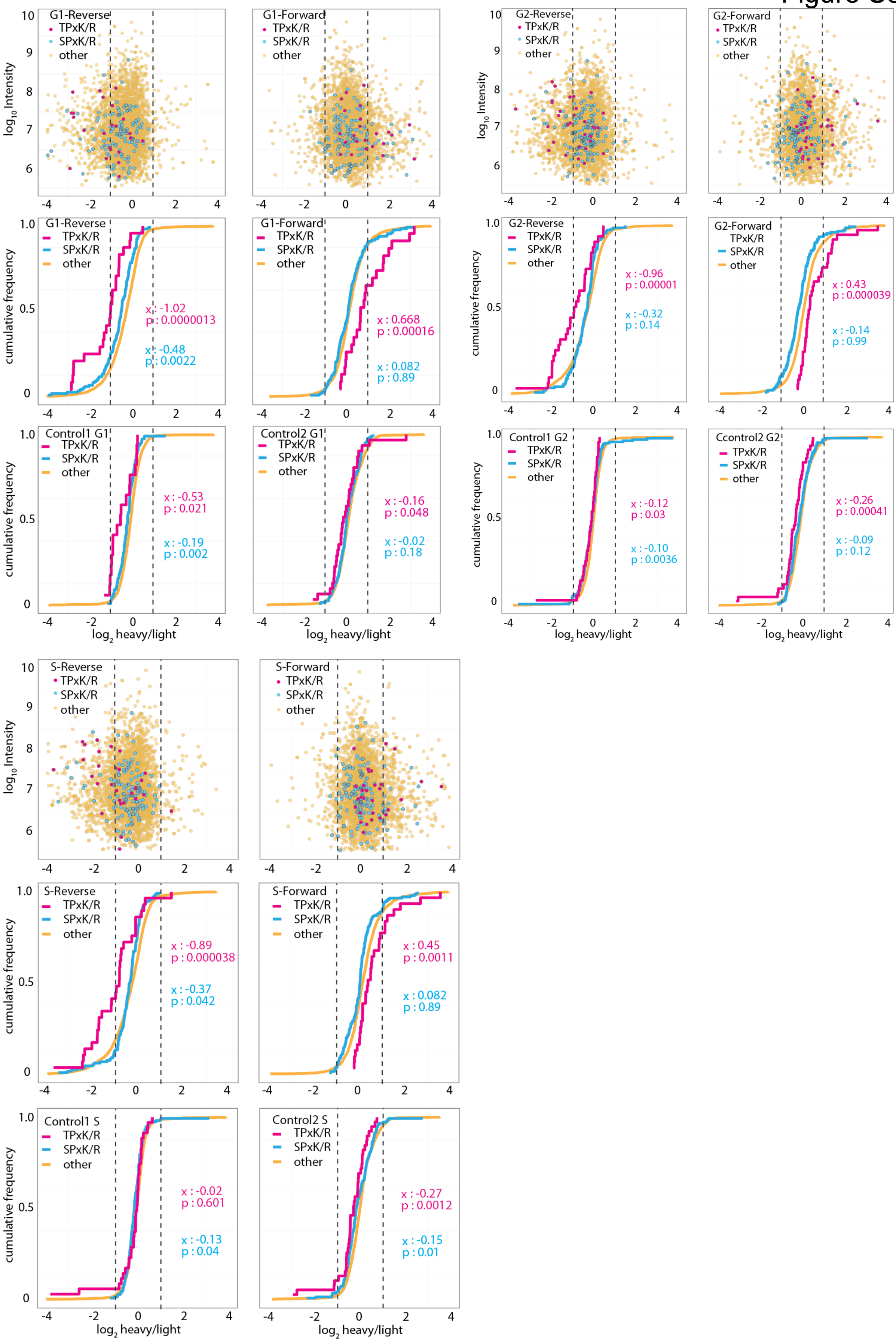

G1-Reverse
TPxK/R
SPxK/R
other
x : -1.02
p : 0.0000013
x : -0.48
p : 0.0022
G1-Forward
x : 0.668
p : 0.00016
x : 0.082
p : 0.89
G2-Reverse
x : -0.96
p : 0.00001
x : -0.32
p : 0.14
G2-Forward
x : 0.43
p : 0.000039
x : -0.14
p : 0.99
Control1 G1
x : -0.53
p : 0.021
x : -0.19
p : 0.002
Control2 G1
x : -0.16
p : 0.048
x : -0.02
p : 0.18
Control1 G2
x : -0.12
p : 0.03
x : -0.10
p : 0.0036
Ccontrol2 G2
x : -0.26
p : 0.00041
x : -0.09
p : 0.12
S-Reverse
x : -0.89
p : 0.000038
x : -0.37
p : 0.042
S-Forward
x : 0.45
p : 0.0011
x : 0.082
p : 0.89
Control1 S
x : -0.02
p : 0.601
x : -0.13
p : 0.04
Control2 S
x : -0.27
p : 0.0012
x : -0.15
p : 0.01
log10 Intensity
cumulative frequency
log2 heavy/light

SUPPLEMENTAL FIGURE LEGENDS

Figure S1. Comparison of cell cycle progression between *swe1Δ* and wild type cells, Related to Figure 1

swe1Δ and wild type cells were arrested in G1 by pheromone α-factor treatment, released to progress through a synchronous cell cycle before re-arrest in the next G1 phase by α-factor re-addition. Cell cycle progression was monitored by FACS analysis of DNA content and by scoring the fraction of cells with short (<2 μm) and long (≥2 μm) spindles. This revealed that wild type and *swe1Δ* cells progress through S phase at similar rates, but that *swe1Δ* cells enter mitosis and progress into anaphase approximately 10-15 minutes earlier than wild type cells, consistent with published observations (Wang and Burke, 1997).

Figure S2. Additional FACS analyses and repeat of the Ndd1 phosphorylation analysis including internal timing controls, Related to Figure 1

(A) FACS profiles to monitor cell cycle progression during the experiments, shown in Figure 1B, in which the kinetics of Ask1 and Ndd1 phosphorylation were analyzed by western blotting.

(B) Repeat of the Ndd1 phosphorylation timecourse analysis. As in Figure 1B, but Ask1 phosphorylation and the levels of Sic1, Clb5 and Clb2 were also analyzed and served as internal timing controls. This confirmed advanced Ndd1 phosphorylation, relative to Ask1, in cells lacking $PP2A^{Cdc55}$. Tubulin served as a loading control.

Figure S3. Comparison of cell cycle-regulated Cdk phosphorylation in the presence and absence of $PP2A^{Cdc55}$, using antibodies raised against phosphorylated Cdk consensus peptides, Related to Figure 1

Cells were arrested in G1 by pheromone α-factor treatment, released to progress through a synchronous cell cycle, before re-arrest in the next G1 phase by α-factor re-addition. Cell extracts were prepared at the indicated times and analyzed by western blotting using the indicated antibodies. While these antibodies were raised against either phosphoserine or phosphothreonine-containing peptides, their ability to discriminate between phosphoserine and phosphothreonine is not known. Tubulin served as a loading control. Cell cycle stages were confirmed by FACS analysis of DNA content. A discernable cell cycle advance and intensity increase of bands reactive against all three antibodies is apparent in the absence of $PP2A^{Cdc55}$.

Figure S4. Analysis of the $PP2A^{Cdc55}$-dependent phosphoproteomes at three timepoints during synchronous cell cycle progression and a G2/M comparison of the phosphoproteomes in wild type cells, Related to Figure 2

(A) FACS analysis of DNA content from the two inverse SILAC experiments documented in Figure 2, illustrating the cell cycle stage at which the timepoints were taken. Immunofluorescence samples were also analyzed to confirm that the G2 sample was taken before mitotic spindle formation (not shown).

(B) The heavy/light (H/L) ratio of all phosphopeptides in the 2 repeats, (A) and (B) as in Figure 2, are shown. Full Cdk consensus motif-containing phosphopeptides are highlighted. Their increased levels in the absence of $PP2A^{Cdc55}$ become apparent in the upper left quadrant of the diagram.

(C) Sequence logos of pSP/pTP-sites, enriched greater than two-fold in the absence of $PP2A^{Cdc55}$, or unchanged, were prepared as in Figure 2D, but the iceLogo algorithm (Colaert et al., 2009) was used that takes the species-specific probability of amino acid occurrence into account.

(D) Phosphoproteome comparison between G2 phase and mitosis. A schematic of the SILAC experiment is shown, indicating the times when G2 and mitosis samples were taken after the staggered synchronous release of cultures grown in light and heavy amino acids. Cell cycle progression was monitored by FACS analysis of DNA content and by scoring the fraction of cells with short and long spindles. 3,124 phosphopeptides were reproducibly identified in both cultures in both repeats. A 2D annotation enrichment plot is shown of motifs preferentially phosphorylated in mitosis. Other phosphorylation consensus sites are indicated in grey. Compare Figure 2C for details. The strongest enrichment in mitosis, compared to G2, was observed for the threonine-directed full Cdk consensus motif pTPxR/K. See also Dataset 3 for the abridged mass spectrometry data.

Figure S5. Phosphoproteome analysis during synchronous cell cycle progression and the effect of PP2A^{Cdc55} on Ndd1-10S phosphorylation, Related to Figures 3 and 4

(A) FACS analysis of DNA content to monitor cell cycle progression during the SILAC time course experiment shown in Figure 3.

(B) The traces of all pSP and pTP sites are shown, subdivided by whether they reached their maximal phosphorylation at early (10 – 30 minutes, G1-S), intermediate (40 – 60 minutes, S-G2) or late (60 – 90 minutes, mitosis) timepoints. Stable phosphopeptides are also shown, as well as those in the 'other' category.

(C) PP2A^{Cdc55} has little influence on the phosphorylation timing of Ndd1-10S. Strains of the indicated genotypes, expressing Ndd1 or Ndd1-10S, progressed synchronously through the cell cycle following α-factor arrest and release. The Ndd1 phosphorylation status was analyzed by western blotting. Tubulin served as a loading control.

Figure S6. APC and Net1 phosphorylation only partly explain the role of PP2A^{Cdc55} in ordering S-phase and mitosis, Related to Figure 5

Cells of the indicated genotypes were arrested in G1 by pheromone α-factor treatment, released to progress through a synchronous cell cycle before re-arrest in the next G1 phase by α-factor re-addition. Cell cycle progression was monitored by scoring the percentage of cells with short or long spindles.

(A) The mitotic delay caused by the *apc-12A* mutations, even more pronounced in the *clb2Δ* background, was substantially reduced by *cdc55* deletion, indicating that mitotic control exerted by PP2A^{Cdc55} acts on targets in addition to the APC.

(B) Similarly, *CDC14*^{TAB6} was less able than *cdc55Δ* to advance mitosis in the *clb2Δ* background, indicating that PP2A^{Cdc55} acts on targets in addition to the Cdc14 inhibitor Net1.

Figure S7. Intensity plots of the SILAC ratios in the six control experiments, Related to the STAR*Methods

The $\log_2$ ratio versus $\log_{10}$ intensity of each phosphorylation site was plotted for each of the six control experiments in which the same strains were labeled with light or heavy amino acids at three cell cycle stages. The two-fold change intervals are indicated by the dashed lines and the percentages of phosphorylation sites contained within this interval is displayed in the panels. Each datapoint represents a quantified phosphorylation site. Phosphorylated TPxK/R sites are indicated in pink, SPxK/R sites in blue.

Figure S8. Cumulative frequency graphs comparing the differences in threonine and serine phosphorylation in the absence of PP2A^{Cdc55}, Related to the STAR*Methods

In these cumulative frequency graphs, all phosphrylation sites are ordered as a function of their change between the 'heavy' and 'light' samples. The median $\log_2$ SILAC ratio difference 'x' for both TPxK/R and SPxK/R categories, compared to all other phosphosites is given. Also given is the p-value of a Wilcoxon-Mann-Whitney test, indicating whether the category is enriched at high or low values, as determined by a 1D annotation enrichment test (Cox and Mann, 2012). This revealed highly significant enrichment of Cdk threonine site phosphorylation in the absence of PP2A^{Cdc55}.

Dataset 1. Abridged mass spectrometry data comparing phosphosite abundance between a wild type and a *cdc55Δ* strain at three cell cycle stages, G1, S and G2, Related to Figure 2.

Dataset 2. Abridged mass spectrometry data of the experiment to follow phosphosite abundance during synchonous cell cycle progression of a wild type strain, Related to Figure 3.

Dataset 3. Abridged mass spectrometry data comparing the phosphosite abundance in a wild type strain between G2 and M, Related to Figure S4D

SUPPLEMENTAL TABLES

Table S1 Overview of peptide counts in the phosphoproteome datasets, Related to Figures 2 and 3

Figure 2

Top left quadrant	Peptides	Proteins
G1	1286	588
S	1286	589
G2	1305	585

Others	Peptides	Proteins
G1	2208	924
S	1962	838
G2	1998	868

	Number of phosphopeptides			
	G1	S	G2	Total
pTPxK/R	19	22	27	35
pTP	223	206	208	320
pS/TP	914	846	851	1209
pSP	691	640	634	889
pSPxR/K	106	121	127	143
pS/TPxR/K	125	145	155	178
Aurora	--	49	50	61

	Phosphopeptides			
	All	S/TP-containing	enriched >2X	S/TP enriched >2X
G1	3122	914	192	117
S	2901	846	210	28
G2	2867	851	237	143
Total	4589	1203		197

Figure 3

	Peptides	Proteins	pSP peptides	pTP peptides
All	405	298		
Early	25	16	22	3
Intermediate	32	30	26	6
Late	26	22	12	14
Stable	295	209	230	53
Oher	27	21	30	8

Table S2 Yeast strains used in this study, Related to the STAR*Methods section.

Strain name	Genotype	Background	Source
**5059**	*MATa ASK1-HA3::TRP1 swe1Δ::HIS3*	W303	This study
**5060**	*MATa ASK1-HA3::TRP1 swe1Δ::HIS3 cdc55Δ::LEU2*	W303	This study
**5057**	*MATa SLI15-HA3::TRP1 swe1Δ::HIS3*	W303	This study
**5058**	*MATa SLI15-HA3::TRP1 swe1Δ::HIS3 cdc55Δ::LEU2*	W303	This study
**5049**	*MATa NDD1-HA3::TRP1 swe1Δ::HIS3*	W303	This study
**5050**	*MATa NDD1-HA3::TRP1 swe1Δ::HIS3 cdc55Δ::LEU2*	W303	This study
**5048**	*MATa NDD1-HA3::TRP1*	W303	This study
**5231**	*MATa NDD1-HA3::TRP1 swe1Δ::HIS3 ASK1-myc18::URA3*	W303	This study
**5232**	*MATa NDD1-HA3::TRP1 swe1Δ::HIS3 cdc55Δ::LEU2 ASK1-myc18::URA3*	W303	This study
**2369 (YAL6B)**	*MATa arg4Δ(YHR018C)::kan^R lys1Δ(YIR034C)::kan^R*	S288C	Gruhler *et al.* (2005)
**4639**	*MATa arg4Δ(YHR018C)::kan^R lys1Δ(YIR034C)::kan^R swe1Δ::HIS3*	S288C	This study
**4640**	*MATa arg4Δ(YHR018C)::kan^R lys1Δ(YIR034C)::kan^R swe1Δ::HIS3 cdc55Δ::LEU2*	S288C	This study
**5230**	*MATa NDD1-HA3::TRP1 ASK1-myc18::URA3*		
**5233**	*MATa NDD1-10S-HA3::TRP1 ASK1-myc18::URA3*		
**5126**	*MATa NDD1-10S-HA3::TRP1*	W303	This study
**5127**	*MATa NDD1-10S-HA3::TRP1 swe1Δ::HIS3*	W303	This study
**5128**	*MATa NDD1-10S-HA3::TRP1 swe1Δ::HIS3 cdc55Δ::LEU2*	W303	This study
**5093**	*MATa NDD1-HA3::TRP1 swe1Δ::HIS3 clb2Δ::URA3*	W303	This study
**5095**	*MATa NDD1-HA3::TRP1 swe1Δ::HIS3 cdc55Δ::LEU2 clb2Δ::URA3*	W303	This study
**5076**	*MATa swe1Δ::HIS3 CDC55-PK3::TRP1 PPH21-PK3::LEU2*	W303	This study
**5067**	*MATa swe1Δ::HIS3 GAL1pr-CDC55-PK3::LEU2 (x1)*	W303	This study
**5068**	*MATa swe1Δ::HIS3 GAL1pr-CDC55-PK3::LEU2 (x2)*	W303	This study
**5078**	*MATa swe1Δ::HIS3 GAL1pr-CDC55-PK3::LEU2 (x2) GAL10pr-GAL4 GAL1pr-PPH21-PK3::ADE2*	W303	This study
**5065 (ADR6624)**	*MATa cdc16-6A::TRP1 cdc27-5A::kan^R cdc23-A::hyg^R bar1Δ*	W303	Gift from A. Rudner
**5071**	*MATa cdc16-6A::TRP1 cdc27-5A::kan^R cdc23-A::hyg^R bar1Δ swe1Δ::HIS3*	W303	This study
**5072**	*MATa cdc16-6A::TRP1 cdc27-5A::kan^R cdc23-A::hyg^R bar1Δ swe1Δ::HIS3 cdc55Δ::LEU2*	W303	This study
**5073**	*MATa cdc16-6A::TRP1 cdc27-5A::kan^R cdc23-A::hyg^R bar1Δ swe1Δ::HIS3 clb2Δ::URA3*	W303	This study
**5074**	*MATa cdc16-6A::TRP1 cdc27-5A::kan^R cdc23-A::hyg^R bar1Δ swe1Δ::HIS3 cdc55Δ::LEU2 clb2Δ::URA3*	W303	This study
**5097**	*MATa CDC14^{TAB6}-PK6::TRP1 swe1Δ::HIS3 clb2Δ::URA3*	W303	This study
**5098**	*MATa CDC14^{TAB6}-PK6::TRP1 swe1Δ::HIS3 cdc55Δ::LEU2 clb2Δ::URA3*	W303	This study

Table S3 Experimental design table for the experiment in Figure 2, comparing phosphosite abundance in the presence and absence of PP2A^{Cdc55}. The total number of phosphorylation sites quantified for each individual mixture is shown in each cell, as well as the number of quantified sites that contained a phosphorylated serine or threonine within a full Cdk consensus motif (S/TPxK/R), Related to the STAR*Methods section.

			heavy					
			WT			*cdc55Δ*		
			G1	S	G2	G1	S	G2
light	WT	G1	**Control1 G1**<br>total: 4240<br>SPxK/R: 122<br>TPxK/R: 17			**G1-F**<br>total: 4190<br>SPxK/R: 135<br>TPxK/R: 23		
		S		**Control1 S**<br>total: 4117<br>SPxK/R: 157<br>TPxK/R: 35			**S-F**<br>total: 4000<br>SPxK/R: 146<br>TPxK/R: 32	
		G2			**Control1 G2**<br>total: 3453<br>SPxK/R: 139<br>TPxK/R: 34			**G2-F**<br>total: 3746<br>SPxK/R: 149<br>TPxK/R: 37
	cdc55Δ	G1	**G1-R**<br>total: 3945<br>SPxK/R: 122<br>TPxK/R: 26			**Control2 G1**<br>total: 4188<br>SPxK/R: 125<br>TPxK/R: 31		
		S		**S-R**<br>total: 3610<br>SPxK/R: 135<br>TPxK/R: 30			**Control2 S**<br>total: 3907<br>SPxK/R: 151<br>TPxK/R: 39	
		G2			**G2-R**<br>total: 3709<br>SPxK/R: 144<br>TPxK/R: 32			**Control2 G2**<br>total: 3904<br>SPxK/R: 153<br>TPxK/R: 40
